# How should overall survival be analysed in randomised clinical trials in cancer if participants receive subsequent treatment lines? A stakeholder consultation

**DOI:** 10.1186/s13063-025-09148-3

**Published:** 2025-10-24

**Authors:** Kara-Louise Royle, Peter Wheatstone, David Meads, Jennifer K. Visser-Rogers, Ian R. White, David A. Cairns

**Affiliations:** 1 Leeds Cancer Research UK Clinical Trials Unit, Leeds Institute of Clinical Trials Research, University of Leeds, Leeds, UK; 2 Patient Partner, Leeds, UK; 3 Academic Unit of Health Economics, Leeds Institute of Health Sciences, University of Leeds, Leeds, UK; 4 Coronado Research, Kent, England; 5 MRC Clinical Trials Unit at UCL, London, UK

**Keywords:** Qualitative analysis, Online questionnaire, Focus group, Overall survival, Randomised controlled trial, Cancer, Subsequent treatment lines

## Abstract

**Background:**

Overall survival is used to assess clinical effectiveness in cancer clinical trials. In practice, it may be influenced by intercurrent events post-randomisation. The decisions made on how to address intercurrent events, change the interpretation of the results.

An example is when participants stop their trial intervention and start subsequent anti-cancer interventions (treatment lines) during trial follow-up. At present, there is no evidence on the views of all stakeholders about this intercurrent event or consensus on how it should be addressed. The aim of this work was to understand the perspectives of all stakeholders and to obtain consensus through a qualitative study to guide future methodological work.

**Methods:**

A modified Rand/UCLA appropriateness method was implemented. Stakeholder views were collected using an online questionnaire and discussed at a focus group. The questionnaire included items on, the different methods for addressing an intercurrent event, data collection following an intercurrent event, statistical assumptions, and data presentation. Analysis was descriptive incorporating a conventional content approach. Consensus was defined a priori.

**Results:**

One hundred three stakeholders (30 statisticians or other data analysts, 6 payers or industry partners, 22 healthcare professionals and 45 patient, carer or members of the public) completed the questionnaire between 3/8/2022 and 30/9/2022. Seventy-nine percent of respondents thought it important to consider the potential effect of subsequent treatment lines.

Consensus was reached on most questionnaire items. Stakeholders agreed that statistical assumptions were applicable only in “Some Scenarios” and that results should be presented using both a visual and summary measure. The focus group discussed different methods for addressing an intercurrent event and items around data collection where consensus was unclear. Seven participants attended (two patients/carers, one healthcare professional, three statisticians and one payer) with K-LR and PW. Attendees agreed that the treatment policy approach should be considered in future work as it was the most realistic, and that data collection was acceptable with informed consent.

**Discussion:**

This work demonstrates that all stakeholder groups are interested in how subsequent treatment lines may impact overall survival and provides evidence on what future methodological work in the area should consider. The next step of this work will investigate whether it is possible to estimate the overall survival treatment effect in a hypothetical scenario where participants who received second-line therapy all received the same second-line therapy. This will aim to complement the existing treatment policy approach and quantify the impact of subsequent treatments.

**Supplementary Information:**

The online version contains supplementary material available at 10.1186/s13063-025-09148-3.

## Introduction

In a confirmatory randomised controlled trial, the endpoint overall survival (OS) is defined as the time from randomisation to death from any cause [1]. It is a definitive endpoint used to assess the clinical effectiveness of experimental interventions for the treatment of cancer.

On analysing OS, like any endpoint, statisticians have to make decisions on how to address post-randomisation events which could affect the interpretation or occurrence of the outcome. These are termed intercurrent events and are defined as part of the estimand framework [2]. The estimand framework is a structured way to pre-specify how a clinical trial will answer a research question. It enables researchers to explicitly document; the population of interest, the interventions being considered in the trial, the outcome of interest to answer the research question, how the outcome will be summarised at the population level, and consideration of any intercurrent events which may affect the interpretation of the outcome. There are five different approaches for addressing intercurrent events. The decision to use any one of these approaches affects the question being asked and therefore, how the results should be interpreted.

The results from the analysis of OS are used, alongside other evidence, in Health Technology Assessments (HTA) to determine whether interventions should be recommended for use in standard of care [3]. If approved, these results, alongside other factors, are then used by patients, carers and clinicians to decide on the best treatment option. Hence the decisions made by the statistician during analysis can impact the choices made by each stakeholder group. It follows that stakeholders should be consulted when statisticians are considering how to address intercurrent events.

An example of an intercurrent event is when trial participants discontinue their trial intervention prior to death and receive subsequent anti-cancer treatment during trial follow-up. This intercurrent event is well known and is acknowledged as a disadvantage of OS in clinical trial recommendations [1]. At present, methodological solutions only exist in the very specific and limited scenario of treatment switching, where patients in the control arm receive the experimental intervention during a later line of treatment [4]. However, a recent literature review showed that the intercurrent event, as a whole, is of interest. The review included 98 clinical effectiveness papers and 59 (60%) of them mentioned subsequent treatments. Of these, seven performed additional analysis for subsequent treatments. The review concluded that the majority of researchers are applying the treatment policy approach to the intercurrent event, i.e. it is considered part of the treatment strategy and there is currently no methodological solution to determine how much of an effect subsequent treatment has on the interpretation of OS [5].

The objectives of this work were to understand to different stakeholder perspectives and experience on the topic and seek consensus through a qualitative study, incorporating an online questionnaire and focus group. Consensus was sought in four different areas to guide the development of a methodological solution to analyse OS when trial participants receive subsequent anti-cancer treatment during trial follow-up, to ensure the solution was informed by stakeholder views.

The consensus exercise primarily aimed to identify the question stakeholders were the most interested in, when they knew that trial participants may have stopped their trial treatment and received subsequent treatment lines during trial follow-up. This was facilitated by considering the different methods available to address intercurrent events (Table 1). Alongside this, the consensus exercise aimed to establish agreement on: what and how data should be collected to answer the question of interest; which statistical assumptions may be appropriate in answering the question; and how the analysis should be presented. Finally, the online questionnaire also sought to collect information on the current practice for the analysis of OS from statisticians and other data analysts.

**Table 1:** Methods of dealing with an intercurrent event

Method of dealing with an intercurrent event	Approach	Impact	Question*
Treatment policy	Intercurrent event is considered part of the treatment strategy		How does the new treatment extend survival compared to the control treatment; even though some participants stopped their trial treatment prior to death?
Hypothetical	The treatment effect for the hypothetical scenario where the intercurrent event is assumed to not have occurred is estimated	Changes the interpretation of the results	How would have the new treatment extended survival compared to the control treatment, if no one stopped their trial treatment prior to death?
Principal stratum	The treatment effect in which the intercurrent event would (or would not) occur is estimated	Subsets the population to only consider those who did (or did not) experience the intercurrent event	How does the new treatment extend survival compared to the control treatment, in participants who only received their trial treatment prior to death?
Composite	The intercurrent event is incorporated into the endpoint definition	Changes the definition of the endpoint	How much longer did participants stay on the experimental treatment compared to the control treatment?
While on treatment/alive	The endpoint prior to treatment discontinuation is of interest	Changes the definition of the endpoint	

^*^Note these questions are as written in the questionnaire for healthcare professionals, payers and industry partners, and statisticians and data analysts. The version for patient, carer or member of the public can be seen in Additional File 3, page 17

## Methods

The qualitative study utilised a modified Rand/UCLA appropriateness method [6], consisting of both an online questionnaire and a focus group (Additional File 7: Supplementary Fig. 1). As a result of the combined approach, both the CHERRIES (Checklist for Reporting Results of Internet E-Surveys) [7] and COREQ (Consolidated criteria for reporting qualitative research) [8] reporting guidelines have been used throughout the manuscript (Additional File 1 and Additional File 2).

### Online questionnaire

#### Development and testing

The target population for the questionnaire were the stakeholders of clinical trials research. The stakeholders were categorised into: patients, carer and the public, health care professionals, statisticians and other data analysts, and payers and industry partners (Table 2).

**Table 2:** The definitions of the stakeholder groups

Stakeholder group	Definition
A member of the public*	For those who have not • Treated patients with cancer • Analysed a cancer clinical trial • Discussed whether a treatment should be recommended for use on the NHS • Been diagnosed with cancer or cared for a family or close friend with cancer But are interested to learn more about cancer clinical trials and/or have a say in how they work and how their results are explained to your family, friends, and the public. If you are not sure which group to pick, everyone is a member of the public
A patient or carer*	For those who have cancer or have cared for someone with cancer and are interested to learn more about cancer clinical trials and/or have a say in how they work and how the results are explained to family, friends, and the public
A health professional	For those with a medical background and who have experience in treating or discussing cancer treatment options with patients
An industry partner^#^	For those who are employed by the pharmaceutical industry and work in the development of cancer treatments
A payer^#^	For those with experience in evaluating whether a new cancer treatment or intervention should be recommended for use in standard practice. For example, a member of a technology appraisal committee
A statistician or other data analyst	For those with a statistical or mathematical background with experience in analysing cancer clinical trial data. This also includes Health Economists and Data Scientists

^*^^#^ indicate groups which were combined to make a total of four stakeholder groups

The questionnaire was co-designed with five patients (including PW) over two sessions. This considered the patient information sheet (PIS), the content and format of the questions, and questionnaire structure on Online Surveys (JISC, Bristol, UK). The draft questionnaire was reviewed and tested by the project’s stakeholder advisory group (SAG). The SAG consists of at least one member of each stakeholder group. They meet and discuss key decisions about the study to ensure all stakeholder perspectives are considered.

In order to inform future methodological research, it was agreed that the questionnaire should include five core sections in addition to consent, demographics, request for optional further participation, and any final comments.

1. Question of interest: This section was included to inform what general question the future methodological research should investigate with particular attention on how to address participants stopping trial treatment and potentially commencing a subsequent treatment.
2. Information required: This section was included to consider what data could be collected on participants following discontinuation of trial treatment and how it should be obtained as existing methodology, e.g. the two-stage method, requires additional information at the point of switching therapy to be used in model development. If any data required for a method was not acceptable, then the method would not be investigated in future research.
3. Assumptions: This section was included to understand perceptions on statistical assumptions, as all methods require assumptions and if any assumptions were not acceptable the methods requiring them wouldn’t be considered in future work.
4. Answer: This section was included to be aware of how stakeholders like results presented to them so that any developed methodology could be presented in a way which would be understood.
5. Additional questions: This section was included to gather information on how to disseminate the work to statisticians through statistical software and understand whether work would be required to change practice for example in terms of data collection.

**Fig. 1:**
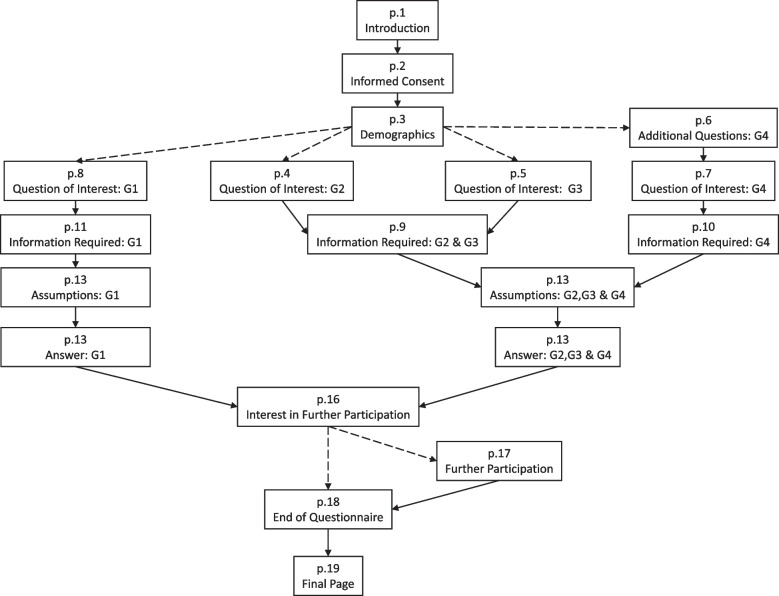
Questionnaire structure. Flow diagram of questionnaire structure. Solid lines indicate the next page, dashed lines indicate a custom route. The custom routes from p.3 were determined by the stakeholder group the respondent self-defined themselves as (G1: patients, carers and the public, G2: healthcare professionals, G3: payers and industry partners, G4: statisticians and other data analysts). The custom route from p.16 was determined by the respondent expressing an interest in further participation

The final questionnaire had 19 pages. However, this included optional pages and each of the four stakeholder groups had their own pathway through the questionnaire (Fig. 1) and its nine sections (Table 3). Therefore, the total number of possible questions for an individual was 83. However, many of the questions were optional opportunities for the respondent to explain their answers. In addition, aside from consent, there was only one mandatory question. This asked respondents to self-identify with a stakeholder group (Table 2). This was mandatory as it determined the respondent’s path through the questionnaire.

**Table 3:** Summary of the online questionnaire

Section	Summary	Pages^α^	Number of questions
Introduction and informed consent	An overview of the study with a frequently asked question section covering data protection and risks and benefits of taking part. The informed consent had 7 points to agree to	P2	7
Demographics	Twelve questions considering respondent demographics, baseline opinions on the topic and how they found the questionnaire	P3	12
Additional questions	Questions for statisticians and data analysts only, asking about current practices. This included what statistical software they use, what data has been collected during follow-up on their past trials and their previous analysis experience	P6	12
Question of interest	Ranking exercise of the question the respondent was most interested in with the option to explain and suggest additional questions. The pre-specified questions related to the five approaches to dealing with an intercurrent event (Table 1)	P4, P5, P7, P8	4
Information required	List of six data items, with definitions, which could be collected on patients for respondents to give their opinions on whether they should be collected. There was also the option for respondents to explain their answers, suggest additional data to be collected and score how data should be collected and recorded. The pre-specified data items included date and cause of death, anti-cancer treatment, patient and disease characteristics	P9, P10, P11	13*
Assumptions	List of seven statistical assumptions, with definitions, which could be applied for respondents to give their opinions on whether they were appropriate to be applied in all, some, or no scenarios. There was also the option for respondents to explain their answers after scoring each assumption. The pre-specified statistical assumptions included non-informative censoring, proportional hazards, common treatment effect, no unmeasured confounding, no time dependent covariates and no competing risks	P12, P13	15
Answer	List of six ways, with definitions, data can be presented for respondents to give their opinion on whether they are helpful. There was also the option for respondents to explain their answers after scoring each option. The pre-specified data presentation options included survival curves, median time, mean survival, hazard ratios, confidence intervals and p-values	P14, P15	13^¥^
Further participation	Option to hear about and consent to contact for additional participation in the focus groups and study updates	P16, P17	6
End of questionnaire	Opportunity for final comments	P18	1

^α^ The page number is as included on Fig. 1

^*^ For Healthcare professionals, an extra question was included for them to explain their answers. For the other stakeholder groups, this section only included 12 questions

^¥^ For non-patient/public groups, this section only included 9 questions. Following the advice of the co-design group, additional questions were included for the patient/public group to have the opportunity to explain their answers

The complete questionnaire is provided in Additional File 3 which includes all definitions and explanations

The participant information sheet (PIS) was the first page of the questionnaire and informed consent was obtained from respondents on the second page (Fig. 1, Table 3). The PIS included links to YouTube videos summarising the study and the purpose of the questionnaire [9] and provided a study email address for queries. The PIS also included statements on data sharing and incentives for respondents. The questionnaire software was GDPR compliant and there was no monetary incentive for taking part. At the end of the questionnaire, respondents could optionally consent to further participation. Respondents had the option to save their answers, complete it at a later date and move back through the questionnaire, but they could not view all their answers on a single page prior to submission.

The full questionnaire, including the PIS, is included in Additional File 3.

Ethical approval for the project was obtained from an NHS Research Ethics Committee (22/YH/0155).

#### Recruitment

The questionnaire was distributed electronically using targeted emails. The questionnaire was an open survey, i.e. registration was not required; participants self-selected and accessed the questionnaire through a link. The targeted emails were sent to all stakeholder groups and included charity groups, funding bodies and clinical trial units (Additional File 7: Supplementary Table 1). In addition to the targeted emails, social media was utilised to promote the study and associated questionnaire. At two-weekly intervals during recruitment, threads introducing different topics were shared on X (previously called Twitter). These included introductions to clinical trials (Additional File 7: Supplementary Fig. 2), statistics (Additional File 7: Supplementary Fig. 3), and survival analysis (Additional File 7: Supplementary Fig. 4). Two summary videos about the study were also made for X [10, 11]. Recruitment was for 8 weeks. A formal sample size calculation was not applicable for the questionnaire. However, a target of 30 respondents per stakeholder group was pre-specified. This equated to 120 respondents, which balanced the desire to collect a wide variety of views with conducting a qualitative analysis within the project timescales.

#### Analysis

The online survey software did not use cookies, check Internet Protocol (IP) addresses or provide a log file for analysis to identify potential duplicate responses. Therefore, any unidentifiable duplicates have been considered as separate responses. Furthermore, whilst the total number of visits to each page of the questionnaire was recorded, there was no way to determine the number of unique visits. Instead, the total number of completed (submitted) questionnaires was compared to the total number of respondents who provided informed consent.

At the end of recruitment, the questionnaire responses were downloaded and read into SAS 9.4 (SAS Institute, Cary, NC). Analysis was descriptive. Themes were identified from the free-text fields using a conventional content analysis approach [12]. Prior to the analysis, consensus guidelines, based on the RAND/UCLA method, were agreed between research team (K-LR, DM, JV-R, IRW and DAC) (Table 4). Questionnaires with missing responses were included in the analysis, but missing answers did not feed into the consensus guidelines. Statistical correction was not applied to adjust for a non-representative sample.

**Table 4:** Questionnaire decision guidelines

Guidelines	Question type	Guidelines
Question decision guideline	Ranked (1–4)	Include—median = 1 Uncertain—median = 2–3 or disagreement Exclude—median = 4 Disagreement: ≥ 1/3 in 1 and ≥ 1/3 in 4
Data decision guideline	Likert (1–5)	Include—median = 1–2 Uncertain—median = 3 or disagreement Exclude—median = 4–5 Disagreement: ≥ 1/3 in 1 and ≥ 1/3 in 5
Choice guideline	Multiple choice (choose 1/2)	The one with the highest percentage of respondents would be considered for further discussion
Assumptions guideline	Likert (1–3 + unsure)	Include—median = 1 Uncertain—median = 2 or disagreement Exclude—median = 3 Disagreement: ≥ 1/3 in 1 and ≥ 1/3 in 3 OR 50% in unsure
Answer guideline	Likert (1–5)	Include—median = 1–2 Uncertain—median = 3 or disagreement Exclude—median = 4–5 Disagreement: ≥ 1/3 in 1 and ≥ 1/3 in 5

### Focus group

#### Design

Following the analysis of the online questionnaire, the results were discussed and interpreted within the research team and a proposal was presented to the SAG (including PW) as to which aspects needed further discussion (Additional File 4). Once agreed, the focus group materials (a pre-focus group information pack and a post-focus group questionnaire) were finalised in collaboration with the research team and SAG. The pre-focus group information pack, included an overview of the questionnaire results, which aspects reached consensus from the pre-defined rules, and a plan of what would be asked and discussed at the focus group (Additional File 5). The post-focus group questionnaire formalised the recommendations made within the focus group by asking attendees to re-rate the points discussed during the focus group and allowed for feedback outside the wider group (Additional File 6). Ethical approval for focus group materials was obtained via a substantial amendment.

Focus group invitees were randomly selected from the questionnaire respondents who consented to be contacted for further participation. The focus group aimed to have 12 attendees equally balanced by stakeholder group and sex. Therefore, consenting respondents were stratified by stakeholder group and gender. Invitees were then randomly selected from each stratum. The random sampling was conducted using the procedure surveyselect in SAS 9.4. Any questionnaire respondents who declined were replaced by resampling from the relevant stratum.

#### Format

The focus group was a hybrid meeting with K-LR chairing (a female PhD student with an MSc in Statistics). Prior to the focus group, K-LR had attended focus groups with different stakeholder groups and discussed with colleagues their experience of leading a focus group. PW facilitated the focus group in person with K-LR. Attendees joined online using Microsoft Teams (Microsoft, Redmond, WA, USA). Breakout rooms were utilised to allow for within and across stakeholder discussion. K-LR did not input into the discussion to reduce the possibility of bias. During the focus group, attendees could write notes using Padlet (San Francisco, CA, USA; Singapore). The focus group was scheduled for 3 h. As the attendees had completed the questionnaire, they knew about the researcher (K-LR), and the reasons for the research from the PIS. Following the focus group, attendees were asked to complete the post-focus group questionnaire online.

#### Analysis

The focus group recording and notes were summarised. The post-focus group questionnaire was analysed descriptively by K-LR where free text was considered in full. The meeting summaries and answers to the questionnaire were used to finalise consensus, where possible, and provide guidance for the next step of the project.

## Results

### Online questionnaire

Questionnaire recruitment took place between 3rd August 2022 and 30th September 2022. There were 1808 visits to the introduction page, 314 gave informed consent and 103 stakeholders completed the questionnaire, resulting in a completion rate of 33% (patient, carer, or member of the public: 45/103 (44%), health professional: 22/103 (21%), payer or industry partner: 6/103 (6%), statistician or other data analyst: 30/103 (29%)).

Results are presented as per the questionnaire sections in Table 3.

#### Demographics

The respondents predominantly found the questionnaire from a mailing list 35/103 (34%), had a median age of 52, were mostly female 63/103 (61%), from the UK and NI 84/103 (81%) and had some prior experience of clinical trials 68/103 (66%). When asked about their opinion on the topic, 81/103 respondents (78.6%) thought that subsequent treatments should be taken into consideration when analysing OS (Table 5).

**Table 5:** Summary of questionnaire respondents

	Patient, carer or member of the public (*n* = 45)	Healthcare professional (*n* = 22)	Payer or industry partner (*n* = 6)	Statistician or other data analyst (*n* = 30)	Total (*n* = 103)
How old are you?					
Median (IQR)	60 (54,66)	48 (38,55)	43 (37,48)	40 (30,48)	52 (40,60)
Missing	1	0	0	3	4
What is your ethnic group?					
English/Welsh/Scottish/Northern Irish/British	41 (91.1%)	17 (77.3%)	6 (100.0%)	20 (66.7%)	84 (81.6%)
Irish	0 (0.0%)	2 (9.1%)	0 (0.0%)	2 (6.7%)	4 (3.9%)
African	0 (0.0%)	1 (4.5%)	0 (0.0%)	0 (0.0%)	1 (1.0%)
Caribbean	1 (2.2%)	0 (0.0%)	0 (0.0%)	0 (0.0%)	1 (1.0%)
Other, please describe	3 (6.7%)	1 (4.5%)	0 (0.0%)	2 (6.7%)	6 (5.8%)
Prefer not to say	0 (0.0%)	1 (4.5%)	0 (0.0%)	3 (10.0%)	4 (3.9%)
Missing	0 (0.0%)	0 (0.0%)	0 (0.0%)	3 (10.0%)	3 (2.9%)
What sex are you?					
Male	10 (22.2%)	9 (40.9%)	2 (33.3%)	12 (40.0%)	33 (32.0%)
Female	33 (73.3%)	12 (54.5%)	4 (66.7%)	14 (46.7%)	63 (61.2%)
Prefer not to say	1 (2.2%)	0 (0.0%)	0 (0.0%)	1 (3.3%)	2 (1.9%)
Missing	1 (2.2%)	1 (4.5%)	0 (0.0%)	3 (10.0%)	5 (4.9%)
Where in the UK are you based?					
Scotland	4 (8.9%)	1 (4.5%)	0 (0.0%)	2 (6.7%)	7 (6.8%)
Northern Ireland	1 (2.2%)	0 (0.0%)	0 (0.0%)	2 (6.7%)	3 (2.9%)
North West England	4 (8.9%)	1 (4.5%)	1 (16.7%)	2 (6.7%)	8 (7.8%)
North East England	1 (2.2%)	1 (4.5%)	0 (0.0%)	0 (0.0%)	2 (1.9%)
Yorkshire and The Humber	18 (40.0%)	12 (54.5%)	2 (33.3%)	10 (33.3%)	42 (40.8%)
East Midlands	4 (8.9%)	2 (9.1%)	0 (0.0%)	0 (0.0%)	6 (5.8%)
West Midlands	1 (2.2%)	0 (0.0%)	0 (0.0%)	3 (10.0%)	4 (3.9%)
London	1 (2.2%)	3 (13.6%)	3 (50.0%)	5 (16.7%)	12 (11.7%)
South East England	6 (13.3%)	0 (0.0%)	0 (0.0%)	2 (6.7%)	8 (7.8%)
South West England	3 (6.7%)	1 (4.5%)	0 (0.0%)	1 (3.3%)	5 (4.9%)
Other	2 (4.4%)	0 (0.0%)	0 (0.0%)	2 (6.7%)	4 (3.9%)
Prefer not to say	0 (0.0%)	1 (4.5%)	0 (0.0%)	0 (0.0%)	1 (1.0%)
Missing	0 (0.0%)	0 (0.0%)	0 (0.0%)	1 (3.3%)	1 (1.0%)
Where did you find out about this questionnaire—mailing list					
Selected	10 (22.2%)	11 (50.0%)	2 (33.3%)	12 (40.0%)	35 (34.0%)
Where did you find out about this questionnaire—social media					
Selected	21 (46.7%)	3 (13.6%)	0 (0.0%)	0 (0.0%)	24 (23.3%)
Where did you find out about this questionnaire—word of mouth					
Selected	4 (8.9%)	0 (0.0%)	1 (16.7%)	6 (20.0%)	11 (10.7%)
Where did you find out about this questionnaire—other					
Selected	11 (24.4%)	8 (36.4%)	3 (50.0%)	11 (36.7%)	33 (32.0%)
Are you?					
A member of the public	13 (28.9%)	0 (0.0%)	0 (0.0%)	0 (0.0%)	13 (12.6%)
A patient or carer	32 (71.1%)	0 (0.0%)	0 (0.0%)	0 (0.0%)	32 (31.1%)
A health professional	0 (0.0%)	22 (100.0%)	0 (0.0%)	0 (0.0%)	22 (21.4%)
An industry partner	0 (0.0%)	0 (0.0%)	2 (33.3%)	0 (0.0%)	2 (1.9%)
A payer	0 (0.0%)	0 (0.0%)	4 (66.7%)	0 (0.0%)	4 (3.9%)
A statistician or other data analyst	0 (0.0%)	0 (0.0%)	0 (0.0%)	30 (100.0%)	30 (29.1%)
Have you had any prior experience of clinical trials?					
Yes	20 (44.4%)	16 (72.7%)	4 (66.7%)	28 (93.3%)	68 (66.0%)
No	25 (55.6%)	6 (27.3%)	2 (33.3%)	2 (6.7%)	35 (34.0%)
Do you think it is important to consider the effect that treatment given after trial treatment has on overall survival?					
Yes	40 (88.9%)	16 (72.7%)	6 (100.0%)	19 (63.3%)	81 (78.6%)
No	0 (0.0%)	0 (0.0%)	0 (0.0%)	1 (3.3%)	1 (1.0%)
Depends	3 (6.7%)	4 (18.2%)	0 (0.0%)	8 (26.7%)	15 (14.6%)
Unsure	2 (4.4%)	2 (9.1%)	0 (0.0%)	2 (6.7%)	6 (5.8%)

#### Additional questions

In total, 30 respondents identified themselves as a statistician or other data analyst and answered the additional questions about their previous experiences analysing OS.

The majority of the 30 respondents used the statistical programming languages STATA or SAS (22/30, 73.3%) to conduct statistical analysis. Seven (23.3%) used the language R and one respondent did not complete the question.

**Fig. 2:**
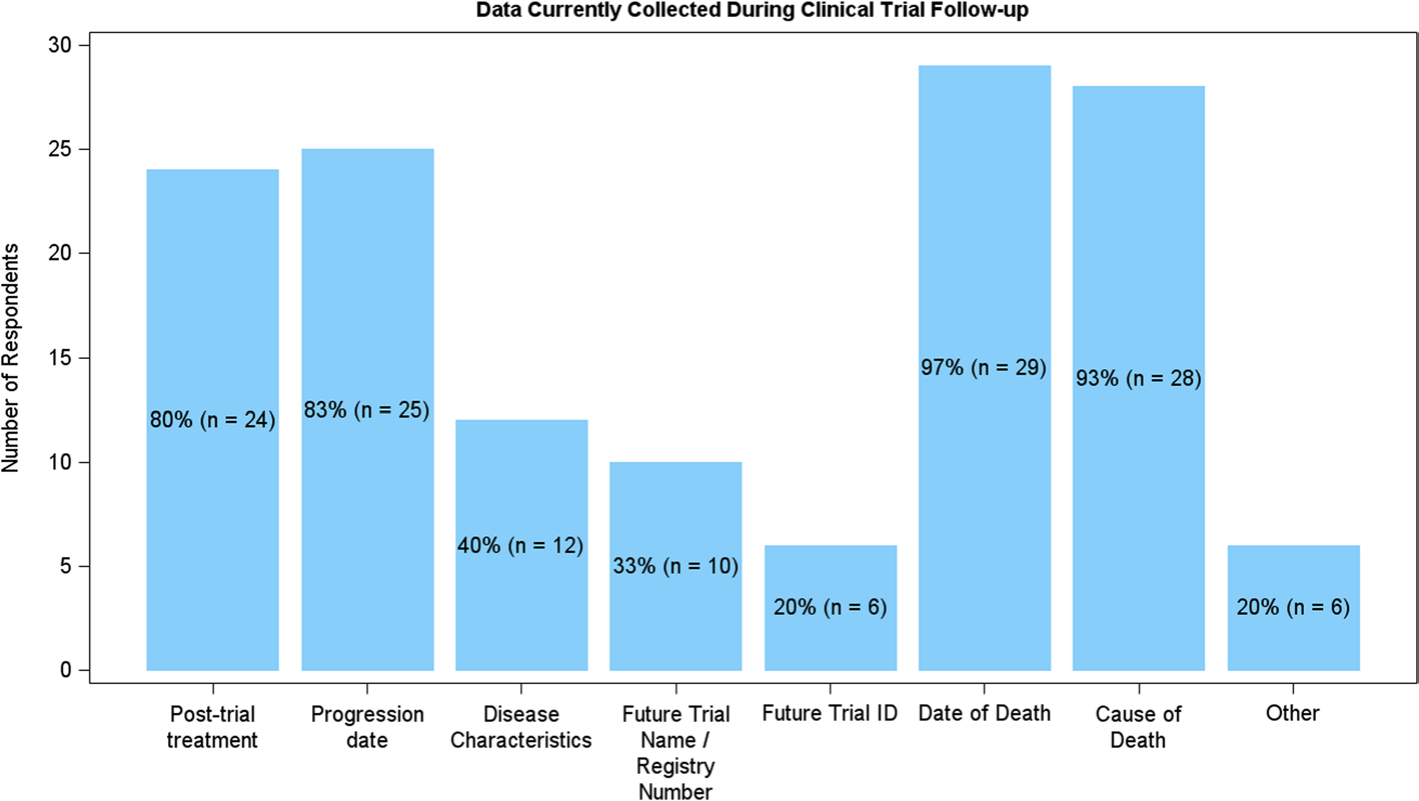
Bar chart showing the number of statisticians or other data analysts who collect information during trial follow-up

In terms of data collection, post-trial treatment, progression date, and date and cause of death were reported as being collected during trial follow-up by a very high proportion of respondents (Fig. 2). However, disease characteristics and participation in future trials along with trial identification number were not selected as often. When asked for other data which is collected, respondents answered with quality of life (QoL) and other safety information. It was also noted that “This is really hard to answer as it really depends on the nature of the index trial/length of follow-up” and “could be any or all of these—there isn’t one standard” highlighting that there is not a common approach to data collection across trials.

**Fig. 3:**
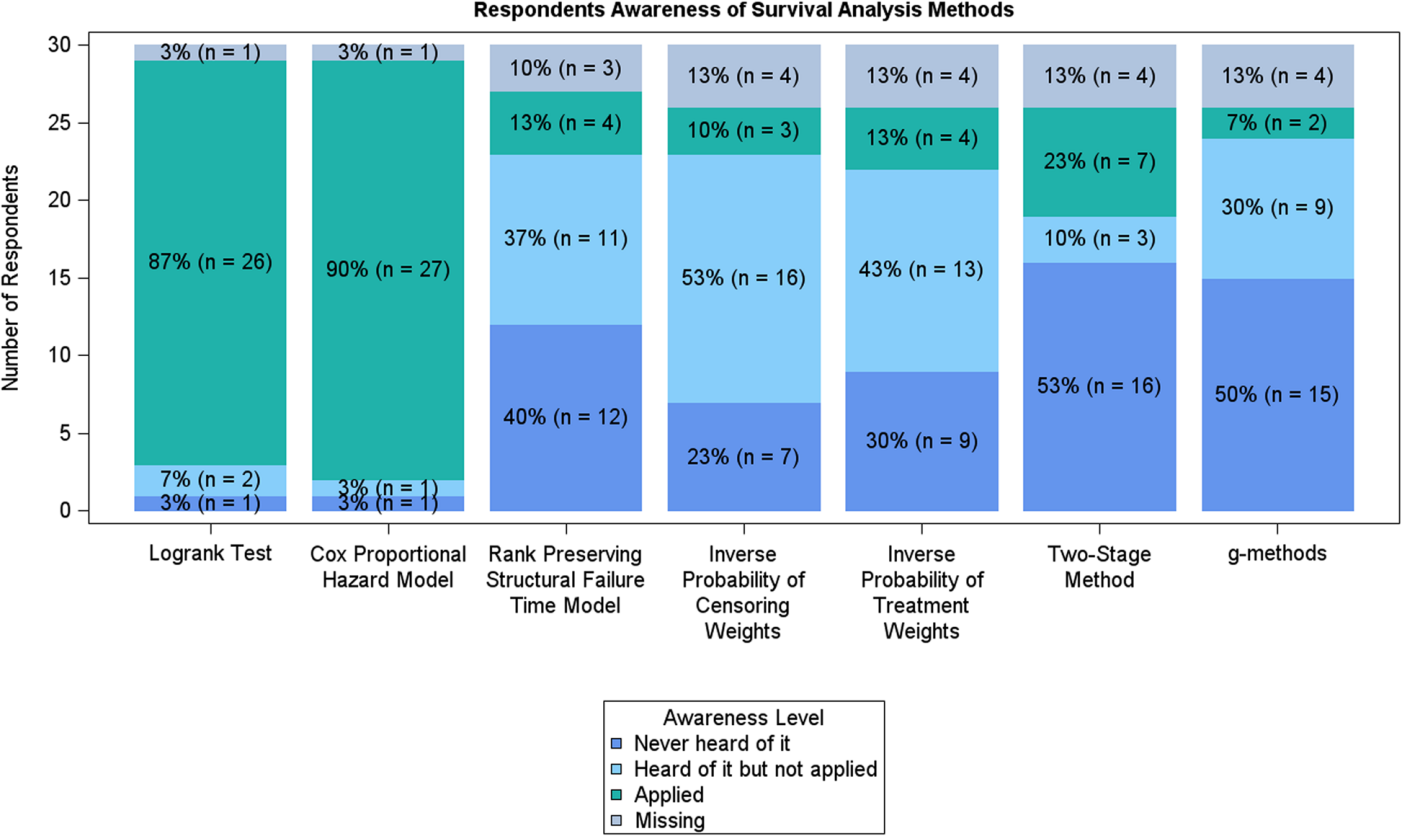
Bar chart showing statisticians and other data analysts’ awareness of methodology. The log-rank test and the Cox proportional hazard model are statistical methods which are widely used in the literature. The remaining methods are less widely used in the literature

On consideration of the statistical methods used to analyse OS, often referred to as survival analysis, most respondents, as expected, had applied the log-rank test and the Cox proportional hazards model which were the most widely used survival analysis methods used in a literature review on the topic [5]. Respondents had heard of, but not applied, IPCW (inverse proportional censoring weighting) and IPTW (inverse proportional treatment weighting) and had not heard of the RPSFTM (rank preserving structural failure time model), the two-stage method or g-methods (Fig. 3). This is not surprising given that these methods are more complex and applied in specific scenarios like treatment switching. In fact, only (5/30) 16.7% of respondents had adjusted OS analysis to account for subsequent lines, with the two-stage method, landmark analysis and RPSFTM being specifically mentioned. Eight respondents took up the opportunity to elaborate on their answers. One respondent commented “Also used semiparametric methods such as spline-based APC models, accelerated failure time, proportional odds and frailty models”. These again are considered more advanced survival analysis methods and were rarely reported as being implemented in the literature review.

#### Question of interest

**Fig. 4:**
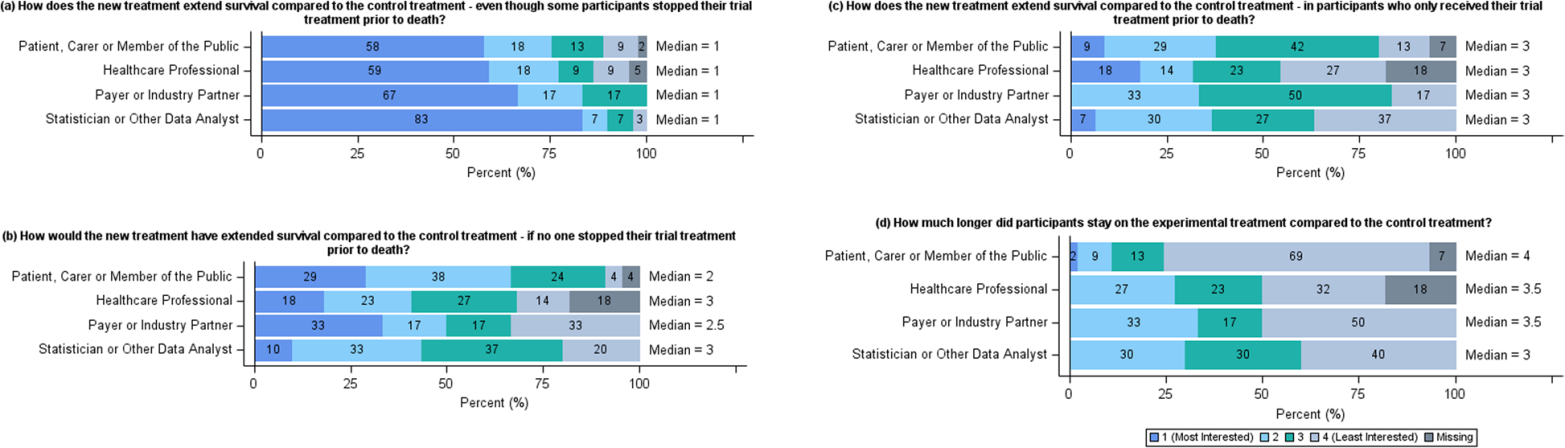
Summary of question of interest results. Horizontal stacked bar charts showing the responses to the question “Please read the five questions below and rank them in order of what would be the most important for you to know the answer to (1) to the least important (4) if you or a friend were being treated for cancer. Please remember this is asking what you think, there are no right or wrong answers.” from the online questionnaire separated by stakeholder group

Following the pre-specified guidelines (Table 4), consensus was reached that the question of interest to each stakeholder group was “How does the new treatment extend survival compared to the control treatment—even though some participants stopped their trial treatment prior to death?” (Fig. 4). This relates to the treatment policy method of addressing an intercurrent event (Table 1). The main themes from the additional questions posed from respondents were, details on the subsequent treatments, consequences of being in the trial, other effects of the trial treatment and how patient characteristics influenced the treatment effect. At the SAG, it was agreed that the decision for the research to consider the first question should be discussed at the focus group.

#### Information required

**Fig. 5:**
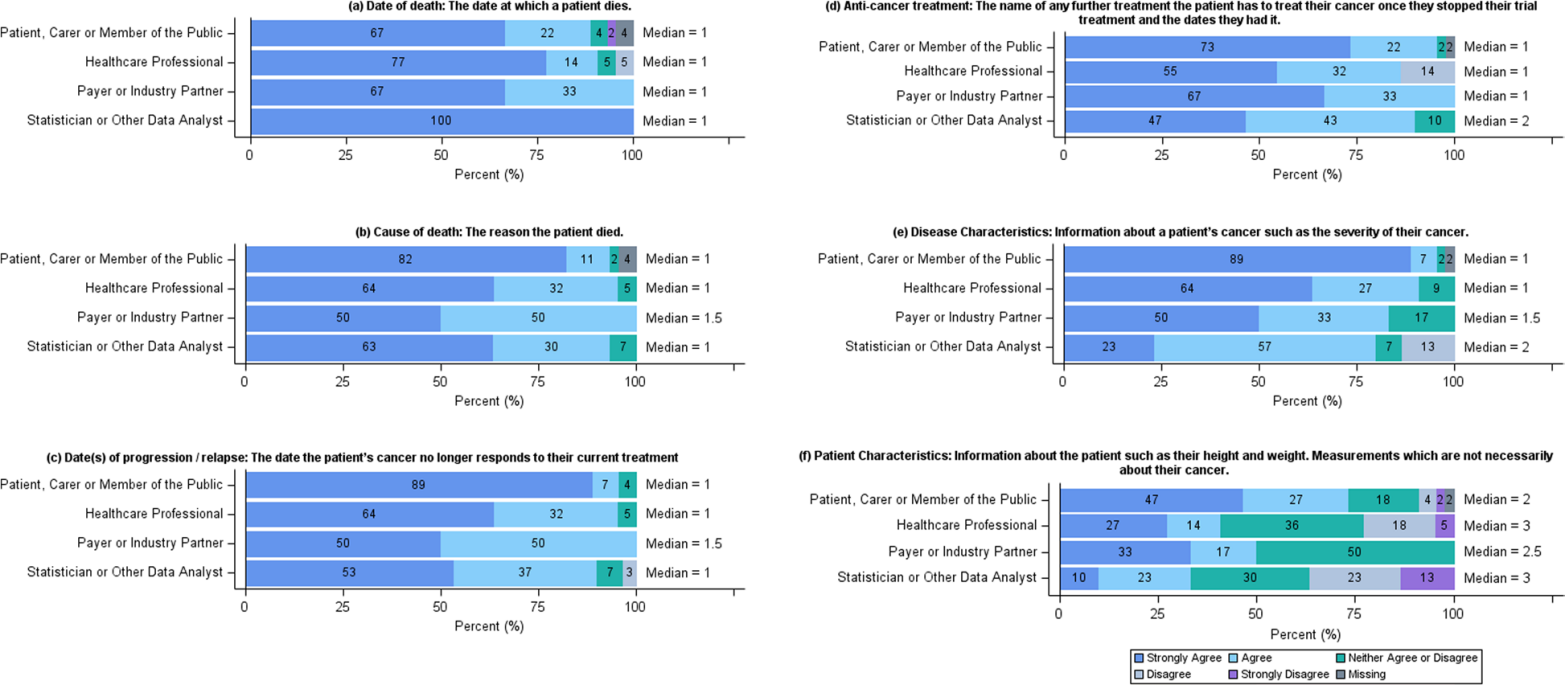
Summary of data collection responses. Horizontal stacked bar charts showing the responses to the question “Please consider each piece of information listed below and decide whether you agree it is practical and appropriate to collect this information about clinical trial participants, after they have stopped receiving their trial treatment.” from the online questionnaire separated by stakeholder group

Consensus was reached that date of death, cause of death, date(s) of progression/relapse, subsequent anti-cancer treatment and disease characteristics could be collected on trial participants once they have stopped their trial treatment (Fig. 5). However, there was disagreement around patient characteristics. Therefore, the collection of patient characteristics along with the collection of co-morbidities, concurrent treatment, QoL/psychological data, toxicity and participation in future trials which were suggested by questionnaire respondents, were agreed to be discussed at the focus group.

**Fig. 6:**
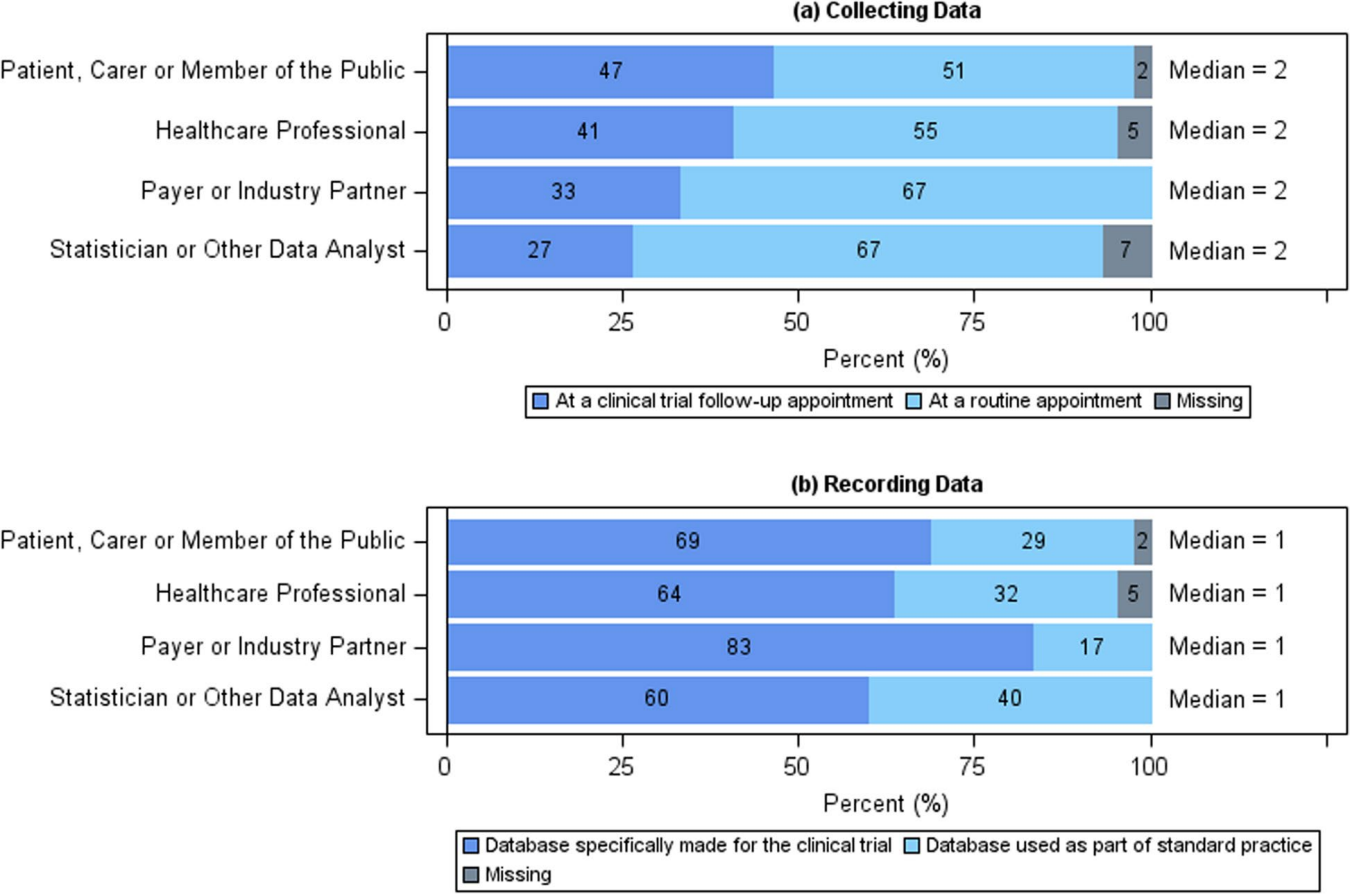
Summary of collecting data and recording information results. **a** Horizontal stacked bar chart showing the responses to the question “There are two different ways this information can be collected from patients who have stopped their trial treatment. Which do you think is the most appropriate?” from the online questionnaire separated by stakeholder group. **b** Horizontal stacked bar chart showing the responses to the question “There are also two different ways this information can be recorded and returned to the trial researchers. Which do you think is the most appropriate?” from the online questionnaire separated by stakeholder group

When asked how data should be collected and recorded on participants once they have stopped their trial treatment, the majority of stakeholders thought that data should be collected at a routine appointment (Fig. 6). However, reasons were provided for both routine and trial follow-up appointments. The main reasons provided in favour of a trial follow-up appointment were as follows: trial focus, the respondents’ explanation mentions that a trial follow-up appointment is conducted by the research team who will keep focus on trial and ensure data collection is completed; phone preference, the respondents’ explanation mentions that a phone appointment is preferable to a face-to-face appointment to reduce burden on patient; and personal experience, the respondents’ explanation includes reference to their own opinion and experience of trials. On the other hand, the main reasons provided in favour of a routine appointment were as follows: patient burden, the respondents’ explanation includes reducing the burden and requirement on patients to attend hospital visits in order to improve compliance; context, the respondents’ explanation notes that the regularity of follow-up appointments would influence their decision on a trial-by-trial basis; and logistics, the respondents’ explanation includes a consideration of how it would work in practice. Similarly, whilst most stakeholders felt that data should be recorded on a database which was made specifically for the trial (Fig. 6), reasons were provided for both options. The main reasons provided in favour of a trial database were as follows: standardised data collection, the reasoning includes that the required data is collected reliably and consistently in a controlled way to allow for it to be as accurate as possible to ensure a robust analysis and routine data access, the reasoning includes a comment around the barriers to accessing data from routine data sources. Alternatively, the main reasons provided in favour of a standard database were as follows: site burden, the respondents’ explanation includes a comment about reducing the pressure on site staff to input data; duplication, the respondents’ explanation includes a comment around reducing duplication across databases; and data sharing, the respondents’ explanation favours data sharing between general practice and researchers. Following discussion with the SAG, it was agreed that the collection and recording of data once trial treatment has stopped should be discussed with the focus group and extended to consider what is currently possible, and what would be ideal if the resources were available.

#### Assumptions and answer

In terms of statistical assumptions, the majority were scored as being appropriate in “Some Scenarios” (Additional File 7: Supplementary Fig. 5). The results were discussed with the SAG and decided not to be taken to the focus group. Similarly, as most ways to present the information were scored as “Very Helpful” or “Helpful” (Additional File 7: Supplementary Fig. 6), it was agreed that data presentation would not be discussed further. Instead, any future work will be presented to stakeholders for review and updated accordingly.

### Summary

To summarise, following the online questionnaire there were three discussion points for the focus group:

1. The decision to consider the treatment policy approach of addressing the intercurrent event with the question “How does the new treatment extend survival compared to the control treatment—even though some participants stopped their trial treatment prior to death?”
2. The data items that are collected once trial treatment has finished.
3. How data is collected and recorded once trial treatment has finished.

These three discussion points were included with the full results of the questionnaire in a pre-meeting discussion pack (Additional File 5). The first point was presented with the researcher’s opinion as consensus had been reached within the initial questionnaire based on the pre-defined criteria (Table 4). The remaining were presented as open-ended questions. The schedule of the focus group was also included in the pre-meeting pack and included the explicit questions the focus group would be discussing (Additional File 5, page 12).

### Focus group

Just under half of respondents (50/103, 48.5%) were interested in learning about further participation in the study. This resulted in 33 (32%) of respondents consenting to being contacted for attendance at discussions with other respondents in a different stakeholder group. The 33 respondents predominantly identified as a patient, carer, or member of the public (20/33, 60.6%). The other respondents identified as healthcare professional (4/33, 12.1%), payer or industry partner (2/33, 6.1%) and statistician or other data analyst (10/33, 30.3%). In total, 19 respondents were invited (Additional File 7: Supplementary Table 2). Of the eight who declined, all were not available on the day of the focus group. One of the respondents who declined sent a colleague in their place. For this attendee, consent was obtained via email as they had not completed the original questionnaire. In total, the focus group had seven attendees, balanced for stakeholder group but not for sex (Additional File 7: Supplementary Table 2).

#### Discussion 1: the question of interest

The focus group considered the researcher’s decision to consider the treatment policy approach by focusing on the question “How does the new treatment extend survival compared to the control treatment—even though some participants stopped their trial treatment prior to death?”. All stakeholder groups agreed that this was a logical approach and that it reflected real life. A patient member commented “When you were talking about reality, that’s a word that resonated. I suppose being slightly suspicious in general life as a patient you would wonder what had happened to the other people, the special people who we weren’t being reported back on.” However, patients did comment that QoL would still be of interest but accepted that was not the focus of the study.

These thoughts in terms of logic and interest in QoL were re-iterated by the five attendees (three statisticians and two patients) who completed the post-focus group questionnaire. The three statisticians responded: “I think this is a choice which makes sense both to clinical experts and to patients. It [does] not preclude pre-specified analyses of narrower questions.”; “I think[this]is the best focus as it focuses on the pragmatic realistic question that is of most interest to patients and clinicians”; and “I think this is the most common question of primary interest, so it makes sense to focus on this. Although context might dictate that considering the other options is more or less relevant, I would imagine this question would have greatest interest.” The two patients responded: “I’m happy with this as it reflects the real world not some artificial scenario” and “Quality of life should be taken into account. Survival might be longer but at what cost to quality of life?”.

#### Discussion 2: data items

The focus group considered the collection of: patient characteristics, whether participants received subsequent anti-cancer treatment as part of a clinical trial, QoL/psychological data and toxicity data, once the trial participant has stopped taking their trial treatment. All stakeholder groups agreed that collecting data from trial participants is acceptable when: there is a clear rationale, patients have been informed during the consent process; and patients are involved in the decision to collect the data. In the post-focus group questionnaire, all items reached consensus to be included overall (median 1–2, Table 4) and the majority reached consensus within each stakeholder group (Additional File 7: Supplementary Fig. 7). Only patient characteristics did not meet the pre-defined consensus guidelines within the statistician or other data analyst group (Additional File 7: Supplementary Fig. 7a). This reiterates the point made during the focus group discussion from this group that there needs to be a clear rationale for data collection.

#### Discussion 3: data collection and recording

When thinking about how data is collected, perhaps months or years after the participant has stopped taking their trial treatment, the perspectives from each stakeholder group focused on reducing participant and site burden and encouraging the use of remote data capture over the telephone where possible. In the focus group, all groups agreed that routinely collected data would be ideal, but understood the statisticians concerns around data access requirements changing during the trial and that the data needed for the trial may not be collected routinely potentially resulting in research waste. A statistician summarised the issue as “That’s what it comes down to, the whole thing is a waste if we don’t get the right data”.

In the post-focus group questionnaire, consensus was reached within each stakeholder group for collecting information in the current research world with patients, carers or members of the public preferring a trial follow-up appointment and statisticians or other data analysts preferring a routine follow-up appointment. In an ideal research world, both stakeholder groups preferred a trial follow-up appointment to collect data (Additional File 7: Supplementary Fig. 8). This reflected some of the discussions within each stakeholder group during the focus group. The patient, carer and member of public group raised the context around the disease. They noted that if routine appointments do not happen as part of standard of care, then a trial appointment was necessary. In addition, they commented that if the information is only needed for trial purposes rather than routine care that the trial team should collect it to reduce the burden on sites. The statisticians and other data analysts also commented that it would make sense to use the routine appointments, but that it depended on the standard of care visit schedule and whether it reflects the follow-up schedule required for the trial outcomes.

In terms of recording information, consensus was reached across the stakeholder groups for recording information in the current research world, with all groups preferring a database specifically made for the trial (Additional File 7: Supplementary Fig. 9a). However, in the ideal research world, whilst statisticians or other data analysts preferred recording information using a database which is completed normally as part of standard practice, patients, carers or members of the public were evenly split. However, if considered together, the consensus is to use a database completed normally as part of standard practice. This reiterates the discussion in the focus group, where patients did not mind where the data was stored if it was accessible and minimised patient and clinician burden, and statisticians wanted to use routine data if it collected the correct information and was accessible.

### Summary

Following the summary of the discussions and analysis of the post-focus group meeting questionnaire, it was concluded that sufficient information and perspectives had been obtained to inform future methodology work but that wider conversations on data storage and recording would be needed in the future. Therefore, no further rounds (pre-focus group questionnaire, focus group and post-focus group questionnaire) were conducted.

## Discussion

The overarching aim of this qualitative study was to obtain consensus from stakeholder groups, to guide the development of a methodological solution to analyse OS when trial participants receive subsequent treatment lines during trial follow-up, which is an intercurrent event. This was achieved as consensus was reached on the majority of topics, and the future direction of the project was agreed at the focus group. As no additional topics, within the scope of the project, were raised from the questionnaire or the focus group, data saturation was concluded to have been reached.

Following both parts of the study, it was agreed that future work should consider the question: “How does the new treatment extend survival compared to the control treatment—even though some participants stopped their trial treatment prior to death?” Important caveats were that the question does not: Assume no one stopped their trial treatment prior to death; consider only those who only received their trial treatment prior to death or shorten overall survival to be the time on treatment. Considering the estimand framework, this question relates to the treatment policy method of dealing with intercurrent events. There are two possible approaches to this question. The first is to ignore subsequent treatment lines in the estimation of the trial treatment effect for OS. This is already done in practice [5] and is the definition of the treatment policy approach. As most stakeholders thought that the potential effect of subsequent treatment lines should be considered during the analysis of OS, an alternative approach will be explored in the next step of this work utilising both a simulation study and real data. The next step will consider the hypothetical scenario assuming all participants, where appropriate, went onto receive the same second-line of therapy. This will aim to provide a sensitivity analysis for the treatment policy approach, to allow researchers to begin to quantify the impact of subsequent treatments. This is thought to be predominantly of interest in the scenario where the treatment landscape has changed during trial-follow-up and there are more effective options available for patients. This decision could be criticised in that stakeholders have not been explicitly asked to comment on this direction. However, from the results of the general questionnaire, all stakeholders agreed that the subsequent treatment lines should be considered, and this question is considered a natural next step by the investigators. It is also unknown whether an answer to the question can be obtained. Following an investigation via a simulation study and application to real data, stakeholders will be invited to comment again and future directions, including alternative estimands, agreed collaboratively. The aim of this stakeholder consultation was to understand current opinions, ensure that this research area was of interest to all and guide the future research which have been met.

The future work will be guided using the results from the qualitative study presented here. Only data which was scored “Appropriate” with a clear rationale to collect will be simulated, and different scenarios considering different levels and routes of data collection during post-trial treatment will be used to compare how the different methods perform in different contexts before applying them existing trial data. The consideration of different contexts is also important when applying the methods themselves and acknowledging the statistical assumptions they use. All stakeholders agreed that all assumptions were applicable in “Some Scenarios” and it was noted that one method or approach may not fit all trial designs. The use of alternative data generating mechanisms will help to determine which scenarios the assumptions are applicable in, to help future researchers decide which method they should use when considering the context of their trial.

Once complete, the best way to describe and present the future work to the different stakeholder groups will be discussed and agreed with the SAG and consenting questionnaire respondents. Using the questionnaire results, a visual representation, a summary statistic such as a median and a hazard ratio which will be supported by at least a confidence interval will aim to be presented as a starting point for discussion.

Following the responses from the statisticians and other data analysts, to support the impact of any developed methodology, it will be first created in Stata (18, StataCorp LLC, College Station, TX) and then translated into SAS (SAS Institute, Cary, NC) and R (R Core Team, Vienna, Austria), where appropriate. In addition, as many of the statisticians and other data analysts had not heard of the more advanced methods currently available to tackle the specific event of treatment-switching (Fig. 3), a variety of different methods of dissemination will be utilised. This will be planned with the SAG and will include pre-conference workshops, conference abstracts, journal articles and social media to improve uptake of the work. It is important to note that the questionnaire and focus group also identified QoL as a key area for future research. Whilst QoL is outside the scope of this project, patients, carers, and the public were interested to know how the long-term QoL compared to long-term survival so that the information could be used in combination for patients with differing priorities to make an informed decision about their treatment. Another avenue for future research, which is outside the scope of this project, is trial design methodology. This work is focusing on the analysis methods. However, trialists may want to consider how trials could be designed to address the problem of subsequent treatment lines upfront. An example of an area to consider could be the implementation of trials with a SMART (Sequential, Multiple Assignment, Randomized Trial) design [13]. Furthermore, the discussions around how information is collected and recorded during trial follow-up is an interesting are to pursue further. However, the current movement in this area to store health systems data in secure/trusted research environments needs to be completed first before data availability and access can be considered. At present, the only approach to consistently collect this data is through trial specific case report forms which requires consideration at trial outset.

In terms of the general process, the study follows a modified RAND/UCLA appropriateness method (RAM) which may be seen as a limitation as it deviated from the traditional RAM approach. The traditional RAM invites a small group of experts to rate the a list of items before and after a meeting to discuss their views, where the items to be rated are identified through a review of the literature [6]. In the implemented RAM the items to be rated initially were identified by K-LR in discussion with the co-design group as areas which needed consensus to guide the development of statistical methodology. The items which were then discussed and re-rated in the focus group were determined by those which had not reached consensus following analysis of the questionnaire using traditional RAM rules (Table 4) and ratification by the SAG. This modification was chosen to obtain a wider range of expert views to the traditional RAM. However, not every stakeholder group was well represented. Only six payers and industry partners completed the questionnaire. This is not seen as a major limitation as review panels include representatives from each stakeholder group. In addition, there was not a pre-focus group rating as all attendees had intended to be questionnaire respondents, so their pre-discussion views should have already been recorded. Another deviation from the traditional RAND was that the experts self-selected themselves to be invited to take part in the focus group in the initial questionnaire. This approach was chosen to reduce bias by not only those in the immediate research teams’ network being invited to take part.

Another limitation is the terminology used in the questionnaire. As it was directed at multiple stakeholder groups, the attempt to balance the terminology in the questionnaire made it too complex for some patients, but too simplistic for some statisticians. On reflection, this could have been resolved by having separate questionnaire for every stakeholder group rather than separate questionnaire pathways. In addition, at the suggestion of the co-design group, the ethnic groups used within the questionnaire were taken from the categorizations used by the Office of National Statistics [14]. However, it was commented that “The ethnic groups are really odd”. Therefore, the questionnaire may have benefited from further testing. Nonetheless even with these changes or additional testing of the questionnaire, it is still likely that not every respondent would have been happy with every aspect of the questionnaire.

Considering technical limitations, hosting the questionnaire online meant that it was subject to the provider restrictions. This was a particular limitation for the format and the fact Cookies were not collected to stop duplicate responses. Given the length of the questionnaire it is unlikely anyone completed it twice and the alternative to require respondents to log in would have added an unnecessary recruitment barrier.

In terms of the focus group, only seven out of the intended 12 attended on the day despite 19 being invited. It may be that in the time between the closure of the questionnaire (September 2022) and the focus group (March 2023) invited respondents may have changed emails or forgotten about completing the survey. A few attendees also dropped out in the days prior to the focus group, which made it difficult to invite another in their place. In the future, we would recommend inviting more than the required sample size to account for last minute dropouts. This may have also led to the imbalance of stakeholders within the focus group. Whilst every stakeholder group was represented, there were more statisticians (three) and patients (two) than payers and industry partners (one) and healthcare professionals (one). An alternative format would have been to offer multiple dates and times for focus groups so that more people could attend, but this has its implications in terms of time and cost. However, it is thought that hosting it online using Microsoft Teams was a strength, as attendees did not have to travel to input into the discussions.

The main strength of this study is the use of patient and public involvement (PPI) in conjunction with participatory research tools to determine what should be considered during the development of statistical methodology. This approach is not novel but is rare with only a few examples existing in the literature, such as the work by Flight using qualitative methods to identify stakeholder views on the use of health economics in the design and analysis of trials [15]. Given the impact methodological decisions can have on the outcome, it seems counterintuitive to not include the patients and the public perspective and there is a movement at present to improve their general involvement in the numerical aspects of trials [16, 17]. From this study, both the patient members on the co-design group and focus group attendees were enthusiastic to be included with an attendee commenting that they had “enjoyed the session” and another one noting that at the beginning of the focus group they wondered if they would be able to understand it, but they did. This feedback, along with the fact that more than a third of questionnaire respondents were patients and the public demonstrates that the patient and public stakeholder group are interested in being included in methodological and numerical discussions, and that it is down to the researcher to consider, with PPI representatives, which aspects are appropriate and make them accessible.

## Conclusion

This work is an example of successfully implementing qualitative research in the process of developing statistical methods. By seeking consensus from stakeholder groups, a range of perspectives have been formally obtained that can be used as an evidence base to guide the development of a methodological solution to analyse OS when trial participants receive subsequent treatment lines during trial follow-up. If these findings are applied, we can be confident that any developed methodology is relevant and aligns to the interests of each stakeholder group.

## Supplementary Information

Additional File 1: CHERRIEs Checklist.

Additional File 2: COREQ Checklist.

Additional File 3: Patient Information Sheet (PIS) and Online Questionnaire.

Additional File 4: Presentation to stakeholder advisory group following questionnaire analysis.

Additional File 5: Pre-focus group material.

Additional File 6: Post-Focus group online questionnaire.

Additional File 7: Supplementary tables and figures.

## Data Availability

Consent to share individual level data was not sought in this project. Therefore, only reasonable requests of aggregate data and anonymised quotes will be considered.

## Abbreviations

CHERRIES: Checklist for Reporting Results of Internet E-Surveys
COREQ: Consolidated criteria for reporting qualitative research
GDPR: General Data Protection Regulation
HTA: Health Technology Assessment
IPCW: Inverse proportional censoring weighting
IPTW: Inverse proportional treatment weighting
IQR: Inter-quartile range
NHS: National Health Service
NI: Northern Ireland
OR: Odds ratio
OS: Overall survival
PIS: Patient Information Sheet
PPI: Patient and Public Involvement
RAM: RAND/UCLA appropriateness method
RCT: Randomised clinical trial
RPSFTM: Rank preserving structural failure time model
SAG: Stakeholder Advisory Group
SMART: Sequential, Multiple Assignment, Randomized Trial
UK: United Kingdom

## References

[1] FDA. Clinical Trial Endpoints for the Approval of Cancer Drugs and Biologics Guidance for Industry. 2018. https://www.fda.gov/media/71195/download. Accessed 6th Sep 2022.

[2] EMA. ICH E9 (R1) addendum on estimands and sensitivity analysis in clinical trials to the guideline on statistical principles for clinical trials. 2017 https://www.ema.europa.eu/en/documents/scientific-guideline/draft-ich-e9-r1-addendum-estimands-sensitivity-analysis-clinical-trials-guideline-statistical_en.pdf. Accessed 1st Mar 2023.

[3] Fontrier AM, Visintin E, Kanavos P. Similarities and differences in health technology assessment systems and implications for coverage decisions: evidence from 32 countries. Pharmacoeconomics. 2022;6:315–28. 10.1007/s41669-021-00311-5. 10.1007/s41669-021-00311-5 PMC9043057 34845671

[4] Latimer NR and Abrams KR. NICE DSU Technical Support Document 16: Adjusting survival time estimates in the presence of treatment switching. 2014. 27466662

[5] Royle K-L, Meads D, Visser-Rogers JK, et al. How is overall survival assessed in randomised clinical trials in cancer and are subsequent treatment lines considered? A systematic review. Trials. 2023;24:708. 37926806 10.1186/s13063-023-07730-1 PMC10626781

[6] Fitch K, Bernstein SJ, Aguilar MD. The RAND/UCLA appropriateness method user’s manual. Santa Monica CA: Rand Corp; 2001.

[7] Eysenbach G. Improving the quality of web surveys: the checklist for reporting results of internet e-surveys (CHERRIES). J Med Internet Res. 2004;6:e34. 10.2196/jmir.6.3.e34. 15471760 10.2196/jmir.6.3.e34 PMC1550605

[8] Tong A, Sainsbury P, Craig J. Consolidated criteria for reporting qualitative research (COREQ): a 32-item checklist for interviews and focus groups. Int J Qual Health Care. 2007;19:349–57. 10.1093/intqhc/mzm042. 17872937 10.1093/intqhc/mzm042

[9] SOLVE Study. SOLVE Summary Video, https://youtu.be/HKYbl8vcCaw. 19 May 2022.

[10] SOLVE Study. What is the SOLVE Study?, https://youtu.be/dZsle9e-NpI. 11 May 2022.

[11] SOLVE Study. How will the SOLVE study answer it's question?, https://youtu.be/AdfS1FeBhBs. 11 May 2022.

[12] Hsieh H-F, Shannon SE. Three approaches to qualitative content analysis. Qual Health Res. 2005;15:1277–88. 10.1177/1049732305276687. 16204405 10.1177/1049732305276687

[13] Kidwell KM, Almirall D. Sequential, multiple assignment, randomized trial designs. JAMA. 2023;329:336–7. 10.1001/jama.2022.24324. 36692577 10.1001/jama.2022.24324 PMC10061579

[14] Office for National Statistics. Ethnic group, national identity and religion, https://www.ons.gov.uk/methodology/classificationsandstandards/measuringequality/ethnicgroupnationalidentityandreligion. Accessed 17th Oct 2023.

[15] Flight L, Julious S, Brennan A, et al. How can health economics be used in the design and analysis of adaptive clinical trials? A qualitative analysis. Trials. 2020;21:252. 10.1186/s13063-020-4137-2. 32143728 10.1186/s13063-020-4137-2 PMC7060544

[16] Beatriz G, Camille P, Katie G. Patient and public involvement in numerical aspects of trials: a mixed methods theory-informed survey of trialists’ current practices, barriers and facilitators. BMJ Open. 2021;11:e046977. 10.1136/bmjopen-2020-046977. 10.1136/bmjopen-2020-046977 PMC7978289 33737444

[17] Goulao B, Bruhn H, Campbell M, et al. Patient and public involvement in numerical aspects of trials (PoINT): exploring patient and public partners experiences and identifying stakeholder priorities. Trials. 2021;22:499. 10.1186/s13063-021-05451-x. 34321066 10.1186/s13063-021-05451-x PMC8316879

