# Supplementary material for: How should overall survival be analysed in randomised clinical trials in cancer if participants receive subsequent treatment lines? A stakeholder consultation
Source: Trials. 2025 Oct 24;26:434. doi: 10.1186/s13063-025-09148-3 (PMC12551141; doi:10.1186/s13063-025-09148-3)
Supplement: Supplementary file 3 — Additional File 3: Patient Information Sheet (PIS) and Online Questionnaire. [file 13063_2025_9148_MOESM3_ESM.pdf]

SOLVE: Assessing Overall Survival - Can we do it better?

Introduction

Thank you for your interest in this online questionnaire.

One in two people will get cancer in their lifetime. This questionnaire forms part of a wider piece of research called SOLVE which is looking at how we can do cancer research better.

The questionnaire has a total of 25 questions and should take no more than 30 minutes to complete.

Unless you choose to provide your details, your answers will remain anonymous.

There are four sections to help you to decide if you would like to complete the questionnaire:

1. What is the purpose of the questionnaire?
2. What does the questionnaire look like?
3. Frequently Asked Questions
4. Data Protection

Please read all four sections and then decide if you would like to continue onto the questionnaire.

If at any point you have any questions, please contact the Chief Investigator Kara-Louise Royle at SOLVE@leeds.ac.uk.

1. What is the purpose of the questionnaire?

Less info

A clinical trial finds out whether or not a new or different treatment works better than any existing treatment. To do this, patients with the illness under study are carefully selected and monitored during the clinical trial period. Patients taking part in the clinical trial, known as participants, will either receive the new treatment, called the experimental treatment, or what is currently used in practice, called the control treatment.

In cancer clinical trials, we use a measure called “overall survival” along with statistics to compare these treatments. “Overall survival” is the length of time that the participant remains alive, from the point at which they start their clinical trial treatment.

However, not all cancers can be controlled with one type or ‘line’ of treatment. A patient may need a new line of treatment if:

1. They experience side effects

2. Their cancer starts to grow or spread to different part of the body (relapse)

If this happens, participants will usually stop their current clinical trial treatment and may start a different treatment. This next treatment is no longer decided by the clinical trial and could be different for all participants.

This means that instead of having one type of anti-cancer treatment in the period in which overall survival is measured, participants may have multiple treatments; the treatment which they received during the trial (“on-trial”) and one or more which they received after they completed their trial treatment (“off-trial”). In the assessment of “overall survival” we are interested in the difference in outcome, if any, between the two types (experimental or control) of “on-trial” treatment.

However, the current statistical methods available to analyse “overall survival”, do not account for multiple lines of treatment. Therefore, the effects of the “on-trial” and “off-trial” treatment are combined. This makes it difficult to work out what effect each line of treatment has on “overall survival” and in turn know whether a difference between the “on-trial” treatments exists.

The aim of SOLVE is to develop a statistical method which assesses “overall survival”, whilst taking into account any effect of the “off-trial” treatments. It is hoped that this method will provide useful information to help:

- Healthcare professionals discussing new treatment options with patients

- Decision makers to detemine whether a new treatment should be recomended for use in the NHS

- Patients and Carers make informed decisions on which treatment they should receive next

This questionnaire forms the first part of SOLVE. It aims to find out what people think about the different ways we can assess “overall survival” in clinical trials.

You can listen to the Chief Investigator, Kara-Louse Royle, explain the research below:

https://youtu.be/HKYbI8vcCaw

Click on "More Info" to find out!

2. What does the questionnaire look like?

Less info

The questionnaire has 25 questions and should take you no more than 30 minutes to complete.

There are five sections in the questionnaire:

1. Demographics: This asks your age, sex, and ethnicity to help us to know if the questionnaire is inclusive and to ensure we are reaching as many sections of the community as possible

2. Question of Interest: This asks you to think about what you might like to know about “overall survival”

3. Information Required: This asks you to choose from examples what information we should record in a clinical trial after a participant has stopped their trial treatment

4. Assumptions: This asks you to choose from examples, what things we should take for granted when we think about “overall survival”

5. Answer: This asks you about how we should show the results of a clinical trial

At the end of the questionnaire there is the option to sign-up for further communication and to discuss your answers to the questionnaire if you would like to.

The questionnaire has different types of questions there are:

Statements to rate in terms of whether you agree with them.

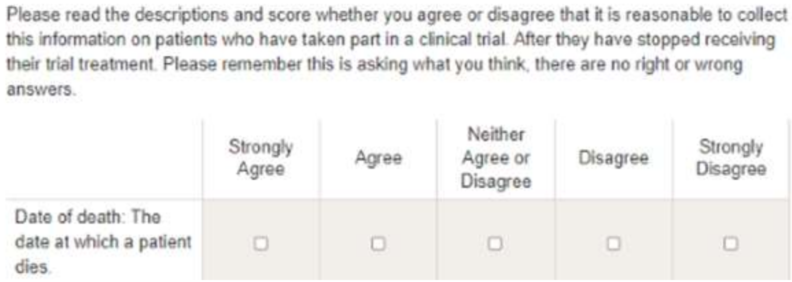
Please read the descriptions and score whether you agree or disagree that it is reasonable to collect this information on patients who have taken part in a clinical trial. After they have stopped receiving their trial treatment. Please remember this is asking what you think, there are no right or wrong answers.

	Strongly Agree	Agree	Neither Agree or Disagree	Disagree	Strongly Disagree
Date of death: The date at which a patient dies.	☐	☐	☐	☐	☐

Questions which ask you to choose one or multiple categories

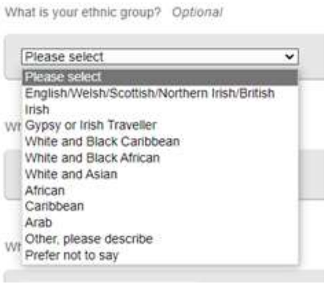
What is your ethnic group? *Optional*

Please select

- Please select
- English/Welsh/Scottish/Northern Irish/British
- Irish
- Gypsy or Irish Traveller
- White and Black Caribbean
- White and Black African
- White and Asian
- African
- Caribbean
- Arab
- Other, please describe
- Prefer not to say

Questions which ask you to respond with a few words or a sentence

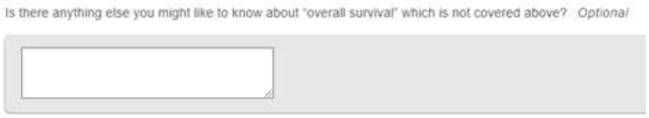
Is there anything else you might like to know about “overall survival” which is not covered above? *Optional*

There is only one question which you must answer. This question, shown in the picture below, asks you to decide which group you identify with the most based on the definitions. You may feel you fit into more than one group but please choose the one you think is most appropriate. The reason that this question is required is because some of the wording of the later questions change to make them more relevant to you.

Are you? Click on "More Info" for the definitions of each group. **Required**

Less info

A Member of the Public

For those who have not.

- Treated patients with cancer

- Analysed a cancer clinical trial

- Discussed whether a treatment should be recommended for use on the NHS

- Been diagnosed with cancer or cared for a family or close friend with cancer

But are interested to learn more about cancer clinical trials and/or have a say in how they work and how their results are explained to your family, friends, and the public. If you are not sure which group to pick, everyone is a member of the public.

A Patient or Carer

For those who have cancer or have cared for someone with cancer and are interested to learn more about cancer clinical trials and/or have a say in how they work and how the results are explained to your family, friends, and the public.

A Health Professional

For those with a medical background who have experience in treating or discussing cancer treatment options with patients.

An Industry Partner

For those who are employed by the pharmaceutical industry and work in the development of cancer treatments.

A Payer

For those with experience in evaluating whether a new cancer treatment or intervention should be recommended for use in standard practice. For example, you are a member of a technology appraisal committee.

A Statistician or Other Data Analyst

Fo[illegible] l background with experience in analysing cancer clinical tria[illegible] Data Scientists.

Yo[illegible] up but please choose the one you think is most ap[illegible]

- Please select
- A Member of the Public
- A Patient or Carer
- A Health Professional
- An Industry Partner
- A Payer
- A Statistician or Other Data Analyst

Please select

You may feel you fit into more than one group but please choose the one you think is most appropriate. The reason that this question is required is because some of the wording of the later questions change to make them more relevant to you.

Click on "More Info" to find out!

3. Frequently Asked Questions

Click on each of the questions below to find the answers to some questions you may have about completing the questionnaire.

Do I have to take part?

Less info

No, the questionnaire is completely voluntary, and you do not have to complete it.

Click on "More Info" for the answer!

What do I have to do to take part?

Less info

If you want to complete the questionnaire you need to first provide informed consent. This is a series of statements you need to tick that you agree with. Informed consent is our way to make sure that you have read and understood all the information that we have provided and are happy to proceed.

Click on "More Info" for the answer

I have no prior experience of research, can I still take part?

Less info

Yes! The questionnaire has been created considering that a lot of people will not have thought about research before. However, if you have any questions about what some of the statements mean please do get in touch with the Chief Investigator Kara-Louise Royle via email SOLVE@leeds.ac.uk. This questionnaire is all about finding out opinions so there are no right or wrong answers.

Click on "More Info" to find out!

What are the possible benefits of taking part?

Less info

We need your opinions so we can enable patients be more informed about their treatment choices, and help decision makers on whether a new treatment should be approved for use in the NHS.

Click on "More Info" to find out!

What are the possible disadvantages and risks of taking part?

Less info

We do not anticipate any risks or disadvantages to you in completing the questionnaire. There will be a small-time commitment of approximately 30 minutes, and you are able to stop completing it at any point. However, in the questionnaire we are thinking about death and cancer. These are sensitive topics which may cause you to experience emotional distress. If at any point you become uncomfortable or upset, you are free to stop completing the questionnaire. If you feel you need support, we recommend talking to your local Patient Advice and Liaison Service (PALS) to identify what support is available. Charities such as Macmillan, also have support helplines you can talk to about cancer.

Click on "More Info" to find out!

Will I get any reimbursement or financial incentive for taking part?

Less info

Unfortunately, we are unable to offer any reward for completing the questionnaire.

Click on "More Info" to find out!

What will happen after I have taken part?

Less info

The answers you give to the questionnaire based on your opinions, will give us a better idea about what everyone thinks about how “overall survival” should be assessed. The information will be collated and used to guide discussion groups. More information about the discussion groups is at the end of the questionnaire. Unless you choose to provide your contact details, your answers to the questionnaire will remain anonymous.

Click on "More Info" to find out!

Do I have to complete the questionnaire in one go?

Less info

No. You will have the option to save your answers online and continue at a more convenient time. However, we encourage you, if possible, to complete the questionnaire in one sitting.

Click on "More Info" to find out!

Can I ask you to delete my questionnaire responses after I have completed it?

Less info

Unfortunately, once you have submitted your questionnaire, we will not be able to delete your individual responses until the end of the research.

Click "More Info" to find out!

Can I share the information about this questionnaire with others?

Less info

Yes! We would encourage you to share the questionnaire with your colleagues, friends and relatives if you wish. However, they will also need to provide informed consent before they take part.

Click on "More Info" to find out!

Can I stop taking part after I have consented?

Less info

Yes. If you decide partway through completing the questionnaire that you do not want to take part anymore. Close the questionnaire and do not submit your answers. Any answers you have already entered will not be saved.

Click "More Info" to find out!

Is there a paper version of the questionnaire?

Less info

No. This is an online questionnaire only as there are links to videos in the questionnaire which could not be played on a paper version.

Click "More Info" to find out!

4. Data Protection

Less info

All data will be protected as required by UK law, including GDPR. If you need further information, the University of Leeds privacy notice can be found here: https://dataprotection.leeds.ac.uk/wp-content/uploads/sites/48/2019/02/Research-Privacy-Notice.pdf. The university also has a data protection officer who can be contacted at: dpo@leeds.ac.uk if you have further concerns.

The following sections answer some of the questions you may have around what happens to your data collected during the research.

How will my data be protected?

Less info

Data collected as part of this research will be stored securely on either the online survey database or on University of Leeds network computers with access restricted to the research team. Any results will be presented in summaries, conference presentations and journal articles in such a way that you cannot be identified.

Click "More Info" to find out!

Who has organised and sponsored the research?

Less info

The research is being organised and coordinated by the Leeds Institute of Clinical Trials Research at the University of Leeds, who is sponsoring the research.

Click "More Info" to find out!

Who has reviewed the research?

Less info

This research is being undertaken as part of a PhD degree. It has been reviewed and funded by the NIHR (National Institute of Health Research) under their Doctoral Fellowship funding stream. It has also been reviewed by the Chief Investigator's PhD supervisors. Finally, it has been reviewed and approved by an NHS Research Ethics Committee 22/YH/0155, IRAS Project ID 299766.

Click "More Info" to find out!

How long will you keep my data?

Less info

Data will be kept for the length of the research (5 years) and archived on university systems for an additional 15 years as per sponsor requirements.

Click "More Info" to find out!

If you are happy with all the information provided please continue on to the next page to provide informed consent.

Informed Consent

The following points should be completed by the individual completing the questionnaire and are required to access the questionnaire.

I confirm that I have read and understand the information provided. I have had the opportunity to ask questions over email and where applicable have had these answered satisfactorily. ✱ *Required*

○ Yes

I understand that completing the questionnaire is voluntary and that I am free to stop at any time without giving a reason. ✱ *Required*

○ Yes

I agree to allow any information or results arising from the questionnaire to be used for training and developing new research where my identity will remain anonymous. ✱ *Required*

○ Yes

I understand that my questionnaire may be looked at by responsible individuals from the research team where it is relevant to my taking part in the research. I give permission for these individuals to have access to my information and data, where my identity will remain anonymous. ✱ *Required*

○ Yes

I understand that the information I provide will be summarised and presented alongside other participants information at meetings and in publications relating to the research without me being identified. ✱ *Required*

○ Yes

I confirm that I am aged 18 or over. ✱ *Required*

○ Yes

I agree to complete the questionnaire ✱ *Required*

○ Yes

Demographics

The following questions are to help gather information about who is competing the questionnaire so we can be sure we have a wide range of opinions.

The first set of questions are generic questions that you may have answered in a questionnaire before.

How old are you? *Optional*

[ ]

What is your ethnic group? *Optional*

[ ]

If you selected Other, please specify:

[ ]

What sex are you? *Optional*

○ Male ○ Female ○ Prefer not to say

Where in the UK are you based? *Optional*

[ ]

If you selected Other, please specify:

[ ]

Where did you find out about this questionnaire? *Optional*

☐ Mailing List ☐ Social Media ☐ Word of Mouth
☐ Other

If you selected Mailing List, please specify

[ ]

If you selected Other, please specify:

[ ]

The next set of questions on this page are specific to this questionnaire and aim to find out what information you may or may not know about clinical trials and assessing “overall survival”. There are no right or wrong answers.

Are you? Click on "More Info" for the definitions of each group. ✱ *Required*

Less info

A Member of the Public

For those who have not.

- Treated patients with cancer

- Analysed a cancer clinical trial

- Discussed whether a treatment should be recommended for use on the NHS

- Been diagnosed with cancer or cared for a family or close friend with cancer

But are interested to learn more about cancer clinical trials and/or have a say in how they work and how their results are explained to your family, friends, and the public. If you are not sure which group to pick, everyone is a member of the public.

A Patient or Carer

For those who have cancer or have cared for someone with cancer and are interested to learn more about cancer clinical trials and/or have a say in how they work and how the results are explained to your family, friends, and the public.

A Health Professional

For those with a medical background who have experience in treating or discussing cancer treatment options with patients.

An Industry Partner

For those who are employed by the pharmaceutical industry and work in the development of cancer treatments.

A Payer

For those with experience in evaluating whether a new cancer treatment or intervention should be recommended for use in standard practice. For example, you are a member of a technology appraisal committee.

A Statistician or Other Data Analyst

For those with a statistical or mathematical background with experience in analysing cancer clinical trial data. Includes Health Economists and Data Scientists.

You may feel you fit into more than one group but please choose the one you think is most appropriate.

You may feel you fit into more than one group but please choose the one you think is most appropriate. The reason that this question is required is because some of the wording of the later questions change to make them more relevant to you.

Have you had any prior experience of clinical trials? *Optional*

- ○ Yes
- ○ No

If Yes...

- ☐ I joined and took part in a clinical trial
- ☐ A friend / family member was part of a clinical trial
- ☐ I have been a patient and public (PPI) contributor on a clinical trial
- ☐ I have been a Principal Investigator or Trial Management Group member in a clinical role
- ☐ I have been a Statistician in a professional capacity
- ☐ I have been a Health Economist in a professional capacity
- ☐ I have been a Data or Trial Manager in a professional capacity
- ☐ Other

If you selected Other, please specify:

Do you think it is important to consider the effect that treatment given after trial treatment has on "overall survival"? *Optional*

- ○ Yes
- ○ No
- ○ Depends
- ○ Unsure

Please explain your answer

Before today have you ever thought about how after trial treatment might affect "overall survival"? *Optional*

- ○ Yes
- ○ No

Please explain your answer

Question of Interest - Healthcare Professionals

Suppose you need to discuss the possibility a new treatment with a patient which aims to improve survival. To aid your discussion you have the results of a clinical trial which compared the new treatment to the previous standard of care. However, not all participants in the trial received the treatment until death. Participants in the trial could only stay on the treatment for a finite period of time due to trial follow-up, stopping due to toxicity, participant or clinician choice or disease progression. After this point their treatment was no longer specified by a clinical trial protocol and may have included no treatment or some treatment which could have been standard or care or other experimental treatments if they joined another clinical trial.

In your opinion which of the following questions would be the most interesting to know the answer to? Please rank them from most interesting (1) to least interesting (4).

	1 (Most Interested)	2	3	4 (Least Interested)
How does the new treatment extend survival compared to the control treatment; even though some participants stopped their trial treatment prior to death?	☐	☐	☐	☐
How would the new treatment have extended survival compared to the control treatment, if no one stopped their trial treatment prior to death?	☐	☐	☐	☐
How does the new treatment extend survival compared to the control treatment, in participants who only received their trial treatment prior to death?	☐	☐	☐	☐
How much longer did participants stay on the experimental treatment compared to the control treatment?	☐	☐	☐	☐

This video explains each question in a little more detail: https://youtu.be/wSgO3x5QyTg

Can you think of any scenarios where the question you are most interested in would change? *Optional*

○ Yes ○ No

Please explain your answer

Is there anything else you might like to know about “overall survival” which is not covered above? *Optional*

Question of Interest - Payers and Industry Partners

Suppose you need to assess whether a new treatment should be recommended for use in standard practice based on the analysis of “overall survival” from clinical trial data. However, not all participants in the trial received the treatment until death. Participants in the trial could only stay on the treatment for a finite period of time due to trial follow-up, stopping due to toxicity, participant or clinician choice or disease progression. After this point their treatment no longer followed a clinical trial protocol and may have included no treatment or some treatment which could have been standard or care or other experimental treatments if they joined another clinical trial.

In your opinion which of the following questions would be the most interesting to know the answer to? Please rank them from most interesting (1) to least interesting (4).

	1 (Most Interested)	2	3	4 (Least Interested)
How does the new treatment extend survival compared to the control treatment; even though some participants stopped their trial treatment prior to death?	☐	☐	☐	☐
How would the new treatment have extended survival compared to the control treatment, if no one stopped their trial treatment prior to death?	☐	☐	☐	☐
How does the new treatment extend survival compared to the control treatment, in participants who only received their trial treatment prior to death?	☐	☐	☐	☐
How much longer did participants stay on the experimental treatment compared to the control treatment?	☐	☐	☐	☐

This video explains each question in a little more detail: https://youtu.be/wSgO3x5QyTg

Can you think of any scenarios where the question you are most interested in would change? *Optional*

○ Yes ○ No

Please explain your answer

Is there anything else you might like to know about “overall survival” which is not covered above? *Optional*

Additional Questions - Statisticians and Data Analysts

The following questions are specific things we wish to find out from statisticians and other data analysts like yourself.

What statistical software do you use the most? *Optional*

- ○ SAS
- ○ R
- ○ STATA
- ○ SPSS
- ○ Other

If you selected Other, please specify:

Thinking about the trials you have worked on, what data is collected on trial participants during follow-up once they have completed their trial treatment? *Optional*

- ☐ Post-trial treatment
- ☐ Progression date
- ☐ Disease Characteristics
- ☐ Participation in clinical trials (trial name / registry number)
- ☐ Participation in clinical trials (Participant ID)
- ☐ Date of Death
- ☐ Cause of Death
- ☐ Other

If you selected Other, please specify:

Please summarise your experience of the following statistical methods in relation to the analysis of "overall survival" *Optional*

	Never heard of it	Heard of it but not applied	Applied
Logrank Test	☐	☐	☐
Cox Proportional Hazard Model	☐	☐	☐
Rank Preserving Structural Failure Time Model	☐	☐	☐
Inverse Probability of Censoring Weights	☐	☐	☐
Inverse Probability of Treatment Weights	☐	☐	☐
Two-Stage Method	☐	☐	☐
g-methods	☐	☐	☐

If you wish to, please elaborate on your answer. Particularly if you have heard of a method but not applied it, please add why. *Optional*

Have you ever adjusted "overall survival" analysis to account for subsequent lines of treatment? *Optional*

○ Yes ○ No

If yes, what method(s) did you use?

Question of Interest - Statisticians and Data Analysts

Suppose you need to analyse the effect of a new treatment, compared to the current standard of care on “overall survival” from clinical trial data. However, not all participants in the trial received the treatment continuously until death or the end of follow-up. Some participants stopped their trial treatment due to either:

1. Toxicity
2. Participant or Clinician Choice
3. Disease progression / relapse

After this point their treatment was no longer specified by the clinical trial protocol and may have included no treatment or some treatment which could have been standard of care, or other experimental treatments, if they joined another clinical trial.

Stopping treatment can be described as an intercurrent event. There are several different ways one can deal with an intercurrent event in an analysis. However, each way changes the question being asked. For each possible scenario we have written the question which an analysis could answer. Please consider each one and rank them in order of which you think would be the most interesting (1) to answer to least interesting (4) to answer. *Optional*

	1 (Most Interesting)	2	3	4 (Least Interesting)
How does the new treatment extend survival compared to the control treatment; even though some participants stopped their trial treatment prior to death?	☐	☐	☐	☐
How would the new treatment have extended survival compared to the control treatment, if no one stopped their trial treatment prior to death?	☐	☐	☐	☐
How does the new treatment extend survival compared to the control treatment, in participants who only received their trial treatment prior to death?	☐	☐	☐	☐
How much longer did participants stay on the experimental treatment compared to the control treatment?	☐	☐	☐	☐

This video explains each question in a little more detail: https://youtu.be/wSgO3x5QyTg

Can you think of any scenarios where the question you are most interested in would change? *Optional*

○ Yes ○ No

Please explain your answer

Are there any other questions you might like to know the answer to when analysing “overall survival” which are not covered above? *Optional*

Question of Interest - Patients, Carers and the Public

Suppose you, a family member or a friend have cancer and are offered a new treatment which aims to increase how long you, or they live.

The new treatment was compared to another treatment in a clinical trial that included patients with the same cancer. In the clinical trial patients either stayed on treatment until:

1. They passed away
2. They did not want to take the treatment anymore
3. Their cancer continued to grow

If a patient stopped taking their trial treatment they were offered different treatments by their doctor.

To help you to decide whether you, a family member or friend, should have the new treatment you can ask questions about the results of the trial.

You might have many questions about different things such as how does the new treatment affect a patients Quality of Life. Thinking specifically about how long the new treatment kept people alive for compared to the other treatment. Please read the five questions below and rank them in order of what would be the most important for you to know the answer to (1) to the least important (4) if you or a friend were being treated for cancer. Please remember this is asking what you think, there are no right or wrong answers. *Optional*

	1 (Most Important)	2	3	4 (Least Important)
In the clinical trial did the new treatment extend patient's lives even though they did not receive it forever?	☐	☐	☐	☐
In the clinical trial if everyone had stayed on their trial treatment, would the new treatment have extended patient's lives?	☐	☐	☐	☐
In the clinical trial did the new treatment extend patient's lives if they only received their trial treatment?	☐	☐	☐	☐
In the clinical trial did the new treatment increase how long people stayed on treatment for?	☐	☐	☐	☐

This video explains each question in a little more detail: https://youtu.be/JVbAjIKppLo

Can you think of any scenarios where the question you think is most important would change? *Optional*

○ Yes ○ No

Please explain your answer

Can you think of any other questions you might want to ask about the results of the clinical trial in terms of whether the new treatment extended patient's lives? *Optional*

Information Required - Health Care Professionals, Payers and Industry Partners

To answer research questions about survival accurately, we need to collect information on participants in a clinical trial.

Please consider each piece of information listed below and decide whether you agree it is practical and appropriate to collect this information about clinical trial participants, after they have stopped receiving their trial treatment. *Optional*

Please don't select more than 1 answer(s) per row.

	Strongly Agree	Agree	Neither Agree or Disagree	Disagree	Strongly Disagree
Date of death: The date at which a patient dies.	☐	☐	☐	☐	☐
Cause of death: The reason the patient died.	☐	☐	☐	☐	☐
Date(s) of progression / relapse: The date the patient's cancer no longer responds to their current treatment	☐	☐	☐	☐	☐
Anti-cancer treatment: The name of any further treatment the patient has to treat their cancer once they stopped their trial treatment and the dates they had it.	☐	☐	☐	☐	☐
Patient Characteristics: Information about the patient such as their height and weight. Measurements which are not necessarily about their cancer.	☐	☐	☐	☐	☐
Disease Characteristics: Information about a patient's cancer such as the severity of their cancer.	☐	☐	☐	☐	☐

If you would like to, please explain your answers

Is there anything other information you think we should consider collecting? *Optional*

There are two different ways this information can be collected from patients who have stopped their trial treatment. Which do you think is the most appropriate? *Optional*

- ○ At a trial follow-up appointment. This appointment could be in person or over the phone and would be an additional appointment to the ones the patient was attending as part of their off-trial treatment.
- ○ At a routine appointment. This appointment could be in person or over the phone and would be an appointment which the patient attended as part of their off-trial treatment.

If you would like to, please explain your answer

There are also two different ways this information can be recorded and returned to the trial researchers. Which do you think is the most appropriate? *Optional*

- ○ From a database which was made specifically for the trial. This makes sure everything is recorded in the same way.
- ○ From a database which is completed normally as part of standard practice. This means that information isn't duplicated across databases.

If you would like to, please explain your answer

If you wish to, please provide your reasoning for your answers to the above questions *Optional*

Information Required - Statisticians and Data Analysts

To analyse "overall survival" accurately, we need to collect information on participants in a clinical trial.

Please consider each piece of information listed below and decide whether you agree it is practical and appropriate to collect this information about clinical trial participants, after they have stopped receiving their trial treatment.

	Strongly Agree	Agree	Neither Agree or Disagree	Disagree	Strongly Disagree
Date of death: The date at which a patient dies.	☐	☐	☐	☐	☐
Cause of death: The reason the patient died.	☐	☐	☐	☐	☐
Date(s) of progression / relapse: The date the patient's cancer no longer responds to their current treatment	☐	☐	☐	☐	☐
Anti-cancer treatment: The name of any further treatment the patient has to treat their cancer once they stopped their trial treatment and the dates they had it.	☐	☐	☐	☐	☐
Patient Characteristics: Information about the patient such as their height and weight. Measurements which are not necessarily about their cancer.	☐	☐	☐	☐	☐
Disease Characteristics: Information about a patient's cancer such as the severity of their cancer.	☐	☐	☐	☐	☐

If you would like to, please explain your answers

Is there anything other information you think we should consider collecting? *Optional*

There are two different ways this information can be collected from patients who have stopped their trial treatment. Which do you think is the most appropriate? *Optional*

- ○ At a trial follow-up appointment. This appointment could be in person or over the phone and would be an additional appointment to the ones the patient was attending as part of their off-trial treatment.
- ○ At a routine appointment. This appointment could be in person or over the phone and would be an appointment which the patient attended as part of their off-trial treatment.

If you would like to, please explain your answer

There are also two different ways this information can be recorded and returned to the trial researchers. Which do you think is the most appropriate? *Optional*

- ○ From a database which was made specifically for the trial. This makes sure everything is recorded in the same way.
- ○ From a database which is completed normally as part of standard practice. This means that information isn't duplicated across databases.

If you would like to, please explain your answer

Information Required - Patients, Carers and the Public

To answer research questions accurately, we need to collect information on patients in a clinical trial. Listed below are some of the pieces of information we may need to collect to find out if the new treatment extended patient's lives.

Please read the descriptions and score whether you agree or disagree that it is reasonable to collect this information on patients who have taken part in a clinical trial. After they have stopped receiving their trial treatment. Please remember this is asking what you think, there are no right or wrong answers.

	Strongly Agree	Agree	Neither Agree or Disagree	Disagree	Strongly Disagree
Date of death: The date at which a patient dies.	☐	☐	☐	☐	☐
Cause of death: The reason the patient died.	☐	☐	☐	☐	☐
Date(s) of progression / relapse: The date the patient's cancer no longer responds to their current treatment	☐	☐	☐	☐	☐
Anti-cancer treatment: The name of any further treatment the patient has to treat their cancer once they stopped their trial treatment and the dates they had it.	☐	☐	☐	☐	☐
Patient Characteristics: Information about the patient such as their height and weight. Measurements which are not necessarily about their cancer.	☐	☐	☐	☐	☐
Disease Characteristics: Information about a patient's cancer such as the severity of their cancer.	☐	☐	☐	☐	☐

If you would like to, please explain your answers *Optional*

Is there anything other information you think we should consider collecting? *Optional*

There are two different ways this information can be collected from patients who have stopped their trial treatment. Which do you think is the most appropriate? *Optional*

- ○ At a trial follow-up appointment. This appointment could be in person or over the phone and would be an additional appointment to the ones the patient was attending as part of their off-trial treatment.
- ○ At a routine appointment. This appointment could be in person or over the phone and would be an appointment which the patient attended as part of their off-trial treatment.

If you would like to, please explain your answer

There are also two different ways this information can be recorded and returned to the trial researchers. Which do you think is the most appropriate? *Optional*

- ○ From a database which was made specifically for the trial. This makes sure everything is recorded in the same way.
- ○ From a database which is completed normally as part of standard practice. This means that information isn't duplicated across databases.

If you would like to, please explain your answer

Assumptions - Statisticians, Health Care Professionals, Payers and Industry Partners

To apply statistical models, we have to specify some conditions which may or may not be true. We call these assumptions. The statistical models which are currently used in practice to analyse “overall survival” (e.g., the Kaplan-Meier method and Cox Regression) use assumptions.

Some of the assumptions which are currently used in some methods are listed below. Consider each one in turn and decide whether you think it is reasonable to make the assumption when we analyse “overall survival” in clinical trials where some participants stopped their trial treatment and received non-trial treatment during follow-up. This could be in all scenarios, in some scenarios, or in no scenario. Each assumption should be considered in its own right and there is the option to state that you are unsure.

Non-Informative Censoring (1) - A participant has stopped being followed-up. If we cannot find out information about a participant in the clinical trial at a certain time, their risk of dying is the same as a participant in the trial who we can find out information about. For example, if a participant in the clinical trial moves to another country and we cannot contact them, this does not make them more likely to die than a participant who has not moved to another country. *Optional*

○ All Scenarios ○ Some Scenarios ○ No Scenarios
○ Unsure

If you would like to, please explain your answer

Non-Informative Censoring (2) - A participant has stopped trial treatment. If a participant stops their trial treatment at a certain time, their risk of dying is the same as all participants in the trial who could stop their trial treatment at the same time but do not. For example, if a participant stops treatment at 6 months, this means that at 6 months they are not more likely to die than someone who does not stop treatment at 6 months. *Optional*

○ All Scenarios ○ Some Scenarios ○ No Scenarios
○ Unsure

If you would like to, please explain your answer

Proportional Hazards. At all times when information is being collected in the clinical trial, the chance of a participant on the current treatment (control group) dying is consistently a multiple of the chance of a participant on the new treatment (experimental group) dying. For example, we may say that at any time the chance of a participant dying on the current treatment is double the chance of a participant dying on the new treatment. *Optional*

○ All Scenarios ○ Some Scenarios ○ No Scenarios
○ Unsure

If you would like to, please explain your answer

Common Treatment Effect. If a participant receives the experimental treatment later on in their treatment pathway, it will have the same effect as it would have done if they had it at the start of the clinical trial. For example, we may say that whenever a participant has this treatment, it will always increase the time they are alive by 3 months. *Optional*

- ○ All Scenarios
- ○ Some Scenarios
- ○ No Scenarios
- ○ Unsure

If you would like to, please explain your answer

No Unmeasured Confounding. We know everything that could affect whether a participant stops their trial treatment, and whether they will die. i.e., we know the whole reason why a participant has stopped trial treatment and the whole reason why they died. *Optional*

- ○ All Scenarios
- ○ Some Scenarios
- ○ No Scenarios
- ○ Unsure

If you would like to, please explain your answer

No Time Dependent Covariates. All person and disease characteristics are collected once and do not change over time. For example, we only consider how fast the cancer is growing at the start of treatment and not how that changes once treatment has started. *Optional*

- ○ All Scenarios
- ○ Some Scenarios
- ○ No Scenarios
- ○ Unsure

If you would like to, please explain your answer

There are no competing risks. The reason why a participant dies is not important. The fact that they have died is the only thing that matters. For example, a participant may have died due to a different illness and not due to their cancer. *Optional*

- ○ All Scenarios
- ○ Some Scenarios
- ○ No Scenarios
- ○ Unsure

If you would like to, please explain your answer

Do you have anything else you would like to add or comment on about assumptions? *Optional*

Assumptions - Patients, Carers and the Public

To answer research questions using statistics, we have to specify some conditions which may or may not be true. We call these assumptions. The statistics we currently use to answer questions about how long a new treatment extends patient's lives have some assumptions. Some of these are described below along with their statistical name and an example. Some of the statistical names and definitions use words which may mean different things in everyday life. Click on "More Info" to find out what these mean statistically.

Consider each of the assumptions below in turn. For each one decide whether you think it is appropriate to make the assumption, when we answer questions about extending patient's lives, either in every circumstance, in some circumstances, or no circumstances. Each assumption should be considered in its own right. There is also the option to explain your answer or say that you are unsure.

Non-Informative Censoring (1) – A participant has stopped being followed-up. If we cannot find out information about a participant in the clinical trial at a certain time, their risk of dying is the same as a participant in the trial who we can find out information about. For example, if a participant in the clinical trial moves to another country and we cannot contact them, this does not make them more likely to pass away than a participant who has not moved to another country. *Optional*

○ All Circumstances ○ Some Circumstances ○ No Circumstances
○ Unsure

If you would like to, please explain your answer

Non-Informative Censoring (2) – A participant has stopped trial treatment. If a participant stops their trial treatment at a certain time, then they are alike (except for their next treatment) to all participants who could stop their trial treatment at the same time but do not. For example, if a participant stops treatment at 6 months, then we can say that at 6 months they are the same as everyone who doesn't stop treatment at 6 months. *Optional*

○ All Circumstances ○ Some Circumstances ○ No Circumstances
○ Unsure

If you would like to, please explain your answer

Proportional Hazards. At all times when information is being collected in the clinical trial, the chance of a participant on the current treatment (control group) dying is a multiple of the chance of a participant on the new treatment (experimental group) dying. For example, we may say that at any time the chance of a participant passing away on the current treatment is double the chance of a participant passing away on the new treatment. *Optional*

○ All Circumstances ○ Some Circumstances ○ No Circumstances
○ Unsure

If you would like to, please explain your answer

Common Treatment Effect. If a participant receives the experimental treatment later on in their treatment pathway, it will have the same effect as it would have done if they had it at the start of the clinical trial. For example, we may say that whenever a participant has this treatment, it will always increase the time they are alive by 3 months. *Optional*

○ All Circumstances ○ Some Circumstances ○ No Circumstances
○ Unsure

If you would like to, please explain your answer

No Unmeasured Confounding. We know everything that could affect whether a participant stops their trial treatment, and whether they will die. i.e., we know the whole reason why a participant has stopped trial treatment and the whole reason why they died. *Optional*

○ All Circumstances ○ Some Circumstances ○ No Circumstances
○ Unsure

If you would like to, please explain your answer

No Time Dependent Covariates. All person and disease characteristics are collected once and do not change over time. For example, we only consider how fast the cancer is growing at the start of treatment and not how that changes once treatment has started. *Optional*

○ All Circumstances ○ Some Circumstances ○ No Circumstances
○ Unsure

If you would like to, please explain your answer

There are no competing risks. The reason why a participant dies is not important. The fact that they have died is the only thing that matters. For example, a participant may have died due to a different illness and not due to their cancer. *Optional*

- ○ All Circumstances
- ○ Some Circumstances
- ○ No Circumstances
- ○ Unsure

If you would like to, please explain your answer

Do you have anything else you would like to add or comment on about assumptions? *Optional*

Answer - Statisticians, Health Care Professionals, Payers and Industry Partners

“Overall survival” results can be shown in several ways. Read the descriptions below and score whether presenting survival data in each of the following ways are helpful to you. *Optional*

	Very Helpful	Helpful	Neither Helpful or Unhelpful	Unhelpful	Very Unhelpful
Survival Curve: A picture showing how the proportion of participants alive on each treatment changes over time.	☐	☐	☐	☐	☐
Median time: The time when half of the people who were assigned to have each treatment were still alive.	☐	☐	☐	☐	☐
Mean Survival: The average time that the people who were assigned to have each treatment were still alive.	☐	☐	☐	☐	☐
Hazard Ratio: A ratio comparing the risk of a participant passing away on the new treatment compared to the other treatment.	☐	☐	☐	☐	☐

If you would like to, please explain your answers *Optional*

Each of the above can be supported by additional information. Consider the two types of additional information below and decide whether they are helpful to you. *Optional*

	Very Helpful	Helpful	Neither Helpful or Unhelpful	Unhelpful	Very Unhelpful
Confidence Interval: A range of answers for which the true answer lies with a percentage of uncertainty.	☐	☐	☐	☐	☐
P-value: The probability that the answer is due to chance.	☐	☐	☐	☐	☐

If you would like to, please explain your answers

Do you have anything else you would like to add or comment on about how best to show the answer to the question? *Optional*

Answer - Patients, Carers and the Public

The answer to "Does the new treatment extend life?" can be shown in several different ways. Read the descriptions below and decide whether presenting the answer in each way would help you to understand clinical trial results. Please remember this is asking what you think, there are no right or wrong answers.

Survival Curve: A picture showing how the proportion of participants alive on each treatment changes over time. As an example it

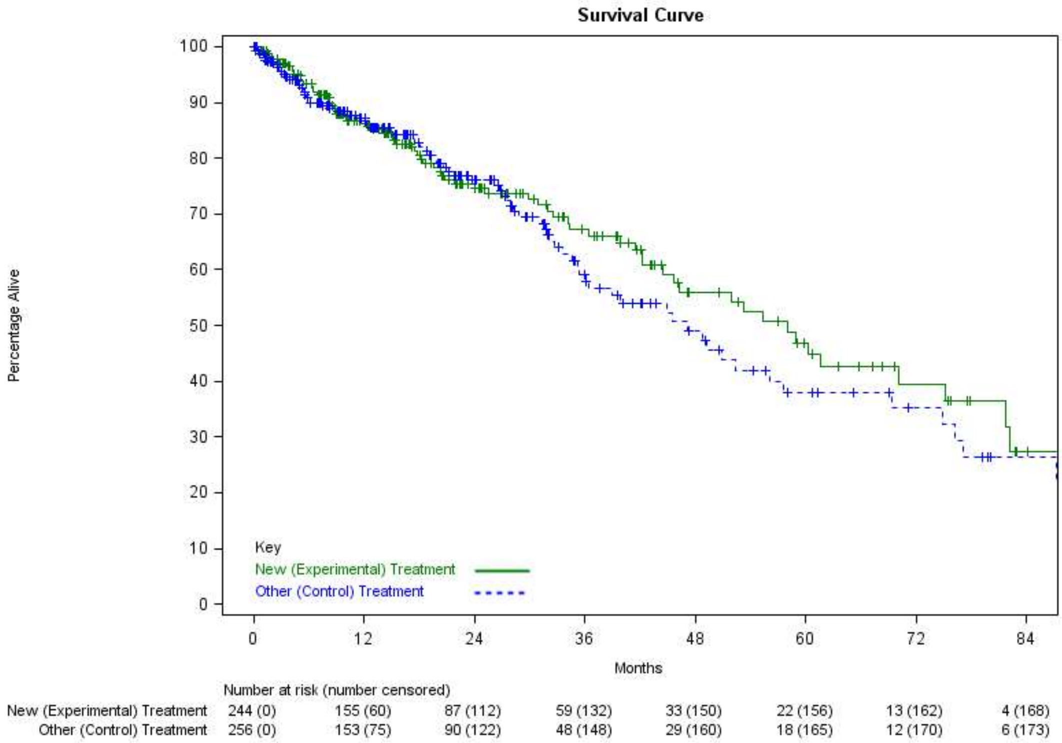

might look like this:
It starts off with everyone in each treatment group alive. So 100% of people are alive. Then, each time someone passes away the lines drops to show the percentage of people alive decreasing. Showing the lines in each group gives an idea of whether people are passing away faster in one treatment group as compared to the other. *Optional*

○ Very Helpful ○ Helpful ○ Neither Helpful or Unhelpful
○ Unhelpful ○ Very Unhelpful

If you would like to, please explain your answer

Median Survival: The time when half of the people who were assigned to have each treatment were still alive. This means that 50% of the people in each group are still alive. We can see this on the survival curve below.

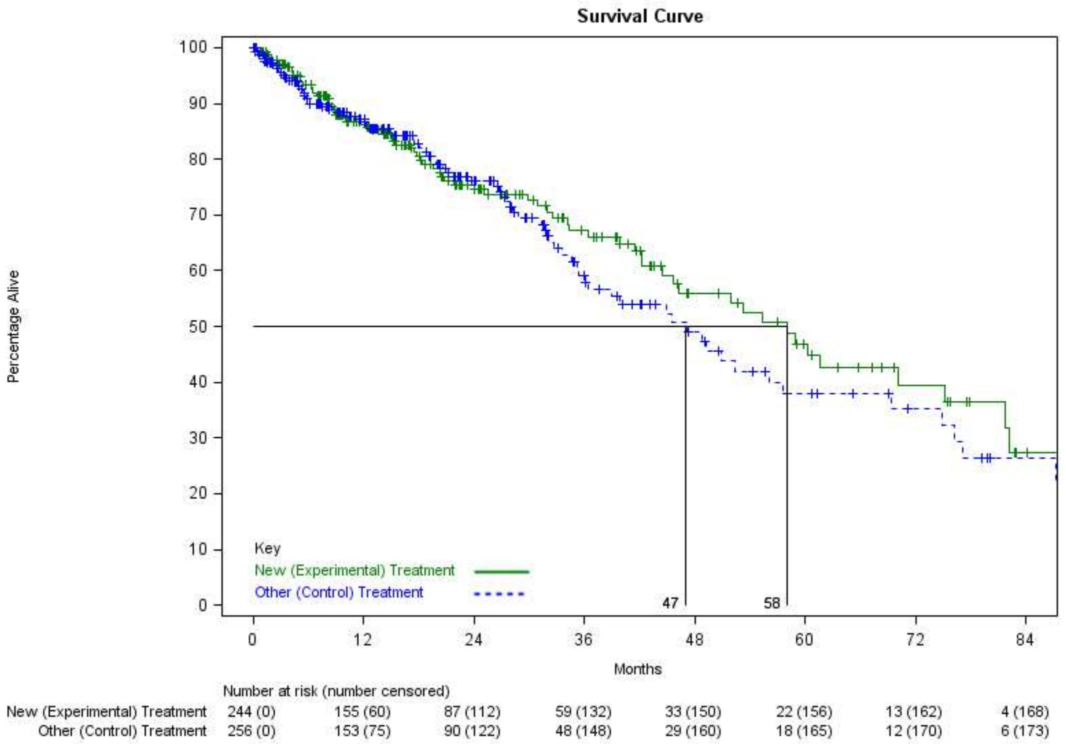

At 47 months half of the people in the control treatment group whereas at 58 months half of the people were still alive in the experimental group. *Optional*

○ Very Helpful ○ Helpful ○ Neither Helpful or Unhelpful
○ Unhelpful ○ Very Unhelpful

If you would like to, please explain your answer

Mean Survival: The average time that the people who were assigned to have each treatment were still alive. For example we may say that on average patients in the control arm were alive for 18 months but patients in the experimental arm were alive for 24 months. *Optional*

○ Very Helpful ○ Helpful ○ Neither Helpful or Unhelpful
○ Unhelpful ○ Very Unhelpful

If you would like to, please explain your answer

Hazard Ratio: A ratio comparing the risk of a participant passing away on the new (experimental) treatment compared to the other (control) treatment. A Hazard Ratio lower than 1 means that participants in the experimental treatment group have a lower chance of passing away compared to the control treatment. A Hazard Ratio greater than 1 means that participants in the experimental treatment group have a higher chance of passing away compared to the control treatment. For example a Hazard Ratio of 0.7 means that participants on the experimental treatment have a 30% reduced chance of passing away compared to participants on the control treatment. *Optional*

○ Very Helpful ○ Helpful ○ Neither Helpful or Unhelpful
○ Unhelpful ○ Very Unhelpful

If you would like to, please explain your answer

Each of the above can be supported by additional information. Consider the two types of additional information below and decide whether they are they are of interest to you.

Confidence Interval: A range of answers for which the true answer lies. Because we haven't included the whole population in our clinical trial our answer is only an estimate. A confidence interval tells us how close we are to the true answer. A wide confidence interval means we are not very close to the true answer. Whereas a narrow one means we are very close! A confidence interval also comes with a percentage that tells us how many times out of 100 we expect the true answer to be in the confidence interval, if we ran the exact same trial. For example a 95% confidence interval means the true answer would be in the confidence interval 95 times, or 90 times for a 90% interval. *Optional*

○ Very Interesting ○ Interesting ○ Neither Interesting or Disinteresting
○ Disinteresting ○ Very Disinteresting

If you would like to, please explain your answer

P-value: The probability that the answer is due to chance when no difference actually exists between the two groups. This value between 0 and 1 tells us how surprised we should be with our answer. A very small p-value means that we would be very surprised if the answer was wrong. Whereas a very large p-value means that we would not be suprised if the answer was wrong. *Optional*

○ Very Interesting ○ Interesting ○ Neither Interesting or Disinteresting
○ Disinteresting ○ Very Disinteresting

If you would like to, please explain your answer

Do you have anything else you would like to add or comment on about how best to show the answer to the question whether a new treatment extends a patient's life? *Optional*

Interest In Further Participation

Thank you for completing this questionnaire.

We wish to keep our work transparent, and open to comment by the public and have the option for you to consent to be contacted about the results of this research throughout its lifetime.

We hope the results of this questionnaire will determine the direction of the research and change how “overall survival” can be assessed in cancer clinical trials. If the questionnaire shows that different groups of people have different views, we will invite members from each group to discuss those views and come to an agreement for the direction of the research.

If you would like to be involved in these discussions, we will invite you to discuss your views. You will need to provide your consent for this.

Are you interested to learn more and have the option to consent to further participation? ✱ *Required*

○ Yes ○ No

Further Participation

Frequently Asked Questions

This section answers some of the questions you may have about continuing to participate in the research.

What will I have to do if I consent to being contacted about the research results?

Less info

If you consent to being contacted you will need to provide your name and email address. This information will not be shared outside the research team. When contacted you may be given the option to comment on the information provided. This is entirely up to you and there will be no expectation on you to provide feedback.

Click "More Info" to find out!

How often will I be contacted about the research's results?

Less info

It is expected that you will be contacted a minimum of 3 times throughout the lifetime of the research to inform you about:

- The results of the questionnaire and discussions, including the direction for the project – Expected Autumn 2022
- The methods which been developed as a result of the stakeholder consultation – Expected Summer 2025
- The final message of the whole research – Expected Summer 2026

However, there may be other times where the research team deem it necessary to feedback information to participants. Such as when information will be presented at conferences or in journals or just how the research is going.

Click "More Info" to find out!

What will I have to do if I consent to being contacted about the discussions?

Less info

If you agree to being contacted you will need to provide your name and email address. This information will not be shared outside of the research team. Once the answers of the questionnaires have been collated and summarised you will be contacted by the Chief Investigator. If you still wish to take part in the meetings you will be asked to complete a questionnaire prior to the meeting and then discuss your views within the meeting. At the end of the meeting, you will be asked to complete the same questionnaire to allow you to reflect on the discussions within the meeting. The results of these questionnaires will be used to direct future discussions and the project direction. The meetings will be arranged for two hours, and pre-meeting activity will be expected to take you no more than an hour. The meetings will likely be virtual using Microsoft TEAMS and will be recorded for note taking. However, the recording will be deleted at the end of the research. If the meeting is not held virtually it will be held in Leeds.

Click "More Info" to find out!

If I consent will I definitely be chosen to take part in the discussions?

Less info

Unfortunately, no. Where possible we will aim to accommodate all individuals who wish to participate further. However, to ensure productive discussions only 12 participants will be invited. The 12 participants will be chosen at random to ensure, where possible, a representative group of people with different age, gender and ethnicity will be invited.

Click "More Info" to find out!

If I consent who will I be in the discussions with?

Less info

There are two possible discussions you can consent to attend:

1. With other members of your group

2. With other members of your group and other groups

Each discussion will also include the Chief Investigator, Kara-Louise Royle, another member of the research team acting as an observer and a patient and public facilitator, if patient and public members are present.

Click "More Info" to find out!

Can I change my mind?

Less info

Yes. Please see the picture below on what to do if you change your mind after agreeing to further participation today.

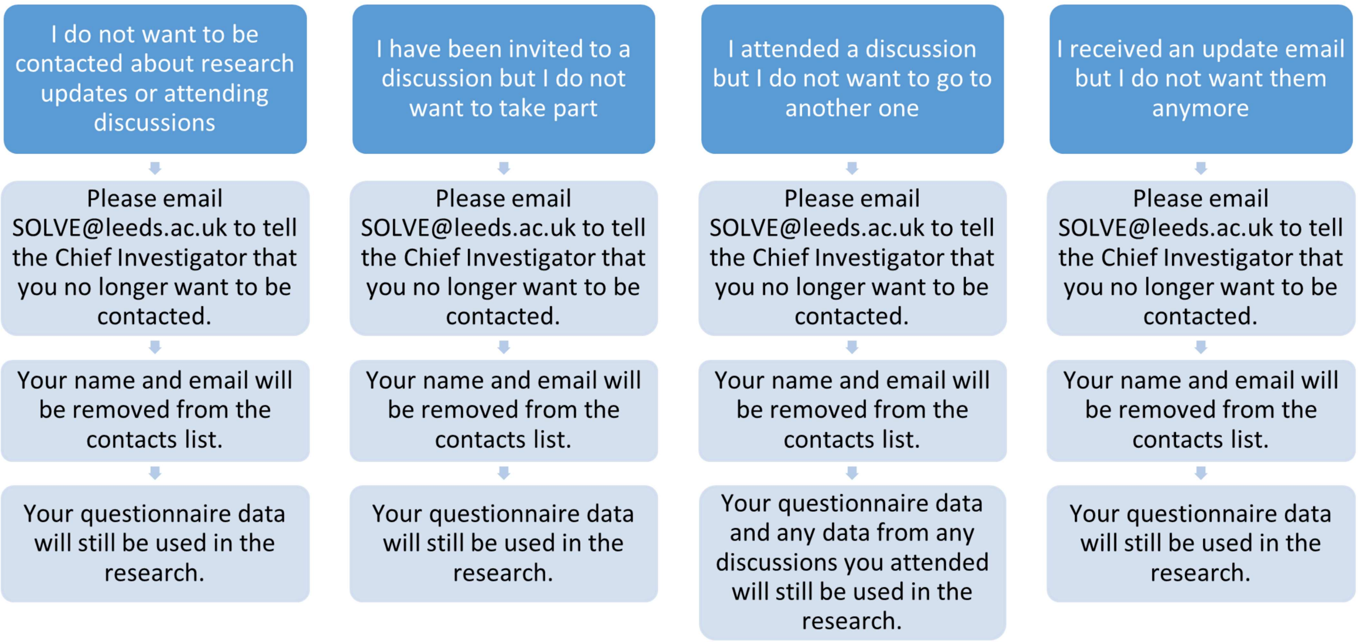

Click on "More Info" to find out!

Will I get any reimbursement or financial incentive for taking part?

Less info

If the discussions are arranged to be face-to-face, you will be reimbursed for you travel expenses. In addition, patient and public participants will be reimbursed for their time to reflect that those coming from a professional capacity will be taking part during their working hours.

Click "More Info" to find out!

How will the additional data be protected?

➖ Less info

The recordings from discussions and your contact details will be stored securely on the online survey database or on University of Leeds network computers with access restricted to the research team. Any quotes from discussions will be presented in summaries, conference presentations and journal articles in such a way that you cannot be identified.

Click "More Info" to find out!

How long will you keep the additional data?

➖ Less info

Recordings of discussions and contact details will be deleted at the end of the research.

Click "More Info" to find out!

Optional Consent

If you are interested in participating in the research further, please tick each option you agree to.

☐ I agree that the Chief Investigator Kara-Louise Royle can contact me to tell me about the results of the research during and at the end of the research and understand what this will involve.

☐ I agree to be contacted for attendance at discussions with other participants in the same group as me and understand what this will involve.

☐ I agree to be contacted for attendance at discussions with other participants in a different group to me and understand what this will involve.

I understand that by providing my contact details my identity will be known and my questionnaire answers will no longer be anonymous.

○ Yes

Please provide the email address you would prefer to be contacted with and your preferred name. By providing this information you are consenting to the electronic storage of personal information provided by yourself which will be only available to the research team for the duration of the research.

I understand that my data from additional questionnaires and discussions, may be looked at by responsible individuals from the research team where it is relevant to my taking part in the research. I give permission for these individuals to have access to my data and know my identity.

○ Yes

I understand that my data from additional questionnaires and discussions will be presented at meetings and in publications relating to the research without me being identified.

○ Yes

I agree to the discussions being recorded. I understand that the recordings will be stored securely, accessed only by the research team, and will be deleted at the end of the research.

○ Yes

End of Questionnaire

Do you have any final comments or feedback on the questionnaire that you would like to add?

Final page

Thank you for taking part in the questionnaire.

Key for selection options

13 - What is your ethnic group?

English/Welsh/Scottish/Northern Irish/British
Irish
Gypsy or Irish Traveller
White and Black Caribbean
White and Black African
White and Asian
African
Caribbean
Arab
Other, please describe
Prefer not to say

15 - Where in the UK are you based?

Scotland
Northern Ireland
Wales
North West England
North East England
Yorkshire and The Humber
East Midlands
West Midlands
London
South East England
South West England
Other
Prefer not to say

17 - Are you? Click on "More Info" for the definitions of each group.

A Member of the Public
A Patient or Carer
A Health Professional
An Industry Partner
A Payer
A Statistician or Other Data Analyst
