# Supplementary material for: How should overall survival be analysed in randomised clinical trials in cancer if participants receive subsequent treatment lines? A stakeholder consultation
Source: Trials. 2025 Oct 24;26:434. doi: 10.1186/s13063-025-09148-3 (PMC12551141; doi:10.1186/s13063-025-09148-3)
Supplement: Supplementary file 4 — Additional File 4: Presentation to stakeholder advisory group following questionnaire analysis. [file 13063_2025_9148_MOESM4_ESM.pdf]

SOLVE

Assessing overall survival – can we do it better?

Stakeholder Advisory Meeting

24th November 2022

ctru University of Leeds

UNIVERSITY OF LEEDS

Meeting Agenda

- Introduction
- Questionnaire Results
- Draft documentation for the discussion group meetings
- AOB

Introduction

Motivation

What are the long-term effects of the experimental intervention?

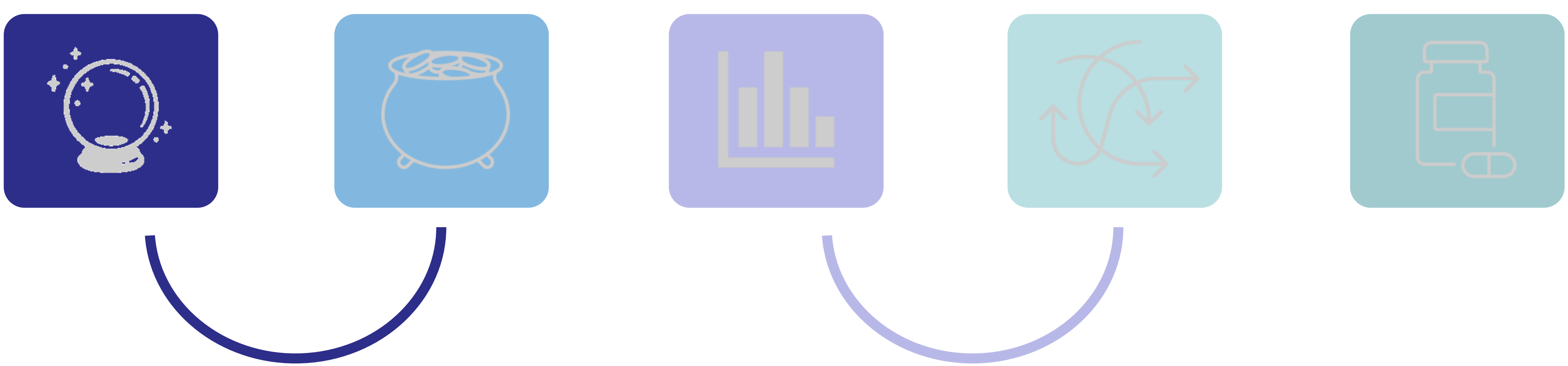

Motivation

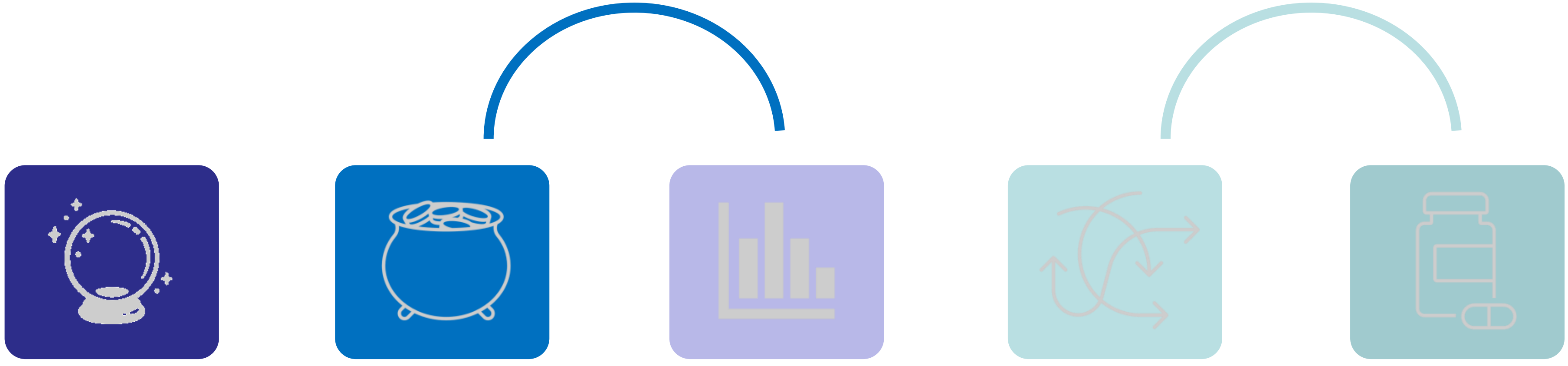

Overall survival is the gold standard endpoint for cancer trials.

Motivation

Current methodology ignores post-trial interventions.

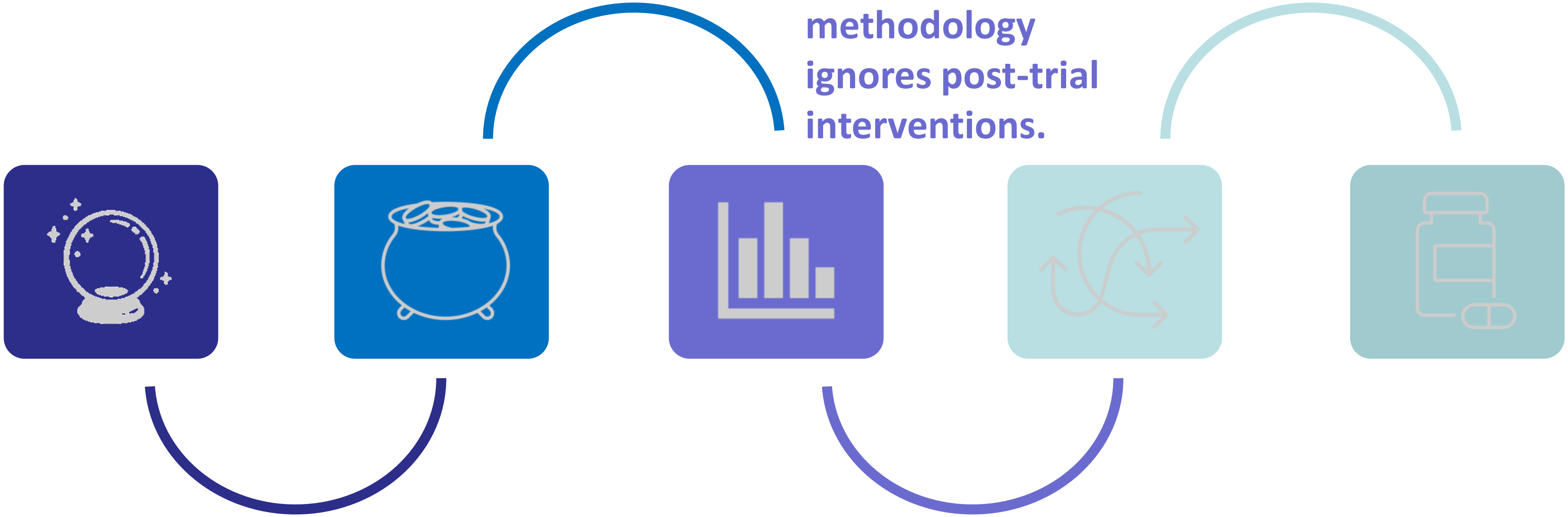

Motivation

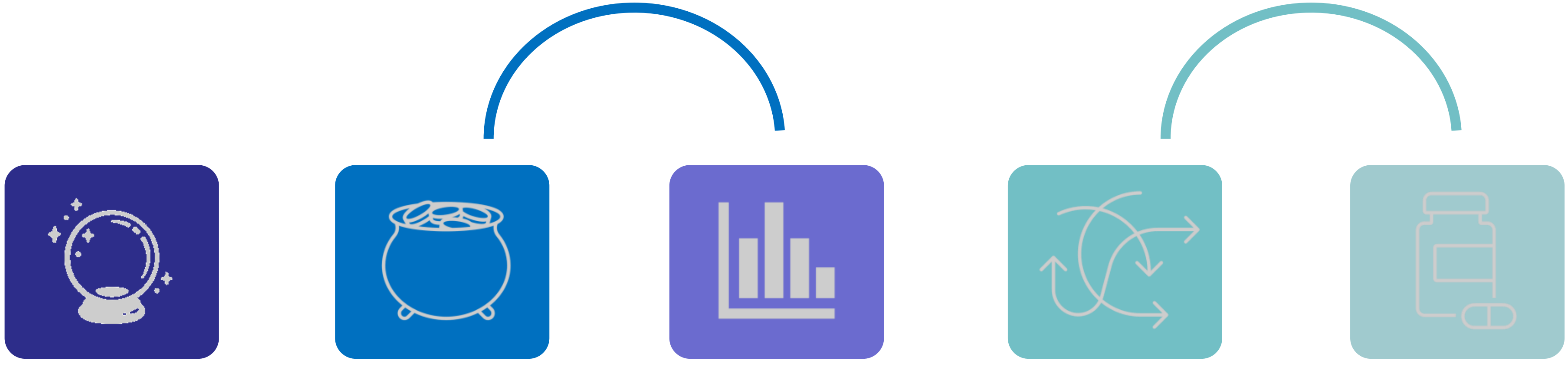

This confuses the evidence available to evaluate the experimental intervention.

Motivation

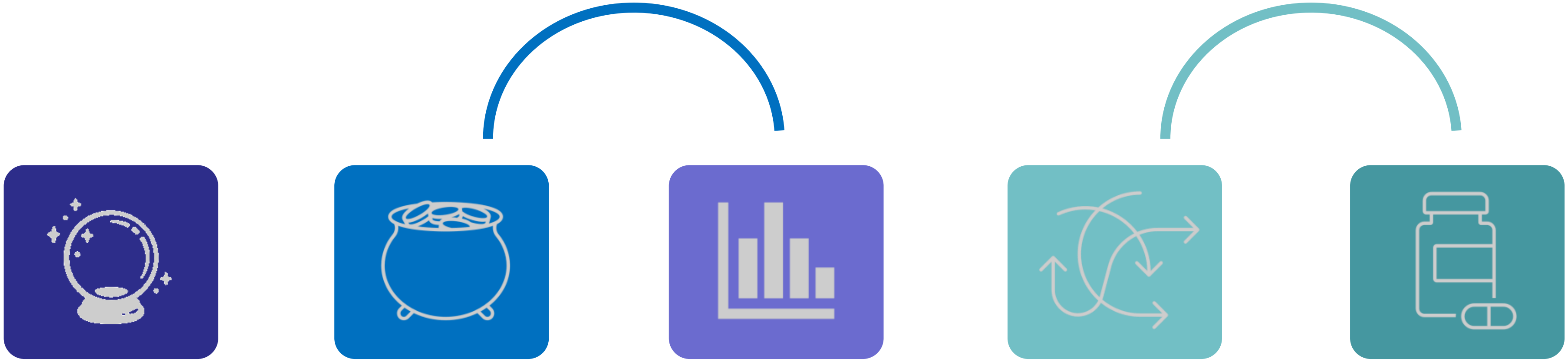

This is a growing concern as more treatments are developed.

?	How should overall survival be assessed in clinical trials of relapsing cancers, given that patients can receive anti-cancer therapy post-trial?
(target)	To develop new or extend existing methodology which assesses overall survival whilst accounting for subsequent lines of anti-cancer therapy.

ctru University of Leeds

SOLVE Assessing overall survival – can we do it better?

UNIVERSITY OF LEEDS

Project Overview

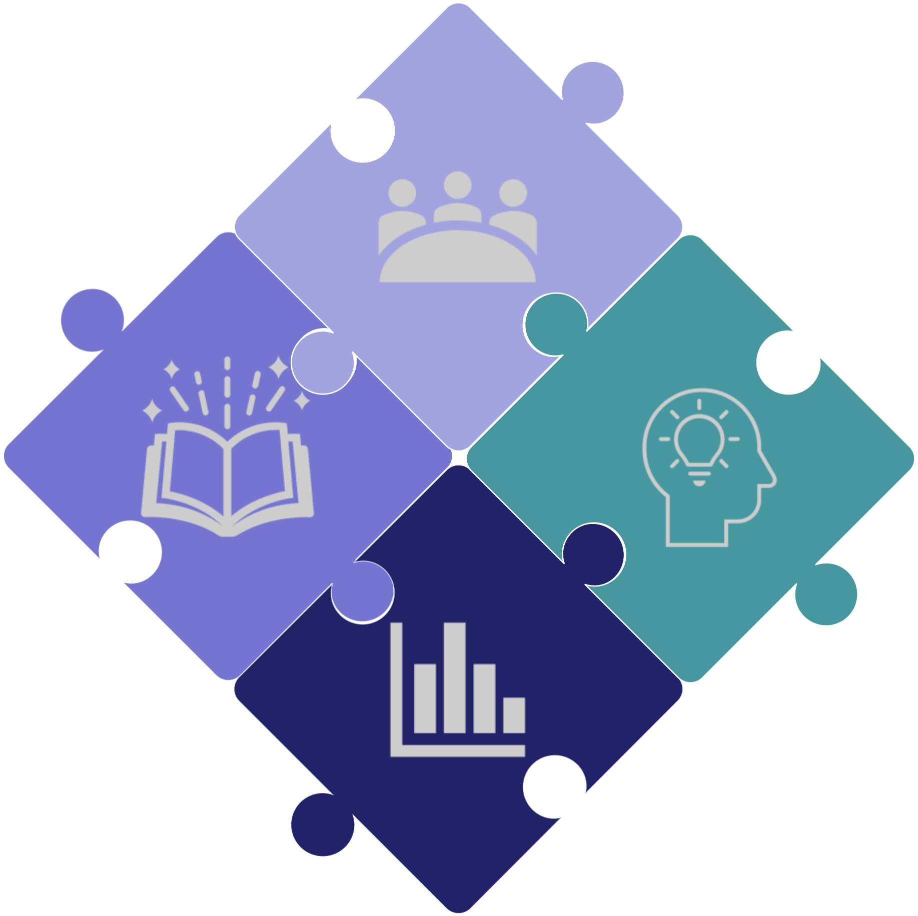

SOLVE
Assessing overall survival – can we do it better?

ctru
University of Leeds

UNIVERSITY OF LEEDS

Project Overview

WP1

Existing Methods and Opinions

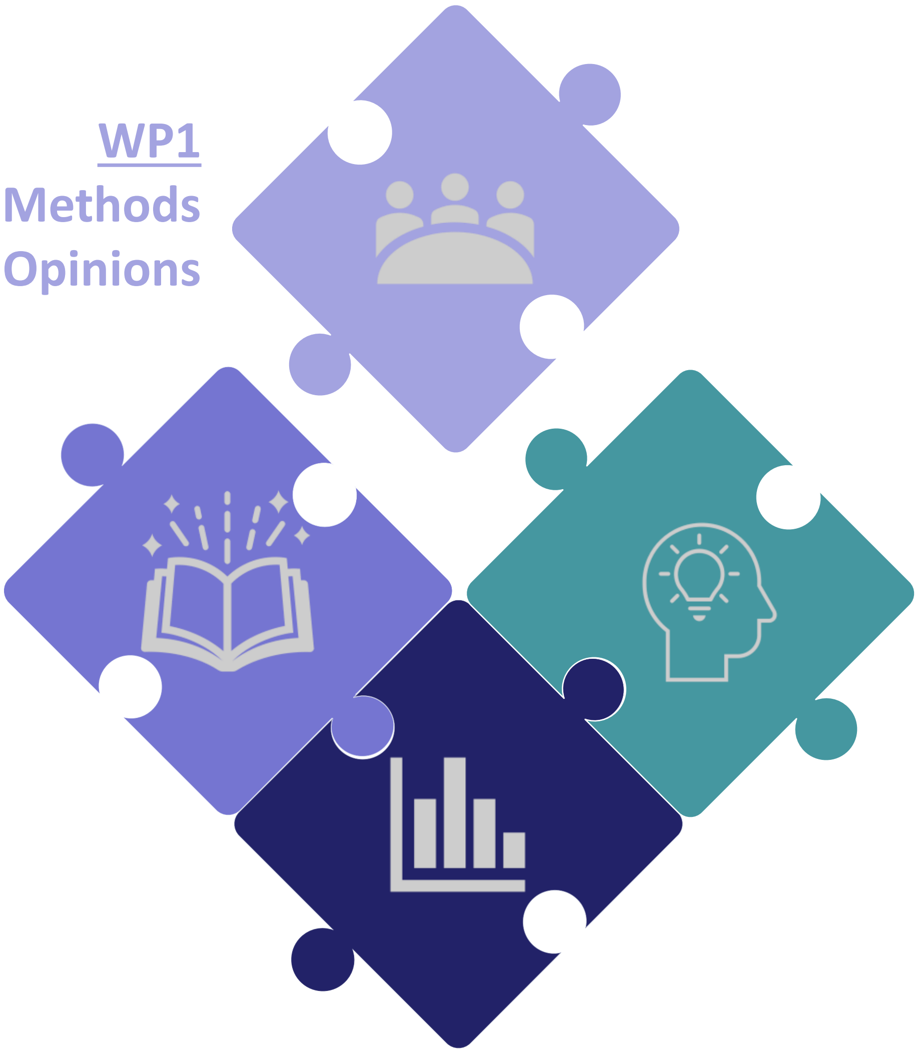

SOLVE
Assessing overall survival – can we do it better?

ctru University of Leeds

UNIVERSITY OF LEEDS

Project Oversight

Stakeholder Advisory Committee

Supervisory Team

Kara-Louise
Royle

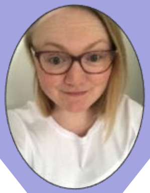

SOLVE
Assessing overall survival – can we do it better?

ctru
University of Leeds

UNIVERSITY OF LEEDS

Questionnaire Results

Demographics

Questionnaire Respondents

103 people completed the questionnaire

61.2% were female

52

The median age was 52

81.6% identified as English / Welsh / Scottish / Northern Irish / British.

40.8% were based in Yorkshire and The Humber

A third found the questionnaire through a mailing list

66% had prior experience of clinical trials.

78.6% thought it was important to consider the effect of post-trial treatment

ctru University of Leeds

SOLVE Assessing overall survival – can we do it better?

UNIVERSITY OF LEEDS

Questionnaire Respondents

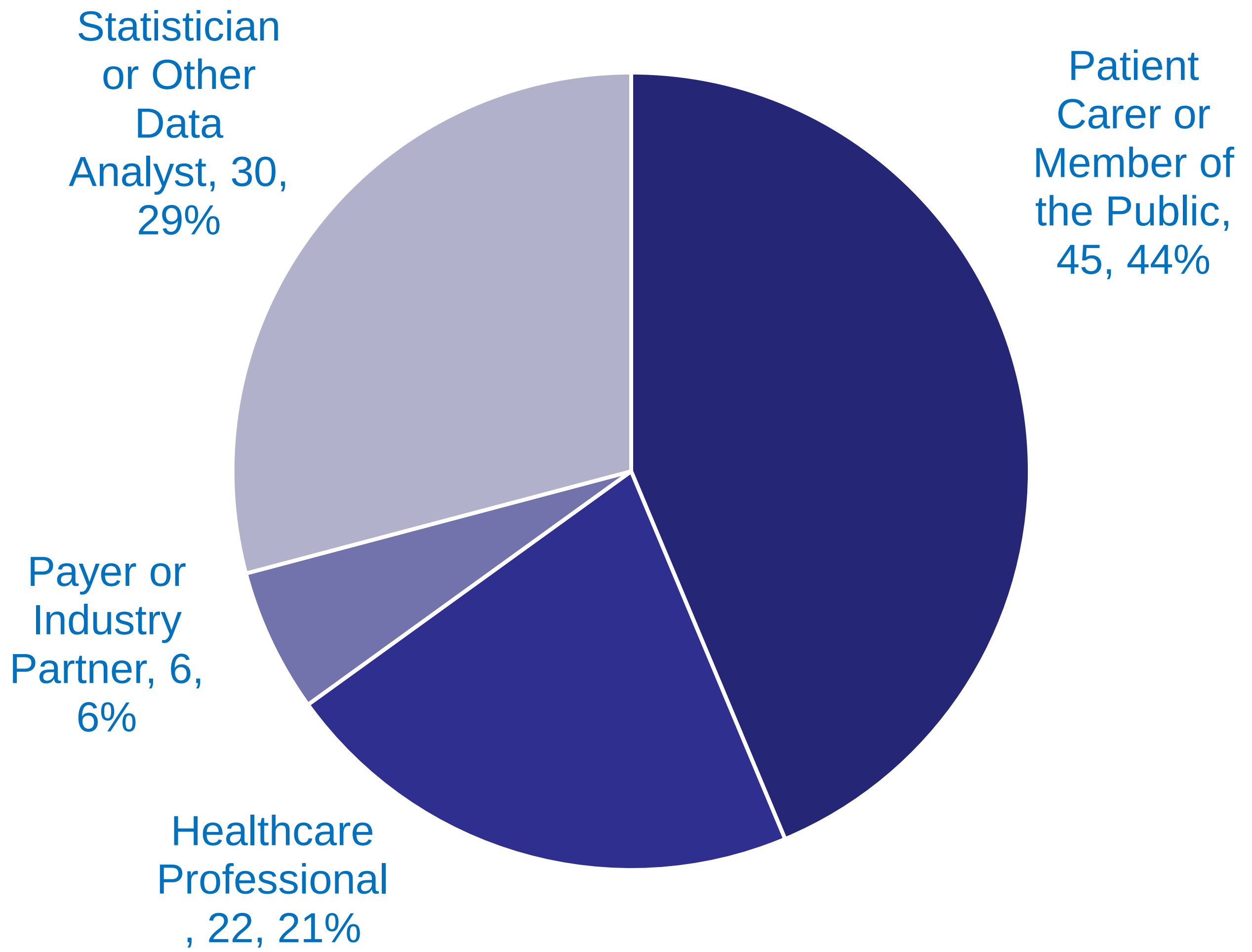

Question of Interest

- The first section of the questionnaire focused on what question we are trying to answer when analysing overall survival.
- Questionnaire respondents were asked to rate four questions from 1 (Most Interested) to 4 (Least interested).
- Prior to analysis the following guidelines were agreed to aid interpretation of the questionnaire results:
    - A question with a median of 1 would be classed as consensus to investigate further.
    - A question with a median of 4 would be classed as consensus to not investigate further.
    - A question with a median of 2, or that had more than a third of respondents in each of the extremes would be classed as uncertain and require further discussion.

How does the new treatment extend survival compared to the control treatment - even though some participants stopped their trial treatment prior to death?

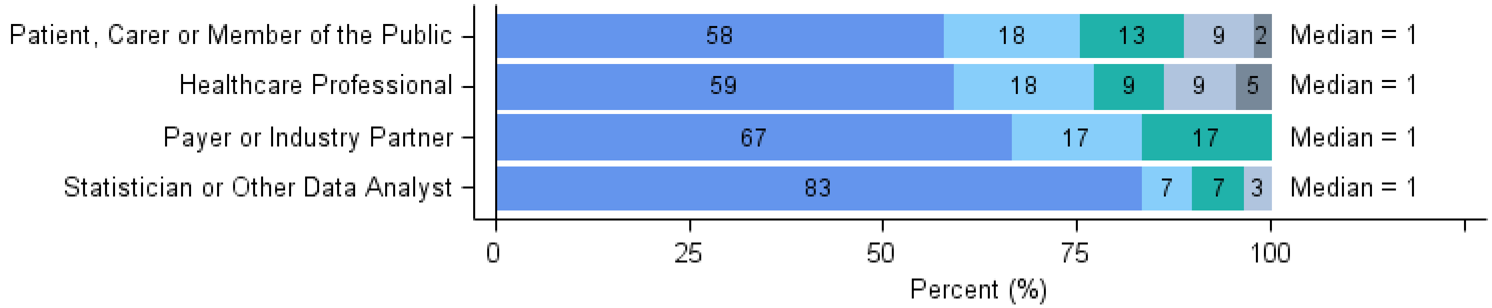

How would the new treatment have extended survival compared to the control treatment - if no one stopped their trial treatment prior to death?

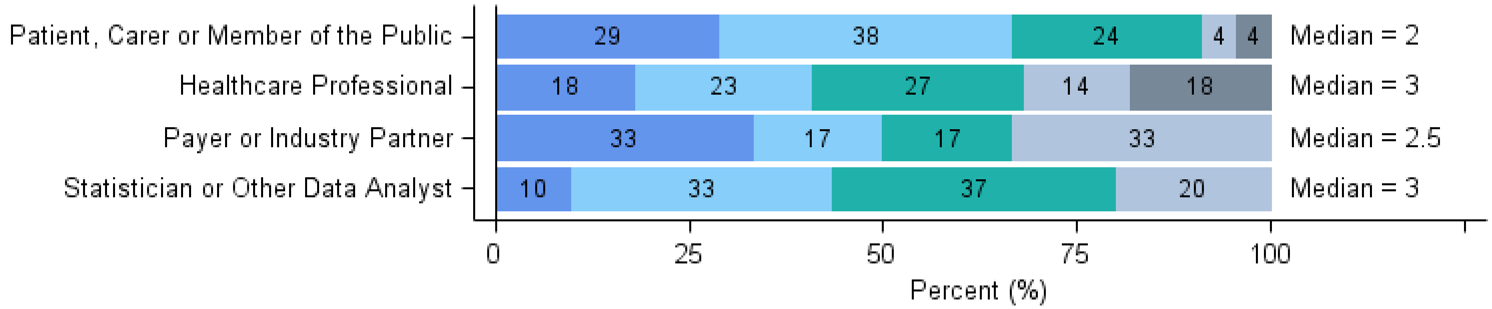

Guidelines
Include - Median = 1
Uncertain - Median = 2 or disagreement
Exclude - Median = 4
Disagreement: ≥ 1/3 in 1 and ≥1/3 in 4

Key

Colour	Score
	1 (Most Interested)
	2
	3
	4 (Least Interested)
	Missing

SOLVE
Assessing overall survival – can we do it better?

ctru University of Leeds

UNIVERSITY OF LEEDS

How does the new treatment extend survival compared to the control treatment - in participants who only received their trial treatment prior to death?

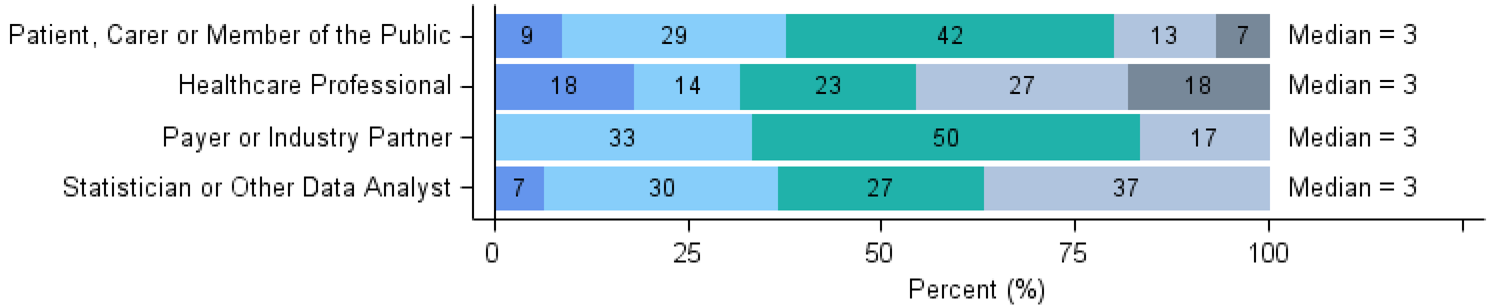

How much longer did participants stay on the experimental treatment compared to the control treatment?

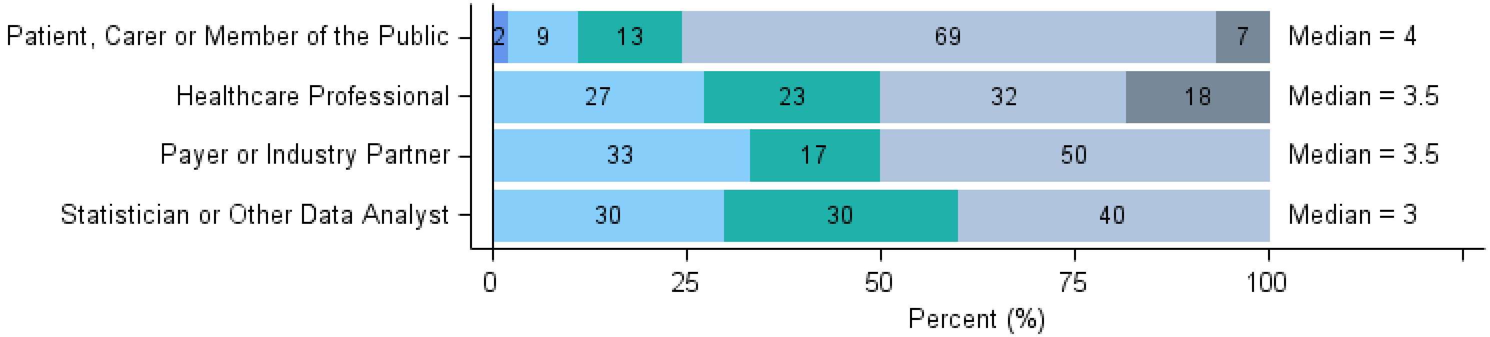

Guidelines
Include - Median = 1
Uncertain - Median = 2 or disagreement
Exclude - Median = 4
Disagreement: ≥ 1/3 in 1 and ≥1/3 in 4

Key

Colour	Score
	1 (Most Interested)
	2
	3
	4 (Least Interested)
	Missing

SOLVE
Assessing overall survival – can we do it better?

ctru University of Leeds

UNIVERSITY OF LEEDS

Free-Text Comments

- Thirty-Three respondents provided scenarios where the question they were most interested in would change. The two main themes were:
  - Other Effects – Explanations where the other effects of the trial treatment are brought into the question such as QoL or side effects.
  - Context – Explanations where the context of the trial, disease area and standard of care options are considered
- Forty-one respondents suggested additional questions, the main themes were:
  - Post-Trial Treatment – Questions about the post-trial treatment trial participants received.
  - Consequences – Questions around the consequences of being in the trial.
  - Other Effects – Questions around the effect of treatment on other outcomes such as QoL or side effects.
  - Patient Characteristics – Questions around how different patient characteristics such as age or comorbidities influence the treatment effect.

My Interpretation

- From the results I think that the question about overall survival that every stakeholder group is interested in, in at least some scenarios is:

“How does the new treatment extend survival compared to the control treatment - even though some participants stopped their trial treatment prior to death?”

- Therefore, I am suggesting that the project should aim to assess overall survival in full, considering participants who stop trial treatment prior to death in a way which does not:
    - Assume no one stopped their trial treatment prior to death,
    - Consider only those who only received their trial treatment prior to death, or
    - Shorten overall survival to be the time on treatment.
- This prioritises the first question and leaves the other three questions as potential extensions if there is time in the project to address them. The other questions around QoL and side effects are outside of the scope of this research but I plan to include a summary of them in my report as a point for future research.

Any Comments?

Information Required

- The next section of the questionnaire asked respondents to score whether they agreed or disagreed with the collection of certain pieces of information on trial participants once they had stopped their trial treatment.
- Questionnaire respondents were asked to score between 1 (Strongly Agree) to 5 (Strongly disagree) as to whether different pieces of information should be collected.
- Prior to analysis the following guidelines were agreed to aid interpretation of the questionnaire results:
  - A data item with a median of 1-2 would be classed as consensus to collect
  - A data item with a median of 4-5 would be classed as consensus to not collect.
  - A data item with a median of 3 or one which had more than a third of respondents in each of the extremes would be classed as uncertain and require further discussion.

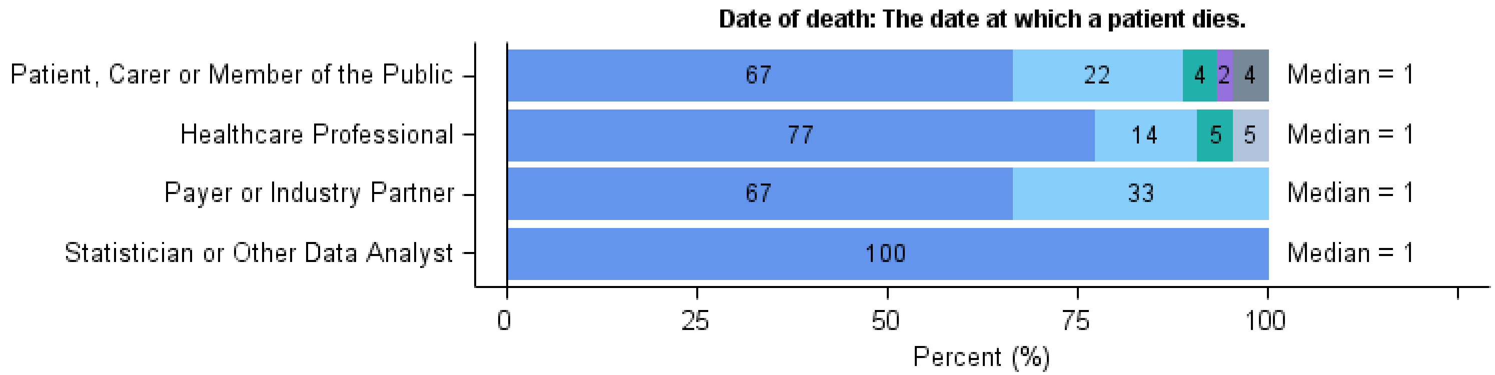

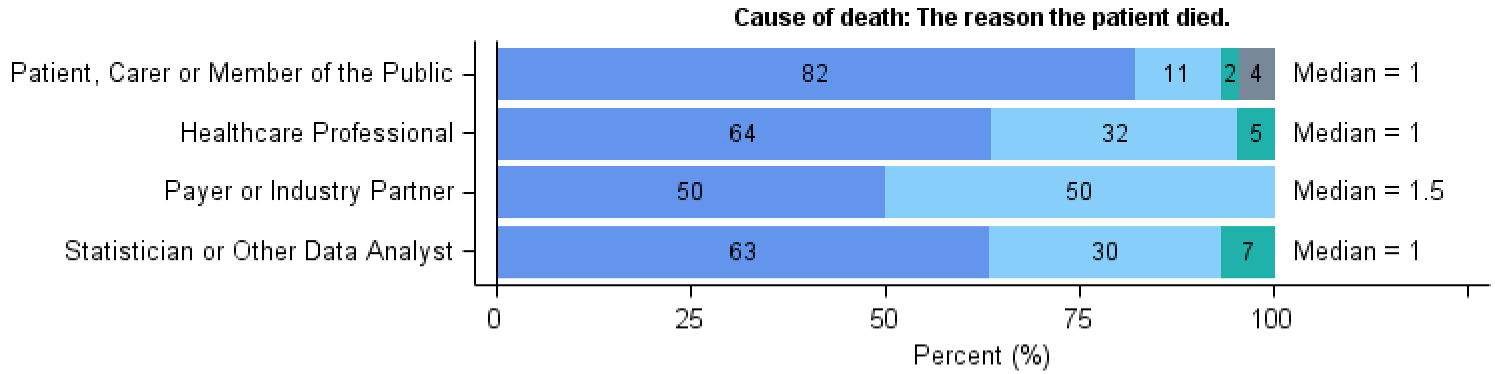

Key	
**Colour**	**Score**
	Strongly Agree
	Agree
	Neither Agree or Disagree
	Disagree
	Strongly Disagree
	Missing

Guidelines
Include - Median = 1-2
Uncertain - Median = 3 or disagreement
Exclude - Median = 5
Disagreement: ≥ 1/3 in 1 and ≥1/3 in 5

Date(s) of progression / relapse: The date the patient's cancer no longer responds to their current treatment

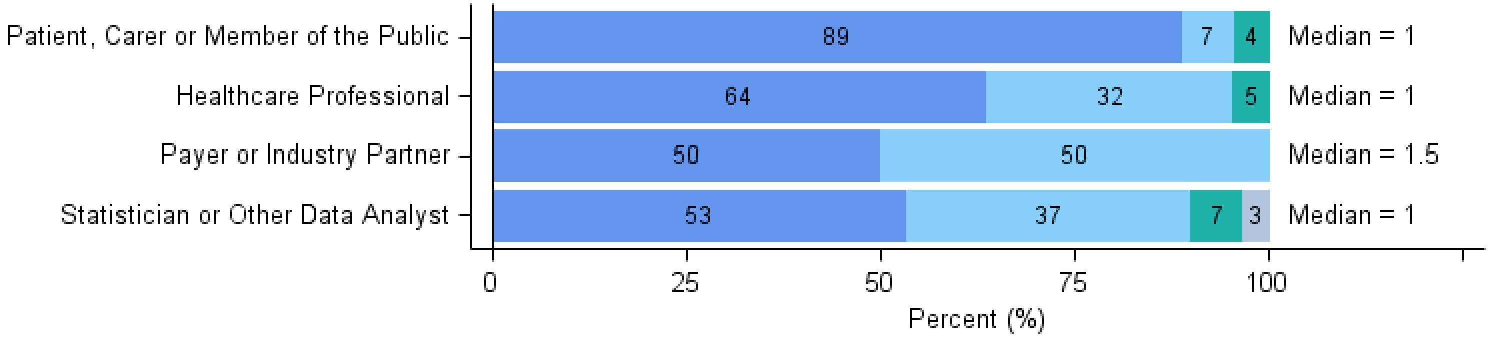

Anti-cancer treatment: The name of any further treatment the patient has to treat their cancer once they stopped their trial treatment and the dates they had it.

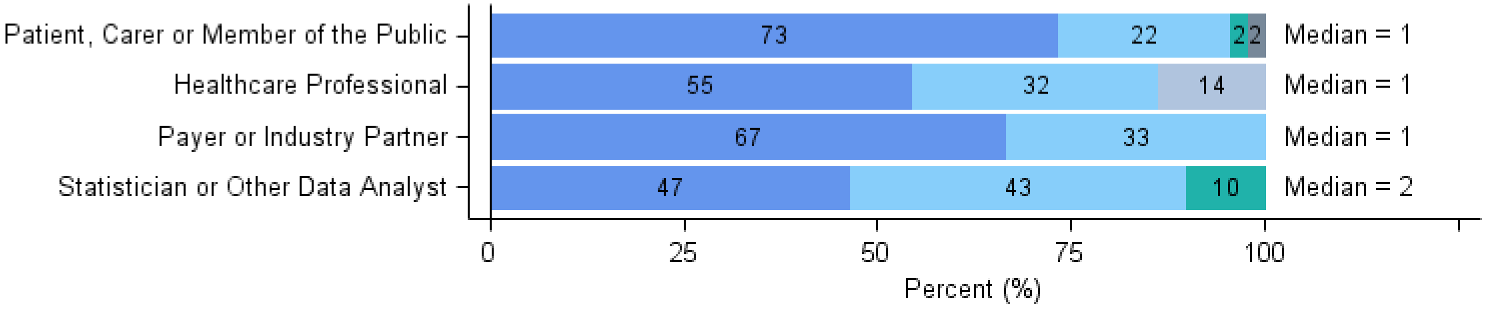

Key

Colour	Score
	Strongly Agree
	Agree
	Neither Agree or Disagree
	Disagree
	Strongly Disagree
	Missing

**Guidelines**
Include - Median = 1-2
Uncertain - Median = 3 or disagreement
Exclude - Median = 5
Disagreement: ≥ 1/3 in 1 and ≥1/3 in 5

SOLVE
Assessing overall survival – can we do it better?

ctru University of Leeds

UNIVERSITY OF LEEDS

Patient Characteristics: Information about the patient such as their height and weight. Measurements which are not necessarily about their cancer.

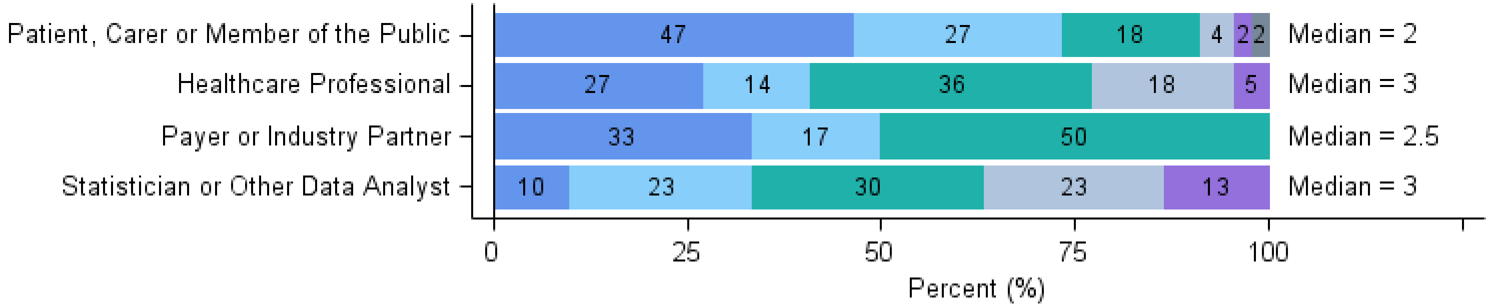

Disease Characteristics: Information about a patient's cancer such as the severity of their cancer.

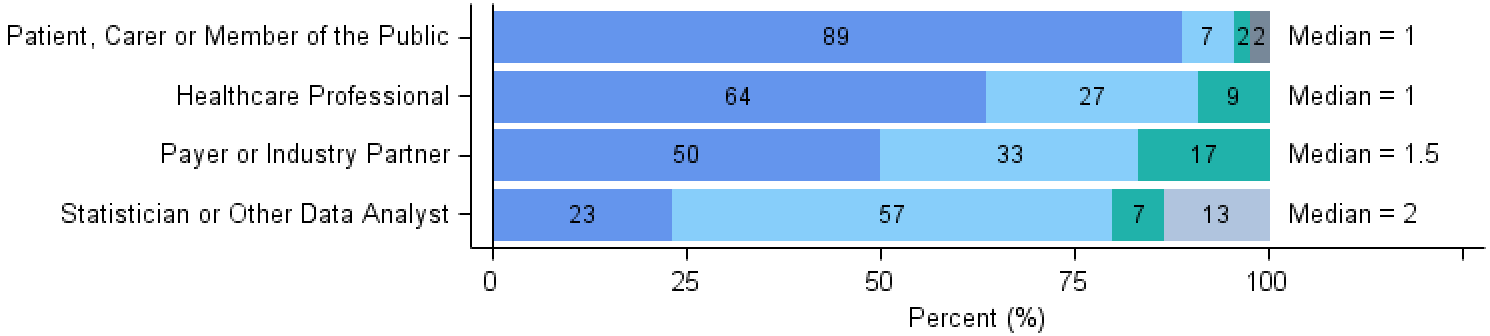

Key	
**Colour**	**Score**
(dark blue)	Strongly Agree
(light blue)	Agree
(teal)	Neither Agree or Disagree
(grey-blue)	Disagree
(purple)	Strongly Disagree
(dark grey)	Missing

**Guidelines**
Include - Median = 1-2
Uncertain - Median = 3 or disagreement
Exclude - Median = 5
Disagreement: ≥ 1/3 in 1 and ≥1/3 in 5

ctru University of Leeds

SOLVE Assessing overall survival – can we do it better?

UNIVERSITY OF LEEDS

Free-Text Comments

- Thirty respondents provided additional comments:
  - Patients, Carers, and Members of the Public focused on how all information could be relevant with regular data collection considered acceptable if pre-specified.
  - Statisticians and Other Data Analysts focused on the practicalities of collecting data long term and making sure what we mean by "progression" is clearly defined.
- Forty-one respondents suggested additional data which could be collected the main types of information were:
  - Quality of Life / Psychological Data
  - Toxicity
  - Co-Morbidities / Concurrent Treatment

My Interpretation

- From the results the majority of data items have a median of 1 or 2 so there is consensus that they should be collected. However, there is a difference of opinion as to whether Patient Characteristics should be collected. I would suggest that the discussion group considers:
    - Should patient characteristics be considered to be collected post-trial treatment? Including co-morbidities and concurrent treatment.
- Along with:
    - Should participation in future trials be collected post-trial treatment?
    - Quality of Life / Psychological Data
    - Toxicity
- These were not included in the original questionnaire.

Any Comments?

Collecting and Recording Information

- The next section asked respondents to rate how they thought information should be:
  - Collected - Whether it should be at a routine appointment or a specific appointment for the trial
  - Recorded – Whether a specific trial database or routine data sources should be used
- Prior to the analysis it was agreed that the one with the highest percentage of participants would be considered for further discussion.

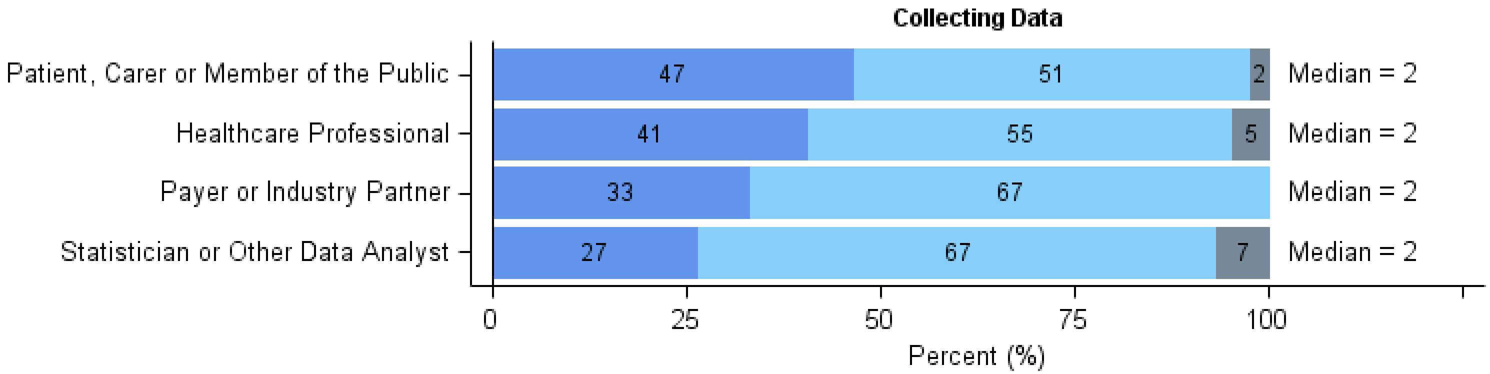

Key	
**Colour**	**Score**
[dark blue]	At a trial follow-up appointment. This appointment could be in person or over the phone and would be an additional appointment to the ones the patient was attending as part of their off-trial treatment.
[light blue]	At a routine appointment. This appointment could be in person or over the phone and would be an appointment which the patient attended as part of their off-trial treatment.
[grey]	Missing

SOLVE
Assessing overall survival – can we do it better?

ctru University of Leeds

UNIVERSITY OF LEEDS

Free-Text Comments: Collecting Data

Main Reasons Provided in Favour of a Trial Follow-up Appointment

- Trial Focus - The participants' explanation mentions that a trial follow-up appointment is conducted by the research team so will keep focus on trial and ensure data collection is completed.
- Phone preference - The participants' explanation mentions that a phone appointment is preferable to a face-to-face appointment to reduce burden on patient.
- Personal Experience - The participants' explanation includes reference to their own opinion and experience of trials.

Main Reasons Provided in Favour of using a Routine Follow-up Appointment

- Patient Burden - The participants' explanation includes reducing the burden and requirement on patients to attend hospital visits in order to improve compliance.
- Context - The participants' explanation notes that the regularity of follow-up appointments would influence their decision on a trial-by-trial basis.
- Logistics - The participants' explanation includes a consideration of how this would work in practice.

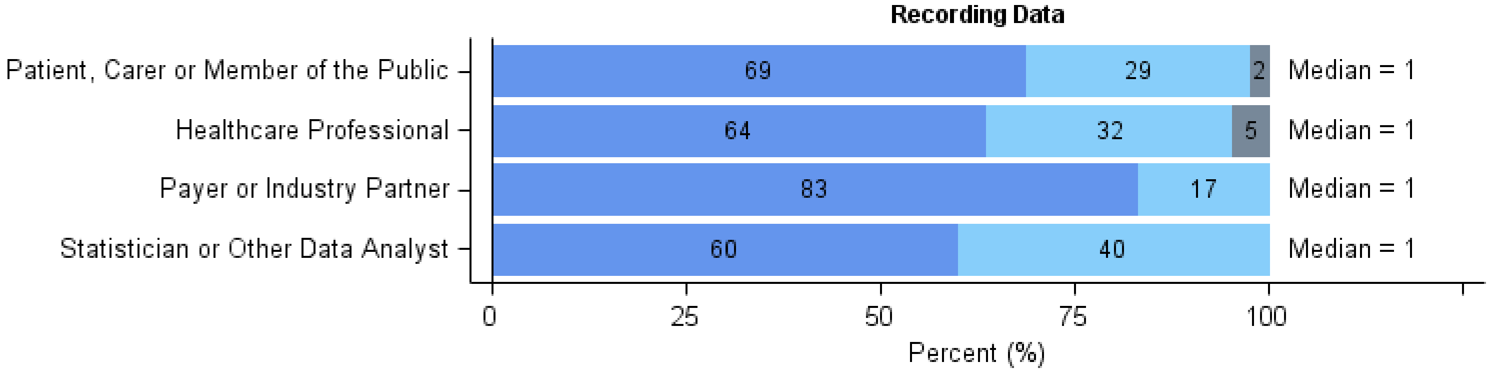

Key	
**Colour**	**Score**
	From a database which was made specifically for the trial. This makes sure everything is recorded in the same way.
	From a database which is completed normally as part of standard practice. This means that information is not duplicated across databases
	Missing

Free-Text Comments: Recording Data

Main Reasons Provided in Favour of a Trial Database

- Standardised Data Collection - The reasoning includes that the required data is collected reliably and consistently in a controlled way to allow for it to be as accurate as possible to ensure a robust analysis.
- Routine Data Access - The reasoning includes a comment around the barriers to accessing data from routine data sources.

Main Reasons Provided in Favour of a Standard Database

- Site burden - The participants' explanation includes a comment about reducing the pressure on site staff to input data.
- Duplication - The participants' explanation includes a comment around reducing duplication across databases.
- Data Sharing - The participants' explanation favours data sharing between general practice and researchers.

My Interpretation

There is a difference of opinion of how this data should be recorded and collected. Therefore, I would suggest that the discussion groups consider the reasons provided for each opinion and discuss:

- How information on trial participants once they have completed their trial treatment should be collected and recorded based on what is currently feasible.
- What the research community should work towards in terms of collecting and recording information on participants who have stopped their trial treatment.

Any Comments?

Assumptions

- The next section of the questionnaire asked respondents to score whether they thought some of the commonly used statistical assumptions held in all scenarios.
- Questionnaire respondents were asked to score between 1 (All Scenarios) to 3 (No Scenarios).
- Prior to analysis the following guidelines were agreed to aid interpretation of the questionnaire results:
    - An assumption with a median of 1 would be classed as consensus to use.
    - An assumption with a median of 3 would be classed as consensus to not use.
    - An assumption with a median of 2 or one which had more than a third of respondents in each of the extremes or 50% in the “unsure” category would be classed as uncertain and require further discussion.

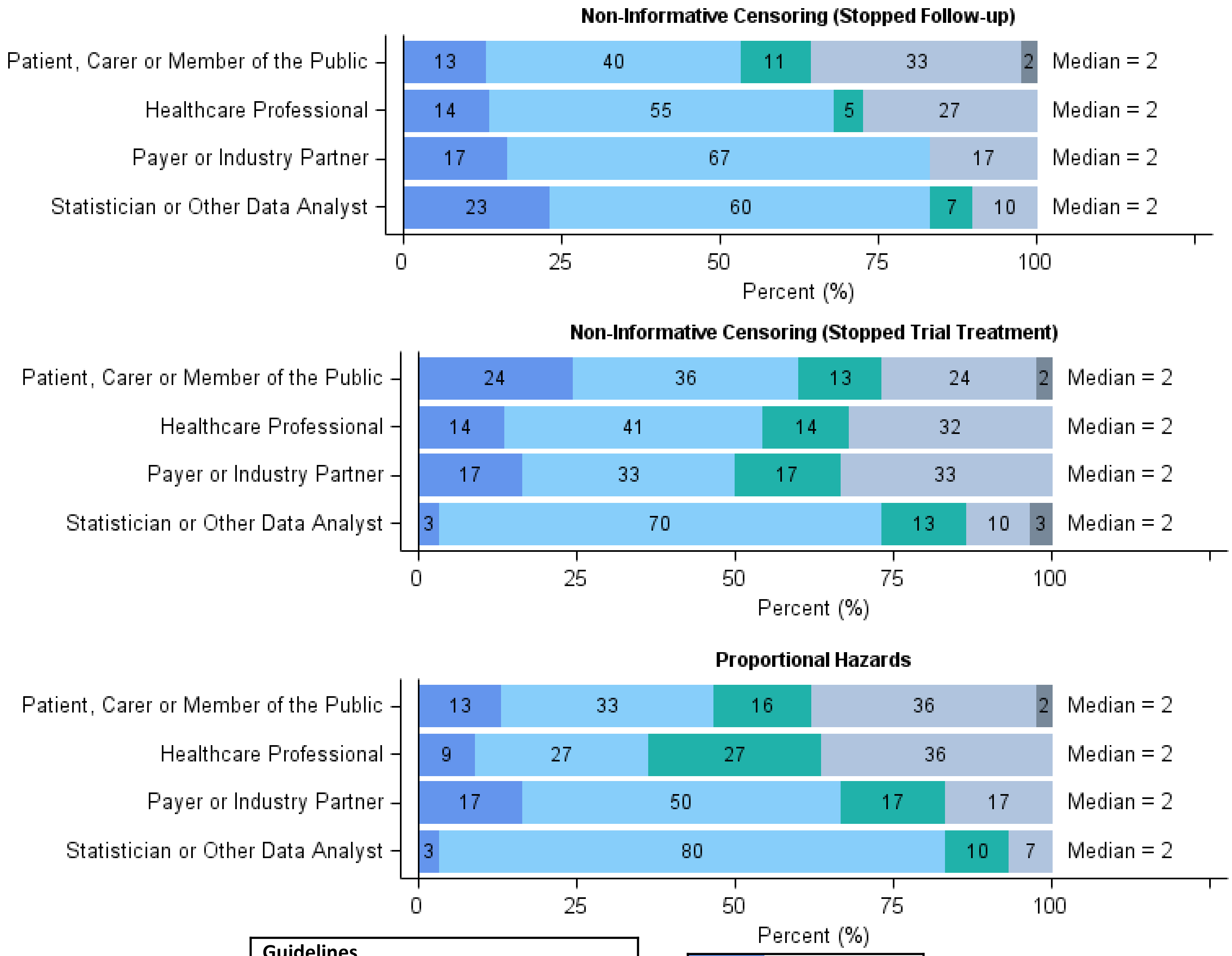

Guidelines
Include - Median = 1
Uncertain - Median = 2 or disagreement
Exclude - Median = 3
Disagreement: ≥ 1/3 in 1 and ≥1/3 in 3
OR 50% in unsure

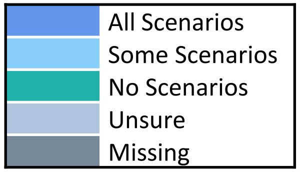

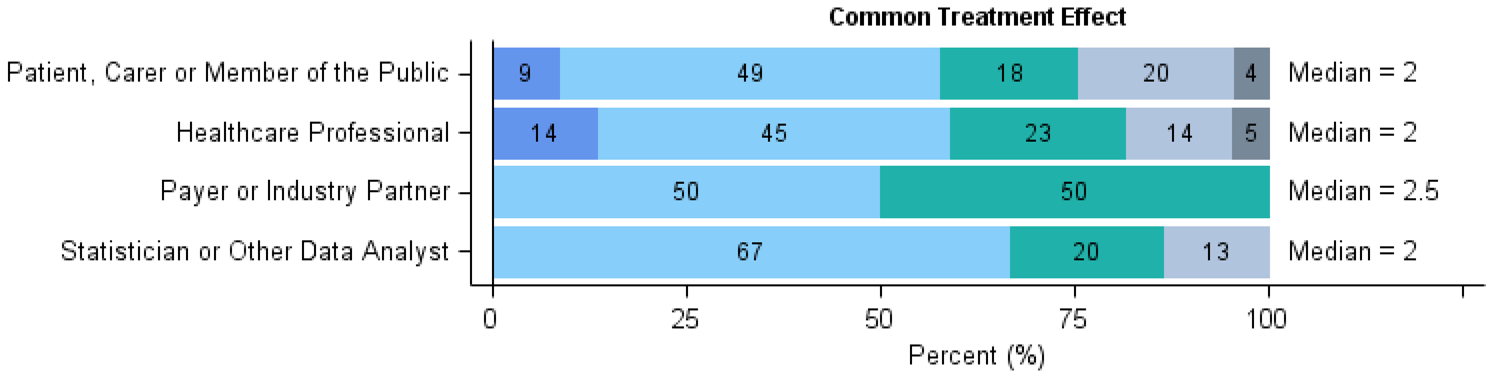

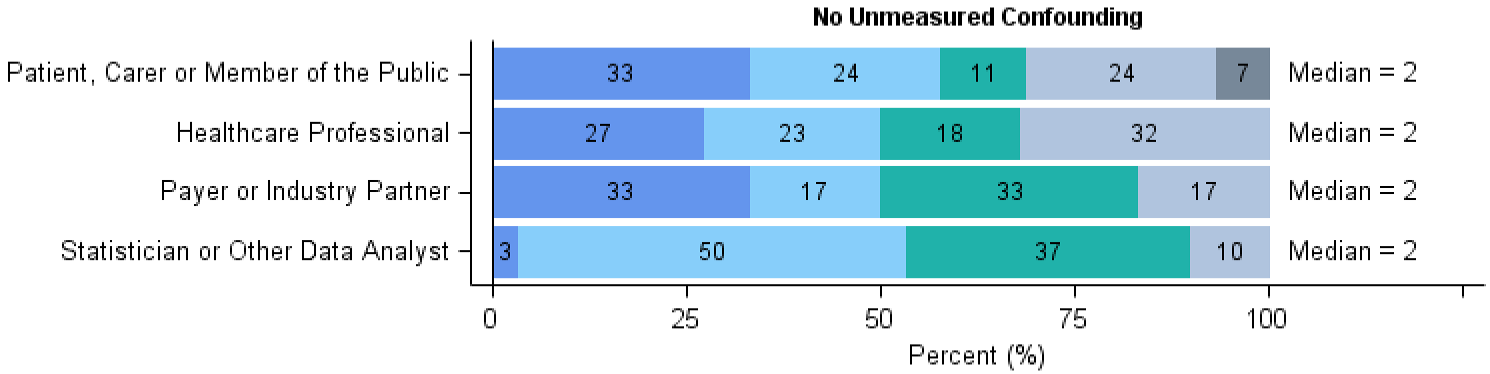

Guidelines

Include - Median = 1
Uncertain - Median = 2 or disagreement
Exclude - Median = 3
Disagreement: ≥ 1/3 in 1 and ≥1/3 in 3
OR 50% in unsure

All Scenarios
Some Scenarios
No Scenarios
Unsure
Missing

UNIVERSITY OF LEEDS

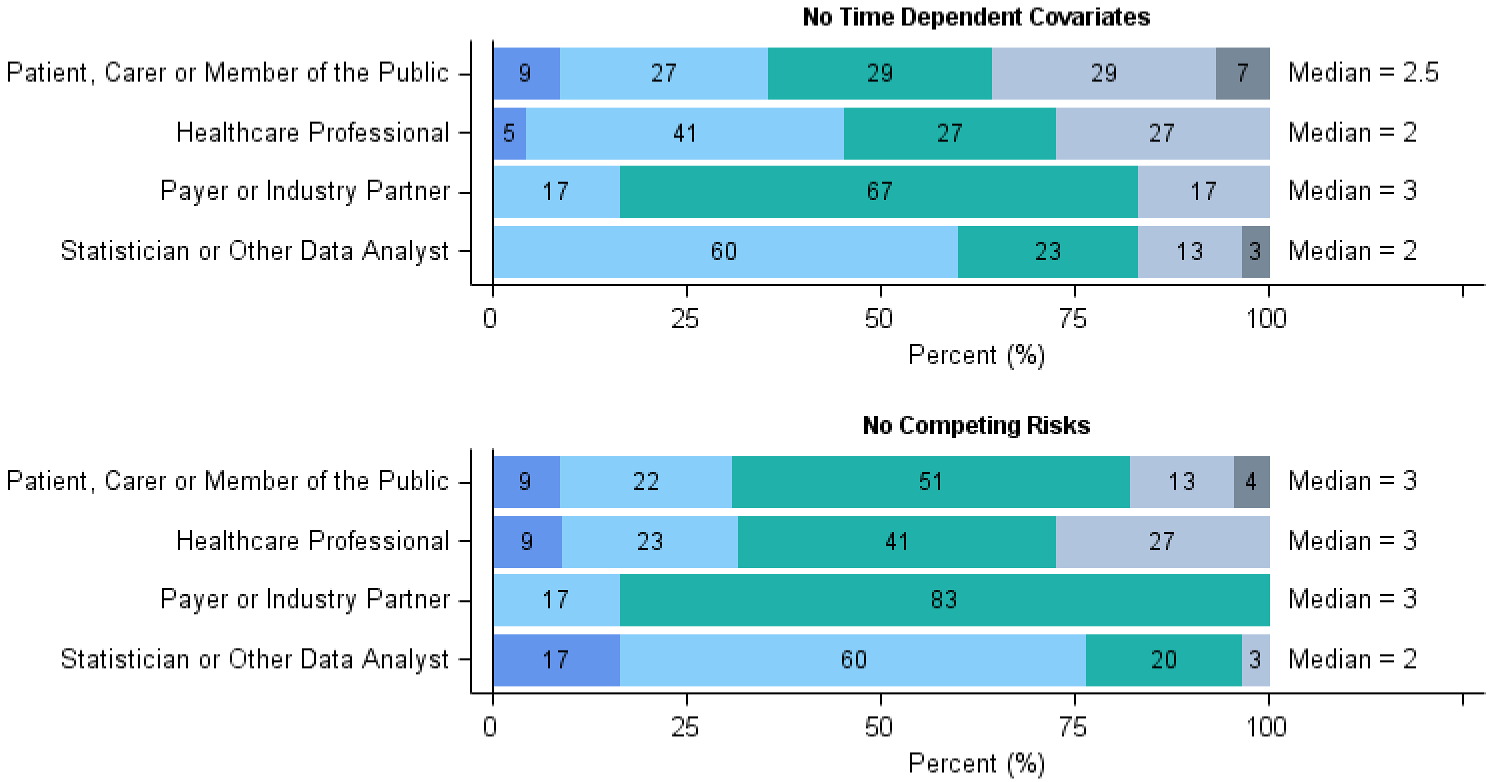

Guidelines
Include - Median = 1
Uncertain - Median = 2 or disagreement
Exclude - Median = 3
Disagreement: ≥ 1/3 in 1 and ≥1/3 in 3 OR 50% in unsure

- All Scenarios
- Some Scenarios
- No Scenarios
- Unsure
- Missing

ctru University of Leeds

UNIVERSITY OF LEEDS

Pause

- From the plots we can see that nearly every assumption is scored as being applicable in “Some Scenarios” this was re-iterated through the free-text comments.

Free-Text Comments & Suggestion

Non-Informative Censoring (Lost-To-Follow-up)	• Context – The explanation given focuses on the context of why participants were lost to follow-up or how it differed between the arms.<br>• Robustness – The explanation has a negative view of assumptions in terms of how reliable the trial results are.
Non-Informative Censoring (Stopped Trial Treatment)	• Reason – The explanation given focuses on the reason for stopping treatment.<br>• Duration – The explanation given focuses on the duration of treatment and how that will affect response.<br>• Future – The explanation given focuses on what happens after stopping treatment and how that would affect survival.

Free-Text Comments & Suggestion

Proportional hazards	• Scenarios – The explanation given provides examples when the assumption will or will not hold.<br>• Test – The explanation given focuses on the testing of the assumption.
Common Treatment Effect	• Disease Burden – The explanation focuses on how the stage of disease may affect how effective a treatment is.<br>• Timing – The explanation focuses on how the timing of a treatment may affect how effective it is.

Free-Text Comments & Suggestion

No Unmeasured Confounding	• Data Collection – The explanation given focuses on how data collection will determine whether this assumption is appropriate.<br>• Misunderstanding – The explanation given suggests that the participant did not understand the question.
No Time Dependent Co-variates	• Factors – The explanation given identifies factors which could change over time.<br>• Relevance – The explanation given focuses on how relevant considering time dependent covariates are and whether they would improve the analysis.
No Competing Risks	• Definition – The explanation given questions whether we are analysing disease-specific survival or overall survival.<br>• Context – The explanation given focuses on the context of the disease / trial in question.

My Interpretation

- From the results it is clear that the respondents understood that assumptions will not be applicable in every scenario. However, there are some which they preferred compared to others from the free-text comments. From this I would like to suggest that these are not discussed further and in the development of the new methodology:
- We are clear about the assumptions we have to make and ensure they can be tested.
- We are cautious about using:
  - Non-Informative Censoring (Stopped Trial Treatment)
  - Proportional Hazards
  - Common Treatment Effect
- and ensure that if assumed, the impact of No Unmeasured Confounding on model performance is assessed.

Any Comments?

Presentation of Results

- The last section of the questionnaire asked respondents to score whether different ways to present the results helped them to understand the answer.
- Questionnaire respondents were asked to score between 1 (Very Helpful) to 5 (Very Unhelpful).
- Prior to analysis the following guidelines were agreed to aid interpretation of the questionnaire results:
    - A presentation method with a median of 1-2 would be classed as consensus to use.
    - A presentation method with a median of 4-5 would be classed as consensus to not use.
    - An assumption with a median of 3 or one which had more than a third of respondents in each of the would be classed as uncertain and require further discussion.

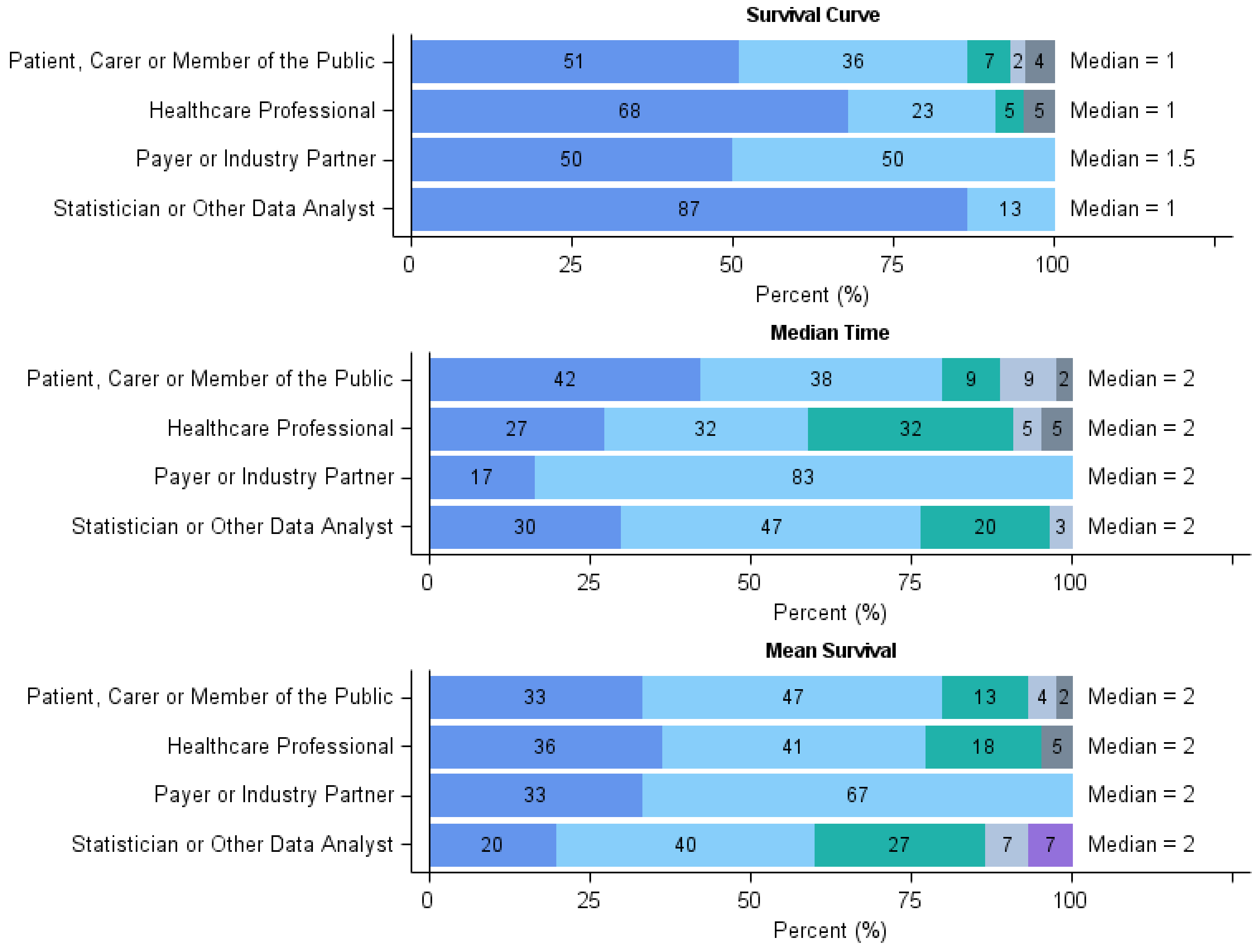

- Very Helpful
- Helpful
- Neither Helpful or Unhelpful
- Unhelpful
- Very Unhelpful
- Missing

Guidelines
Include - Median = 1-2
Uncertain - Median = 3 or disagreement
Exclude - Median = 4-5
Disagreement: ≥ 1/3 in 1 and ≥1/3 in 5

ctru University of Leeds

UNIVERSITY OF LEEDS

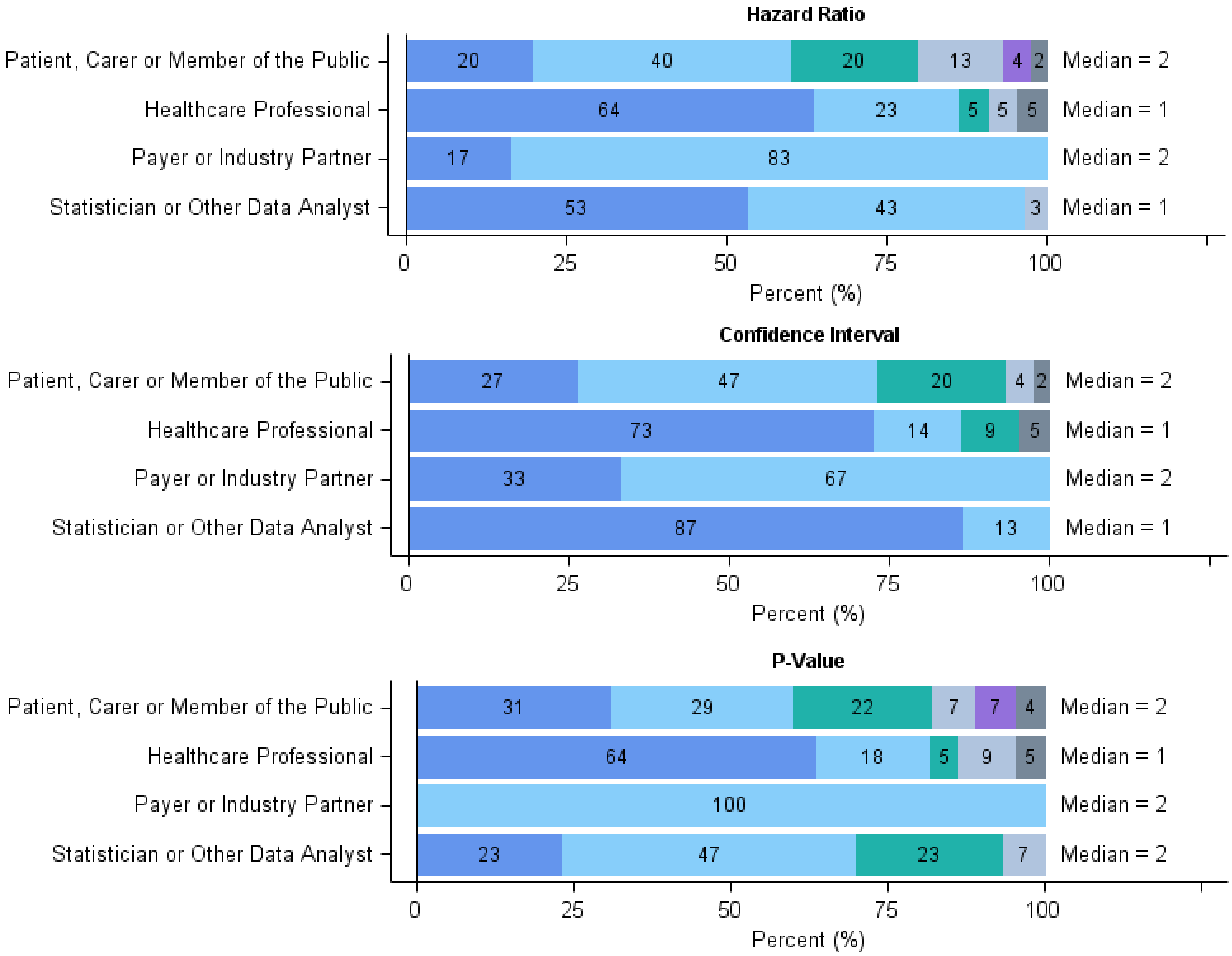

- Very Helpful
- Helpful
- Neither Helpful or Unhelpful
- Unhelpful
- Very Unhelpful
- Missing

Guidelines

Include - Median = 1-2

Uncertain - Median = 3 or disagreement

Exclude - Median = 4-5

Disagreement: ≥ 1/3 in 1 and ≥1/3 in 5

ctru University of Leeds

UNIVERSITY OF LEEDS

Free-Text Comments

There were no obvious themes from the respondents, some key comments from Patients, Carers and the Public were:

- A preference for visual representation of the results
- A preference for a median over a mean
- A concern over the use of the term “Hazard”
- An interest seeing everything, provided that it is well explained
- A need for consistency across studies

The other stakeholder groups commented:

- The best way to present the data will depend on the disease and treatment in question
- Mean Survival is important but comes with limitations

My Interpretation

- From these results each stakeholder group scored each way of presenting the results with a median of “Very Helpful” or “Helpful” from this I would like to suggest that this is not discussed any further and say the approach we develop to tackling the issue of subsequent treatments will look to have a visual representation, a summary statistic in terms of a median and then a hazard ratio which will be supported by at least a confidence interval.

Any Comments?

Summary – Next Steps

Points for agreement

1) Do you agree with the decision for the research to consider the question:

   "How does the new treatment extend survival compared to the control treatment - even though some participants stopped their trial treatment prior to death?"

2) Do you agree with the decision for the research to focus on avoiding using:

   a. Non-Informative Censoring (Stopped Trial Treatment)

   b. Proportional Hazards

   c. Common Treatment Effect

3) Do you agree with the decision that the research should aim to present the results of the methodology using:

   a. A visual representation

   b. A summary statistic in terms of a median

   c. A hazard ratio supported by at least a confidence interval. Where the language around the hazard ratio is considered when fed back to patients and the public.

Points for discussion

1) It is practical and appropriate to collect patient characteristics, participation in future trials, QoL/Psychological data and toxicity once a trial participant has stopped their trial treatment?

2) How should data be recorded and collected during trial follow-up

- Given what is currently possible
- In an ideal world

Stakeholder Discussion Meeting Format

SOLVE
Assessing overall survival – can we do it better?

ctru
University of Leeds

UNIVERSITY OF LEEDS

Stakeholder Discussion Document Comments

ctru
University of Leeds

SOLVE
Assessing overall survival – can we do it better?

UNIVERSITY OF LEEDS

Any Other Business?
