# Supplementary material for: How should overall survival be analysed in randomised clinical trials in cancer if participants receive subsequent treatment lines? A stakeholder consultation
Source: Trials. 2025 Oct 24;26:434. doi: 10.1186/s13063-025-09148-3 (PMC12551141; doi:10.1186/s13063-025-09148-3)
Supplement: Supplementary file 5 — Additional File 5: Pre-focus group material. [file 13063_2025_9148_MOESM5_ESM.pdf]

SOLVE Study – Discussion Group Meeting

Thank you for completing the SOLVE study questionnaire and agreeing to be contacted for participation in the discussion groups.

If you would like to refresh your memory on what the SOLVE study is investigating, you can find an overview of the entire project on YouTube here: https://youtu.be/dZsle9e-NpI. A second video explaining each of the four parts of the project in more detail is here: https://youtu.be/AdfS1FeBhBs.

In total 103 people completed the questionnaire. Their answers were analysed, and the results discussed with representatives from each stakeholder group. This document provides an overview of the results along with some suggestions on how we would like to use the results to determine the next stages of the project and recommendations for future research. We would like to get your opinion on these suggestions in the Discussion Group Meeting on the 24th March 2023.

The meeting will be held on Microsoft TEAMS. It will start at 9:30 and close by 12:30. The plan for the meeting is at the end of this document. The meeting will include members from each stakeholder group along with the lead researcher (Kara-Louise Royle) and one of the studies Patient and Public Representatives. A maximum of 14 people will be in the meeting.

We are using TEAMS, as NHS computers, which some stakeholders will be joining from, cannot access Zoom. It is also the virtual meeting software which the meeting chair is more familiar with. Kara-Louise will join the meeting 15 minutes early to allow you log-in and make sure everything is set-up and working prior to the meeting starting. Joining early is optional. You can join the meeting using this link:

[redacted]

As a reminder the meeting audio and video will be recorded to aid note taking. This recording will not be made publicly available. There will also be the opportunity to provide feedback within the meeting using Padlet. The Padlet boards have been set-up so that only those with the link can access them and access will be removed after the meeting. If you would like to try using Padlet before the meeting please use this link: [redacted]

You do not need an account to access Padlet or Microsoft TEAMS.

The aim of the Discussion Group Meeting is to agree how the SOLVE study should move forward in adapting statistical methodology to account for post-trial treatment when assessing overall survival.

Not everything in this document will be discussed in the meeting. This is made clear throughout the document. If there are any results you would like to raise for additional discussion there will be time at the end of the meeting.

I look forward to meeting and discussing the next steps of the project with you.

If you have any questions or would like a printed copy of this material to be posted to you prior to the meeting please email Kara-Louise Royle at SOLVE@Leeds.ac.uk

Question of Interest

The first part of the questionnaire asked you to consider what question you would be most interested in finding out about overall survival from a clinical trial where the people in the trial may have stopped treatment prior to death. The table below shows the score for each question by each stakeholder group. The key for the plots is at the bottom of the page.

Question	Plain English	Scoring by Stakeholder Group
How does the new treatment extend survival compared to the control treatment - even though some participants stopped their trial treatment prior to death?	In the clinical trial did the new treatment extend patient's lives even though they did not receive it forever?	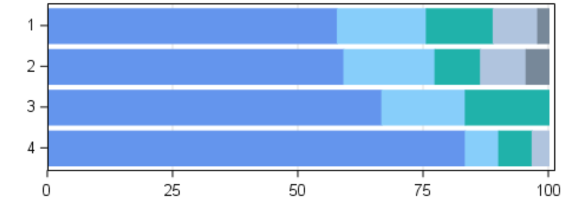
How would the new treatment have extended survival compared to the control treatment, if no one stopped their trial treatment prior to death?	In the clinical trial if everyone had stayed on their trial treatment, would the new treatment have extended patient's lives?	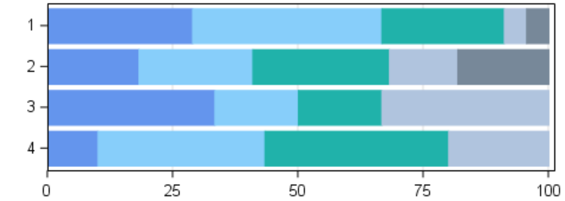
How does the new treatment extend survival compared to the control treatment, in participants who only received their trial treatment prior to death?	In the clinical trial did the new treatment extend patient's lives if they only received their trial treatment?	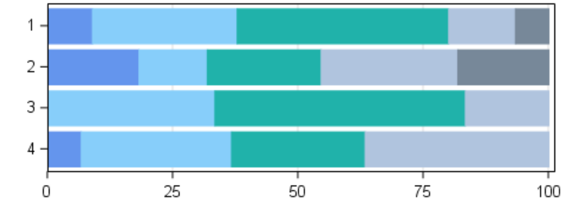
How much longer did participants stay on the experimental treatment compared to the control treatment?	In the clinical trial did the new treatment increase how long people stayed on treatment for?	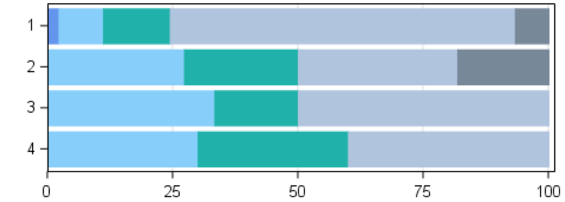

Key	1 = Patient, Carer or Member of the Public	2 = Healthcare Professional	3 = Payer or Industry Partner	4 = Statistician or Other Data Analyst	1 (Most Interested)	2	3	4 (Least Interested)	Missing

Comments from Respondents
Thirty-Three respondents provided scenarios where the question they were most interested in would change. The two main themes were:<br>• Other Effects – Explanations where the other effects of the trial treatment are brought into the question such as QoL or side effects.<br>• Context – Explanations where the context of the trial, disease area and standard of care options are considered<br>Forty-one respondents suggested additional questions, the main themes were:<br>• Post-Trial Treatment – Questions about the post-trial treatment trial participants received.<br>• Consequences – Questions around the consequences of being in the trial.<br>• Other Effects – Questions around the effect of treatment on other outcomes such as QoL or side effects.<br>• Patient Characteristics – Questions around how different patient characteristics such as age or comorbidities influence the treatment effect.

Suggestions from Researchers
From the results we think that the question about overall survival that every stakeholder group is interested in, in at least some scenarios is:<br><br>*“How does the new treatment extend survival compared to the control treatment - even though some participants stopped their trial treatment prior to death?”*<br><br>Therefore, we suggest that the project should aim to assess overall survival in full, considering participants who stop trial treatment prior to death in a way which does not:<br>• Assume no one stopped their trial treatment prior to death,<br>• Consider only those who only received their trial treatment prior to death, or<br>• Shorten overall survival to be the time on treatment.<br><br>There will be a chance to discuss this suggestion in the discussion meeting.

Information Required

The next section asked you to score whether you agreed with collecting certain pieces of information on trial participants after they have stopped their trial treatment. The table below shows the score for each piece of information by each stakeholder group. The key for the plots is at the bottom of the page.

Information	Definition	Scoring by Stakeholder Group
Date of death	The date at which a patient dies.	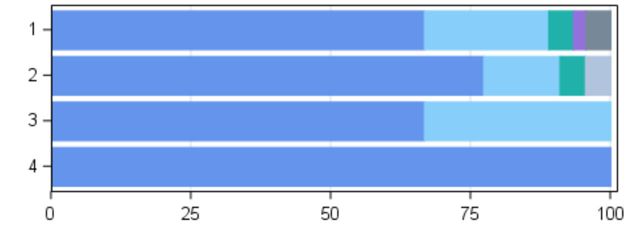
Cause of Death	The reason the patient died.	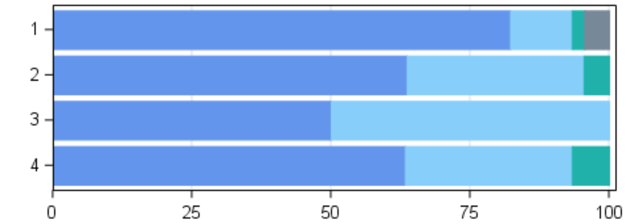
Date(s) of progression / relapse	The date the patient's cancer no longer responds to their current treatment	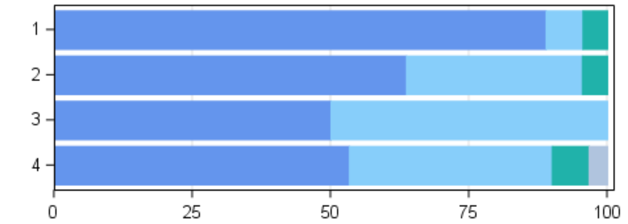
Anti-Cancer Treatment	The name of any further treatment the patient has to treat their cancer once they stopped their trial treatment and the dates they had it.	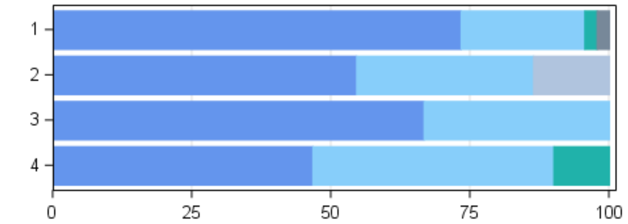
Patient Characteristics	Information about the patient such as their height and weight. Measurements which are not necessarily about their cancer.	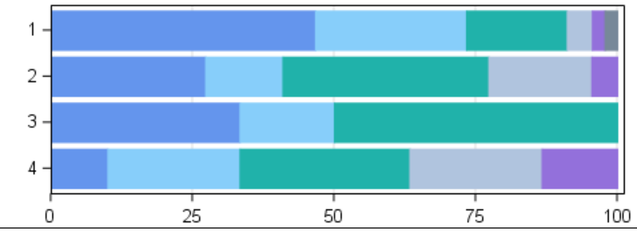
Disease Characteristics	Information about a patient's cancer such as the severity of their cancer.	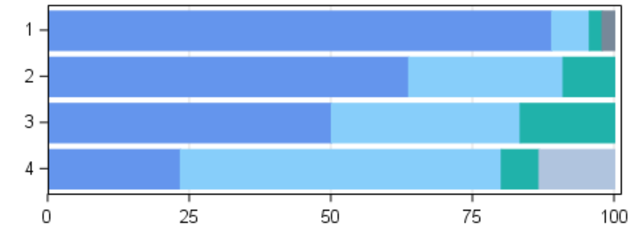

1 = Patient, Carer or Member of the Public	2 = Healthcare Professional	3 = Payer or Industry Partner	4 = Statistician or Other Data Analyst

Key

Strongly Agree	Agree	Neither Agree or Disagree	Disagree	Strongly Disagree	Missing

Comments from Respondents
Thirty respondents provided additional comments:<br>• Patients, Carers, and Members of the Public focused on how all information could be relevant with regular data collection considered acceptable if pre-specified.<br>• Statisticians and Other Data Analysts focused on the practicalities of collecting data long term and making sure what we mean by "progression" is clearly defined.<br>Forty-one respondents suggested additional data which could be collected the main types of information were:<br>• Quality of Life / Psychological Data<br>• Toxicity<br>• Co-Morbidities / Concurrent Treatment

The questionnaire also asked how this information should be collected and recorded.

How should information be collected from patients who have stopped their trial treatment?

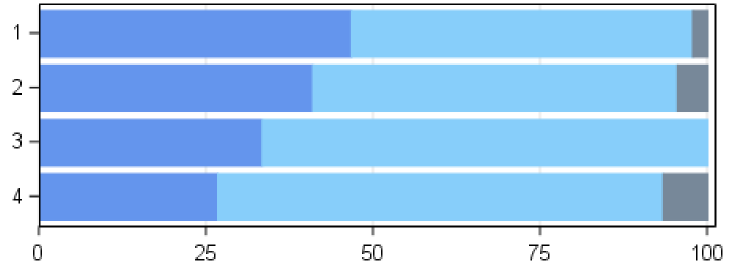

Main Reasons Provided in Favour of a Trial Follow-up Appointment

- Trial Focus - The participants' explanation mentions that a trial follow-up appointment is conducted by the research team so will keep focus on trial and ensure data collection is completed.
- Phone preference - The participants' explanation mentions that a phone appointment is preferable to a face-to-face appointment to reduce burden on patient.
- Personal Experience - The participants' explanation includes reference to their own opinion and experience of trials.

Main Reasons Provided in Favour of using a Routine Follow-up Appointment

- Patient Burden - The participants' explanation includes reducing the burden and requirement on patients to attend hospital visits in order to improve compliance.
- Context - The participants' explanation notes that the regularity of follow-up appointments would influence their decision on a trial-by-trial basis.
- Logistics - The participants' explanation includes a consideration of how this would work in practice.

How should this information be recorded and returned to the trial researchers?

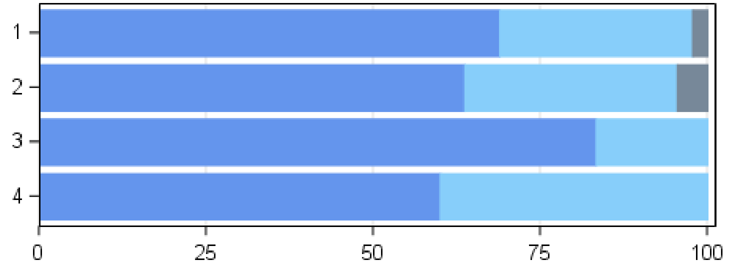

Main Reasons Provided in Favour of a Trial Database

- Standardised Data Collection - The reasoning includes that the required data is collected reliably and consistently in a controlled way to allow for it to be as accurate as possible to ensure a robust analysis.
- Routine Data Access - The reasoning includes a comment around the barriers to accessing data from routine data sources.

Main Reasons Provided in Favour of a Standard Database

- Site burden - The participants' explanation includes a comment about reducing the pressure on site staff to input data.
- Duplication - The participants' explanation includes a comment around reducing duplication across databases.
- Data Sharing - The participants' explanation favours data sharing between general practice and researchers.

Key	1 = Patient, Carer or Member of the Public	2 = Healthcare Professional	3 = Payer or Industry Partner	4 = Statistician or Other Data Analyst	Missing

Suggestions from Researchers
From the results we believe that there is broad agreement that the pieces of information which were listed could be collected on trial participants after they have completed their trial treatment, if they were needed for statistical analysis.<br><br>However, there is some disagreement around Patient characteristics and some questionnaire respondents suggested some additional pieces of information which we need to consider.<br><br>Therefore, we would like to discuss the following at the meeting:<br>• If required for trial analysis, is it acceptable to collect the following pieces of information post-trial treatment?<br>o Patient characteristics: Information about the patient such as their age, weight, height, other health conditions (co-morbidities) and other treatments (concurrent treatments). Measurements which are not necessarily linked to their cancer.<br>o Future Trial Participation: Information about any trials the patient enters for future lines of treatment such as trial name, identification number and randomised treatment.<br>o Quality of Life / Psychological Data: Information from a patient perspective such as their treatment side effects and feelings.<br>o Toxicity Data: Information about any side effects of their cancer or treatment that the patient experiences.<br><br>There is also a difference of opinion of how this data should be recorded and collected. Therefore, we would suggest that the discussion groups consider the reasons provided for each opinion and discuss:<br>• How information should be collected and recorded based on what is currently feasible.<br>• What the research community should work towards in terms of collecting and recording information.<br>• Whether time since trial treatment would change your answer.

Statistical Assumptions

The next section asked you to score whether you thought the commonly used statistically assumptions were appropriate in all, some, or no scenarios. The table below shows each assumption, how it was scored, the reasoning given by respondents the key for the plots is at the bottom of the page.

Assumption	Definition	Score	Main Reasoning
Non-Informative Censoring (Lost-To-Follow-up)	If we cannot find out information about a participant in the clinical trial at a certain time, their risk of dying is the same as a participant in the trial who we can find out information about.	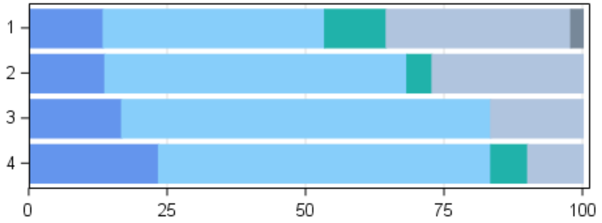	•Context – The explanation given focuses on the context of why participants were lost to follow-up or how it differed between the arms.<br>•Robustness – The explanation has a negative view of assumptions in terms of how reliable the trial results are.
Non-Informative Censoring (Stopped Trial Treatment)	If a participant stops their trial treatment at a certain time, then they are alike (except for their next treatment) to all participants who could stop their trial treatment at the same time but do not.	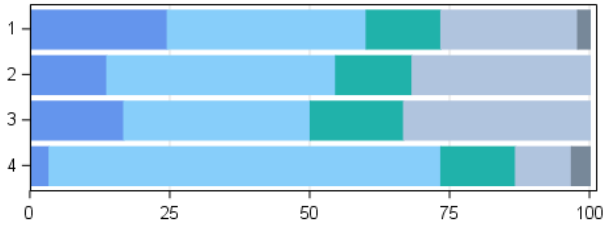	•Reason – The explanation given focuses on the reason for stopping treatment.<br>•Duration – The explanation given focuses on the duration of treatment and how that will affect response.<br>•Future – The explanation given focuses on what happens after stopping treatment and how that would affect survival.
Proportional hazards	At all times when information is being collected in the clinical trial. The chance of a participant on the current treatment (control group) dying is a multiple of the chance of a participant on the new treatment (experimental group) dying.	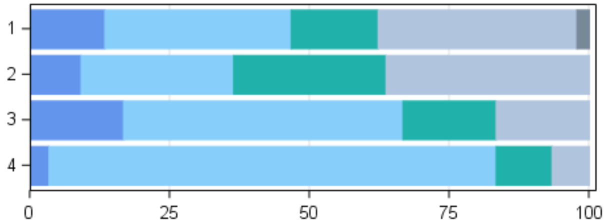	• Scenarios – The explanation given provides examples when the assumption will or will not hold.<br>• Test – The explanation given focuses on the testing of the assumption.
Common Treatment Effect	If a participant receives the experimental treatment later on in their treatment pathway, it will have the same effect as it would have done if they had it at the start of the clinical trial.	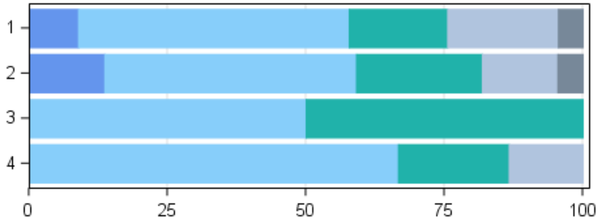	•Disease Burden – The explanation focuses on how the stage of disease may affect how effective a treatment is.<br>• Timing – The explanation focuses on how the timing of a treatment may affect how effective it is.

Key	1 = Patient, Carer or Member of the Public	2 = Healthcare Professional	3 = Payer or Industry Partner	4 = Statistician or Other Data Analyst	All Scenarios	Some Scenarios	No Scenarios	Unsure	Missing

No Unmeasured Confounding	We know everything that could affect whether a patient stops their trial treatment, and whether they will die.	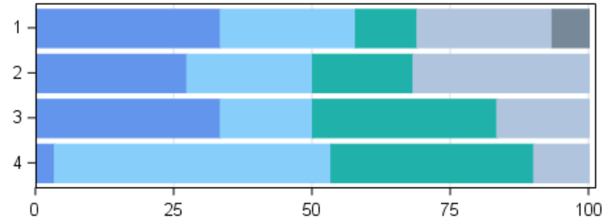	•Data Collection – The explanation given focuses on how data collection will determine whether this assumption is appropriate.<br>•Misunderstanding – The explanation given suggests that the participant did not understand the question.
No Time Dependent Co-variates	All person and disease characteristics are collected once and do not change over time.	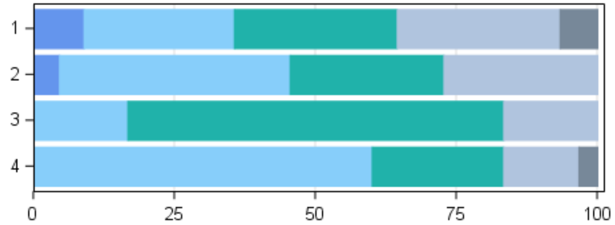	•Factors – The explanation given identifies factors which could change over time.<br>•Relevance – The explanation given focuses on how relevant considering time dependent covariates are and whether they would improve the analysis.
No Competing Risks	The reason why a participant dies is not important. The fact that they have died is the only thing that matters.	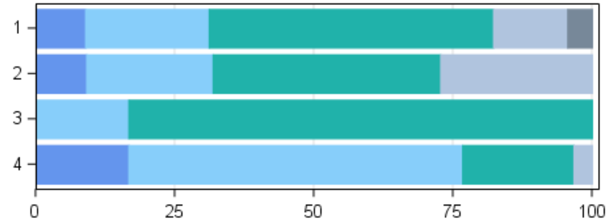	•Definition – The explanation given questions whether we are analysing disease-specific survival or overall survival.<br>•Context – The explanation given focuses on the context of the disease / trial in question.

Suggestions from the Researchers

From the results it is clear that the respondents understood that assumptions will not be applicable in every scenario. However, there are some which they preferred compared to others. From this we would like to suggest that in the development of our new methodology:

- We are clear about the assumptions we have to make and ensure they can be tested.
- We are cautious about using:
  - No Unmeasured Confounding (Stopped Trial treatment)
  - Proportional Hazards
  - Common Treatment Effect

and ensure that if assumed, the impact of No Unmeasured Confounding is assessed as part of model development.

These results and suggestions are only included for your information. They will not be discussed in the meeting. They were discussed with the Stakeholder Advisory Group and agreed based on the reasoning that Statisticians are cautious about using assumptions so would not benefit from future discussion.

Key	1 = Patient, Carer or Member of the Public	2 = Healthcare Professional	3 = Payer or Industry Partner	4 = Statistician or Other Data Analyst	All Scenarios	Some Scenarios	No Scenarios	Unsure	Missing

Presentation of Results

The next section asked you to score whether you found the ways we can present and support overall survival helpful in understanding the results. The table below shows the score for each method by each stakeholder group.

<table>
<tr><th>Presentation Method</th><th>Definition</th><th>Scoring by Stakeholder Group</th></tr>
<tr><td>Survival Curve</td><td>A picture showing how the proportion of participants alive on each treatment changes over time.</td><td>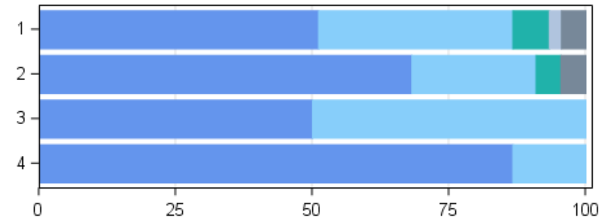
</td></tr>
<tr><td>Median Survival</td><td>The time when half of the people who were assigned to have each treatment were still alive, the other half having passed away.</td><td>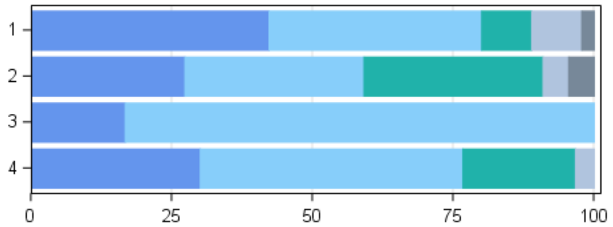
</td></tr>
<tr><td>Mean Survival</td><td>The average time that the people who were assigned to have each treatment were still alive.</td><td>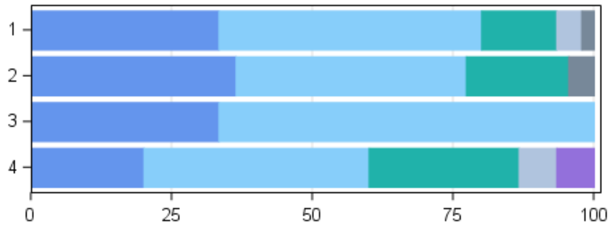
</td></tr>
<tr><td>Hazard Ratio</td><td>A ratio comparing the risk of a participant passing away on the new treatment compared to the other treatment.</td><td>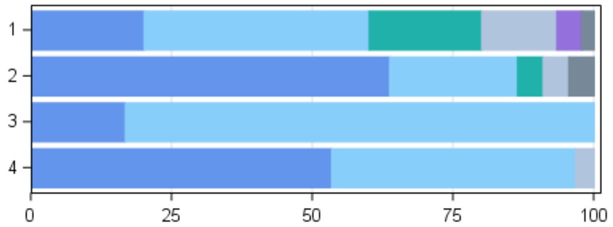
</td></tr>
<tr><td>Confidence Interval</td><td>A range of answers for which the true answer lies within at a percentage of uncertainty.</td><td>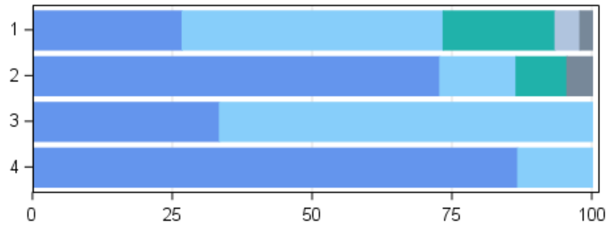
</td></tr>
<tr><td>P-Value</td><td>The probability that the answer is due to chance.</td><td>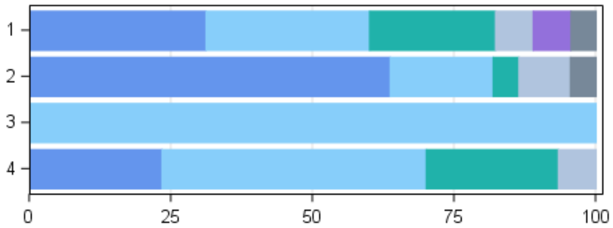
</td></tr>
</table>

<table>
<tr><td colspan="2">1 = Patient, Carer or Member of the Public</td><td colspan="2">2 = Healthcare Professional</td><td>3 = Payer or Industry Partner</td><td>4 = Statistician or Other Data Analyst</td></tr>
<tr><td colspan="6">Key</td></tr>
<tr><td></td><td></td><td></td><td></td><td></td><td></td></tr>
<tr><td>Very Helpful</td><td>Helpful</td><td>Neither Helpful or Unhelpful</td><td>Unhelpful</td><td>Very Unhelpful</td><td>Missing</td></tr>
</table>

Comments from Respondents
There were no obvious themes from the respondents, some key comments from Patients, Carers and the Public were:<br>• A preference for visual representation of the results<br>• A preference for a median over a mean<br>• A concern over the use of the term "Hazard"<br>• An interest seeing everything, provided that it is well explained<br>• A need for consistency across studies<br>The other stakeholder groups commented:<br>• The best way to present the data will depend on the disease and treatment in question<br>• Mean Survival is important but comes with limitations

Suggestions from the Researchers
In this work we suggest that we should aim to standardise in part how trials should present the impact of post-trial treatment on the analysis of overall survival in a clearly explained scenario. We suggest that what we use should aim to include:<br>• A visual representation<br>• A summary statistic, perhaps in terms of a median<br>• A hazard ratio supported by at least a confidence interval. Where the language around the hazard ratio is considered when fed back to patients and the public.<br><br>These results and suggestions are included for your information. They will not be discussed in the meeting. However, once the project has developed a new way to assess survival in clinical trials you will be invited to comment on how it is presented and explained to each stakeholder groups.

Discussion Group Meeting Plan
**Welcome, Introduction and Aim (5 Minutes)**
Kara-Louise Royle will welcome everyone to the meeting. Ask everyone to introduce themselves and remind everyone of the aim and format of the session.
**Summary of Respondents (5 Minutes)**
Kara-Louise Royle will present the demographics of those who completed the online questionnaire
**Main Discussion**
• *Kara-Louise Royle will present the questionnaire results where consensus not reached or was reached but further discussion was thought beneficial*<br>• *After each presentation breakout groups will be created for attendees to discuss their opinions within their stakeholder group and across the different stakeholder groups*<br>• *Each breakout group will be 10 minutes long*<br>• *After each discussion there is the opportunity to feedback key discussion points using Padlet*<br>• *Individual handouts have been created to support note taking and discussions and are provided at the end of the pre-meeting document.*
**Discussion 1 (30 Minutes)**
What are your thoughts on the decision for the research to consider the question:<br>*"How does the new treatment extend survival compared to the control treatment - even though some participants stopped their trial treatment prior to death?"*
**Break (10 Minutes)**<br>*Please add key discussion points from Discussion 1 onto the Padlet board:*<br>[redacted]
**Discussion 2 (30 Minutes)**
If required for trial analysis, is it acceptable to collect the following pieces of information post-trial treatment?<br>• Patient characteristics: Information about the patient such as their age, weight, height, other health conditions (co-morbidities) and other treatments (concurrent treatments). Measurements which are not necessarily linked to their cancer.<br>• Future Trial Participation: Information about any trials the patient enters for future lines of treatment such as trial name, identification number and randomised treatment.<br>• Quality of Life / Psychological Data: Information from a patient perspective such as their treatment side effects and feelings.<br>• Toxicity Data: Information about any side effects of their cancer or treatment that the patient experiences.
**Break (10 Mins)**<br>*Please add key discussion points from Discussion 2 onto the Padlet board:*<br>[redacted]
**Discussion 3 (30 Minutes)**
How should data be collected and recorded on a trial participant who has stopped their trial treatment?<br>•Given what is currently possible<br>•In an ideal world<br>Would time since trial treatment change your answer?
**Break (10 Mins)**<br>*Please add key discussion points from Discussion 3 onto the Padlet board:*<br>[redacted]
**Closing Discussion (20 Minutes)**
A chance for anyone to raise any points with the entire group. Kara-Louise will then go through the next steps and post-meeting actions. This includes the post-meeting questionnaire which will go live after the meeting. You will receive an email which you can use to access the questionnaire.
**Meeting Close**

Discussion Group Handouts

Discussion 1:

What are your thoughts on the decision for the research to consider the question:

"How does the new treatment extend survival compared to the control treatment - even though some participants stopped their trial treatment prior to death?"

My thoughts on this point are:	
Notes from the first breakout group:	
Notes from the second breakout group:	

Discussion 2:

If required for trial analysis, is it acceptable to collect the following pieces of information post-trial treatment?

- Patient characteristics: Information about the patient such as their age, weight, height, other health conditions (co-morbidities) and other treatments (concurrent treatments). Measurements which are not necessarily linked to their cancer.
- Future Trial Participation: Information about any trials the patient enters for future lines of treatment such as trial name, identification number and randomised treatment.
- Quality of Life / Psychological Data: Information from a patient perspective such as their treatment side effects and feelings.
- Toxicity Data: Information about any side effects of their cancer or treatment that the patient experiences.

	Patient characteristics	Future Trial Participation:	Quality of Life / Psychological Data	Toxicity Data:
My thoughts on this point are:				
My notes from the first breakout group:				
My notes from the second breakout group:				

Discussion 3:

How should data be collected and recorded on a trial participant who has stopped their trial treatment?

•Given what is currently possible
•In an ideal world

Would time since trial treatment change your answer?

**Collecting Data (Trial Follow-Up OR Routine Appointment)**	Currently Possible	In an Ideal World	Would time since trial treatment change your answer?
My notes on this point are:			
My notes from the first breakout group:			
My notes from the second breakout group:			

**Recording Data (Trial Database OR Standard Database)**	Currently Possible	In an Ideal World	Would time since trial treatment change your answer?
My thoughts on this point are:			
Notes from the first breakout group:			
Notes from the second breakout group:			
