# Supplementary material for: How should overall survival be analysed in randomised clinical trials in cancer if participants receive subsequent treatment lines? A stakeholder consultation
Source: Trials. 2025 Oct 24;26:434. doi: 10.1186/s13063-025-09148-3 (PMC12551141; doi:10.1186/s13063-025-09148-3)
Supplement: Supplementary file 6 — Additional File 6: Post-Focus group online questionnaire. [file 13063_2025_9148_MOESM6_ESM.pdf]

SOLVE Post-Discussion Groups V1 20230116

Questionnaire Access

Please enter your email address ✱ *Required*

Introduction

Introduction

Thank you for attending the discussion meeting for the SOLVE study.

Following your discussions in the meeting please answer the questions on the following page.

If at any point you have any questions, please contact Kara-Louise Royle at SOLVE@leeds.ac.uk.

Data Protection

The following sections answer some of the questions you may have around what happens to your data collected during the research. The research will be GDPR compliant, the University of Leeds privacy notice can be found here: https://dataprotection.leeds.ac.uk/wp-content/uploads/sites/48/2019/02/Research-Privacy-Notice.pdf. The university also has a data protection officer who can be contacted at: dpo@leeds.ac.uk.

How will my data be protected?

Less info

Any data collected on this research will be stored securely on either the online survey database or on University of Leeds network computers with access restricted to the research team. Any results or will be presented in summaries, conference presentations and journal articles in such a way that you cannot be identified.

Click on More info to see the answer

Who has organised and sponsored the research?

Less info

The research is being organised and coordinated by the Leeds Institute of Clinical Trials Research at the University of Leeds, who is sponsoring the research.

Click on More info to see the answer

Who has reviewed the research?

Less info

The research was reviewed and funded by the NIHR (National Institute of Health Research) under their Doctoral Fellowship funding stream. It has also been reviewed by the Chief Investigator's PhD supervisors. Finally, it has been reviewed and approved by an NHS Research Ethics Committee (22/YH/0155).

Click on More info to see the answer

How long will you keep my data?

Less info

Data will be kept for the length of the research (5 years) and archived on university systems for an additional 15 years as per sponsor requirements.

Click on More info to see the answer

Discussion Group Questions

Discussion 1

The following question relates to Discussion 1 from the discussion group meeting.

What are your thoughts on the decision for the research to consider the question: "*How does the new treatment extend survival compared to the control treatment - even though some participants stopped their trial treatment prior to death?*" Suggesting that the project should aim to assess overall survival in full, considering participants who stop trial treatment prior to death in a way which does not: ·Assume no one stopped their trial treatment prior to death, ·Consider only those who only received their trial treatment prior to death, or ·Shorten overall survival to be the time on treatment. ·This prioritises the first question and leaves the other three questions as potential extensions if there is time in the project to address them.

Discussion 2

The following questions relate to Discussion 2 from the discussion group meeting.

To answer research questions accurately, we need to collect information on patients in a clinical trial. Listed below are some of the pieces of information we may need to collect to find out if the new treatment extended patient’s lives. Please read the descriptions and score whether you agree or disagree that it is reasonable to collect this information on patients who have taken part in a clinical trial. After they have stopped receiving their trial treatment.

	Strongly Agree	Agree	Neither Agree or Disagree	Disagree	Strongly Disagree
Patient Characteristics: Information about the patient such as their age, weight, height, other health conditions (co-morbidities) and other treatments (concurrent treatments). Measurements which are not necessarily linked to their cancer.	○	○	○	○	○
Future Trial Participation: Information about any trials the patient enters for future lines of treatment such as trial name, identification number and randomised treatment.	○	○	○	○	○
Quality of Life / Psychological Data: Information from a patient perspective such as their treatment side effects and feelings.	○	○	○	○	○
Toxicity Data: Information about any side effects of their cancer or treatment that the patient experiences.	○	○	○	○	○

Discussion 3

The following questions relate to Discussion 3 from the discussion group meeting.

There are two different ways this information can be collected from patients who have stopped their trial treatment.

In the current research world, which do you think is most appropriate?

- ○ At a trial follow-up appointment. This appointment could be in person or over the phone and would be an additional appointment to the ones the patient was attending as part of their off-trial treatment.
- ○ At a routine appointment. This appointment could be in person or over the phone and would be an appointment which the patient attended as part of their off-trial treatment.

What about in an ideal world?

- ○ At a trial follow-up appointment. This appointment could be in person or over the phone and would be an additional appointment to the ones the patient was attending as part of their off-trial treatment.
- ○ At a routine appointment. This appointment could be in person or over the phone and would be an appointment which the patient attended as part of their off-trial treatment.

Would time since trial treatment change your answer?

There are also two different ways this information can be recorded and returned to the trial researchers.

In the current research world, which do you think is most appropriate?

- ○ From a database which was made specifically for the trial. This makes sure everything is recorded in the same way.
- ○ From a database which is completed normally as part of standard practice. This means that information isn’t duplicated across databases.

What about in an ideal world?

- ○ From a database which was made specifically for the trial. This makes sure everything is recorded in the same way.
- ○ From a database which is completed normally as part of standard practice. This means that information isn't duplicated across databases.

Would time since trial treatment change your answer?

Final page

Thank you for completing the post-meeting questionnaire.
