# Supplementary material for: How should overall survival be analysed in randomised clinical trials in cancer if participants receive subsequent treatment lines? A stakeholder consultation
Source: Trials. 2025 Oct 24;26:434. doi: 10.1186/s13063-025-09148-3 (PMC12551141; doi:10.1186/s13063-025-09148-3)
Supplement: Supplementary file 7 — Additional File 7: Supplementary tables and figures. [file 13063_2025_9148_MOESM7_ESM.docx]

## Additional File 7 – Supplementary Tables and Figures

**Contents**

[Supplementary Table 1: Recipients of Targeted Emails 2](#_Toc191538658)

[Supplementary Table 2: Summary of Invited Respondents 5](#_Toc191538659)

[Supplementary Figure 1: Flow chart of implemented methods 8](#_Toc191538660)

[Supplementary Figure 2: Clinical Trials Tweetorial 9](#_Toc191538661)

[Supplementary Figure 3: Statistics Tweetorial 10](#_Toc191538662)

[Supplementary Figure 4: Survival Analysis Tweetorial 11](#_Toc191538663)

[Supplementary Figure 5: Summary of Statistical Assumptions Results 13](#_Toc191538664)

[Supplementary Figure 6: Summary of Presentation of Results 15](#_Toc191538665)

[Supplementary Figure 7: Post-Focus Group Questionnaire Results – Data Items 17](#_Toc191538666)

[Supplementary Figure 8: Post-Focus Group Questionnaire Results – Collecting Information 20](#_Toc191538667)

[Supplementary Figure 9: Post-Focus Group Questionnaire Results – Recording Information 21](#_Toc191538668)

Supplementary Table 1: Recipients of Targeted Emails

| Target Stakeholder Group | A member of the public / patient or carer | Health Professional | Industry Partner / Payer | Statistician or other data analyst |
| --- | --- | --- | --- | --- |
| Organization / Mailing List |  |  |  |  |
| **Allstat**  A mailing list for statisticians and data scientists |  |  |  | ✓ |
| **AMRC (Association of Medical Research Charities)**  A membership organisation of the leading medical and health research charities in the UK |  | ✓ |  |  |
| **APBI (Association of the British Pharmaceutical Industry)**  An organisation which represents companies who invest in drug development. |  |  | ✓ |  |
| **Association of Lead Research Nurses (Research and Innovation Bulletin)**  A network of research active nurses in the UK. |  | ✓ |  |  |
| **Cancer Patient and Public Involvement Group Leeds**  A patient and public involvement group based in Leeds, UK. | ✓ |  |  |  |
| **Cancer Research UK Clinical Trial Units**  A network of clinical trials units across the UK who receive infrastructure funding from cancer research UK. | ✓ | ✓ |  | ✓ |
| **Health Data Research UK**  A charity which crosscuts industry and academia focusing on matters relating to health data science. | ✓ | ✓ |  | ✓ |
| **Health Research Board Trials Methodology Research Network (Ireland)**  A network of researchers based in Ireland focusing on trials methodology. |  |  |  | ✓ |
| **HESG (Health Economists’ Study Group) Email List**  A mailing list of all members signed up to the HESG. |  |  |  | ✓ |
| **HRA (Health Research Agency)**  An organisation ensuring that health and social care research protects the interests of patients and the public. |  |  | ✓ |  |
| **Independent Cancer Patient Voice**  A patient advocate group supporting involvement in research, | ✓ |  |  |  |
| **Lymphoma Action**  A UK charity dedicated to supporting patients with lymphoma | ✓ |  |  |  |
| **Marie Curie**  A UK charity dedicated to supporting patients at end of life care. | ✓ |  |  |  |
| **Mednet**  A University of Leeds mailing list for everyone in medicine and health. | ✓ | ✓ | ✓ | ✓ |
| **Myeloma UK**  A UK charity dedicated to supporting patients with Myeloma | ✓ |  |  |  |
| **NCRI (National Cancer Research Institute)**  A network of researchers and patients to support the advancement of cancer research. | ✓ | ✓ | ✓ | ✓ |
| **NHS Digital**  An organisation which focuses on the sharing of health systems data. |  |  | ✓ |  |
| **SOLVE PhD Supervisors**  A group of three statisticians and an health economist spanning industry and academia. |  |  |  | ✓ |
| **PSI (Statisticians in the Pharmaceutical Industry)**  A network of statisticians and other data analysts working in medical research both in industry and academia. |  |  |  | ✓ |
| **RADAR Patient Advisory Group**  A group of six patients and carers who provide PPI for the Myeloma trial RADAR run by Leeds CTRU. | ✓ |  |  |  |
| **Registered Clinical Trials Unit (CTU) Network**  A network of all of the registered CTUs in the UK | ✓ | ✓ | ✓ | ✓ |
| **Royal Colleges**  A membership organisation for the 23 medical royal colleges and faculties focusing on the different specialities in the UK. |  | ✓ |  |  |
| **Royal Statistical Society**  A membership organisation for statisticians |  |  |  | ✓ |
| **SOLVE Stakeholder Advisory Group**  A group of 2 patient representatives, 2 XXX and 2 clinicians. | ✓ | ✓ |  | ✓ |
| **Trial Managers’ Network**  A network of trial managers who work in clinical trials in the UK. | ✓ |  |  |  |
| **Trials Methodology Research Partnership (UK)**  A network of institutions and organisations who work in trial methodology. | ✓ | ✓ | ✓ | ✓ |
| **Yorkshire Cancer Community**  A charity focused on supporting people in Yorkshire who have cancer. | ✓ |  |  |  |
| **Cancer Drugs Fund Team**  An organisation focused on facilitating early access to effective treatments in the UK |  | ✓ | ✓ |  |

Supplementary Table 2: Summary of Invited Respondents

|  | **Attended (n=6)** | **Declined (n=8)** | **Did Not Attend (n=5)** | **Not Invited (n=14)** | **Total**  **(n=33)** |
| --- | --- | --- | --- | --- | --- |
| **How old are you?** |  |  |  |  |  |
| Median (IQR) | 58.00  (37.00, 62.00) | 46.50  (39.00, 54.00) | 55.00  (50.00, 55.00) | 60.50  (48.00, 68.00) | 55.00  (45.00, 64.00) |
| **What is your ethnic group?** |  |  |  |  |  |
| English/Welsh/Scottish/Northern Irish/British | 5 (83.3%) | 6 (75.0%) | 2 (40.0%) | 13 (92.9%) | 26 (78.8%) |
| Irish | 1 (16.7%) | 0 (0.0%) | 1 (20.0%) | 0 (0.0%) | 2 (6.1%) |
| Other, please describe | 0 (0.0%) | 1 (12.5%) | 1 (20.0%) | 0 (0.0%) | 2 (6.1%) |
| Prefer not to say | 0 (0.0%) | 1 (12.5%) | 1 (20.0%) | 0 (0.0%) | 2 (6.1%) |
| Missing | 0 (0.0%) | 0 (0.0%) | 0 (0.0%) | 1 (7.1%) | 1 (3.0%) |
| **What sex are you?** |  |  |  |  |  |
| Male | 5 (83.3%) | 3 (37.5%) | 1 (20.0%) | 3 (21.4%) | 12 (36.4%) |
| Female | 1 (16.7%) | 5 (62.5%) | 4 (80.0%) | 10 (71.4%) | 20 (60.6%) |
| Missing | 0 (0.0%) | 0 (0.0%) | 0 (0.0%) | 1 (7.1%) | 1 (3.0%) |
| **Where in the UK are you based?** |  |  |  |  |  |
| Scotland | 0 (0.0%) | 0 (0.0%) | 2 (40.0%) | 1 (7.1%) | 3 (9.1%) |
| Northern Ireland | 1 (16.7%) | 0 (0.0%) | 0 (0.0%) | 0 (0.0%) | 1 (3.0%) |
| North East England | 1 (16.7%) | 0 (0.0%) | 0 (0.0%) | 0 (0.0%) | 1 (3.0%) |
| Yorkshire and The Humber | 2 (33.3%) | 3 (37.5%) | 2 (40.0%) | 6 (42.9%) | 13 (39.4%) |
| East Midlands | 0 (0.0%) | 0 (0.0%) | 0 (0.0%) | 1 (7.1%) | 1 (3.0%) |
| West Midlands | 0 (0.0%) | 1 (12.5%) | 0 (0.0%) | 0 (0.0%) | 1 (3.0%) |
| London | 0 (0.0%) | 2 (25.0%) | 1 (20.0%) | 2 (14.3%) | 5 (15.2%) |
| South East England | 1 (16.7%) | 1 (12.5%) | 0 (0.0%) | 2 (14.3%) | 4 (12.1%) |
| South West England | 1 (16.7%) | 0 (0.0%) | 0 (0.0%) | 2 (14.3%) | 3 (9.1%) |
| Other | 0 (0.0%) | 1 (12.5%) | 0 (0.0%) | 0 (0.0%) | 1 (3.0%) |
| **Are You?** |  |  |  |  |  |
| A Member of the Public | 1 (16.7%) | 1 (12.5%) | 1 (20.0%) | 3 (21.4%) | 6 (18.2%) |
| A Patient or Carer | 1 (16.7%) | 1 (12.5%) | 1 (20.0%) | 8 (57.1%) | 11 (33.3%) |
| A Health Professional | 1 (16.7%) | 2 (25.0%) | 1 (20.0%) | 0 (0.0%) | 4 (12.1%) |
| An Industry Partner | 0 (0.0%) | 0 (0.0%) | 1 (20.0%) | 0 (0.0%) | 1 (3.0%) |
| A Payer | 0 (0.0%) | 1 (12.5%) | 0 (0.0%) | 0 (0.0%) | 1 (3.0%) |
| A Statistician or Other Data Analyst | 3 (50.0%) | 3 (37.5%) | 1 (20.0%) | 3 (21.4%) | 10 (30.3%) |
| **Have you had any prior experience of clinical trials?** |  |  |  |  |  |
| Yes | 5 (83.3%) | 7 (87.5%) | 4 (80.0%) | 9 (64.3%) | 25 (75.8%) |
| No | 1 (16.7%) | 1 (12.5%) | 1 (20.0%) | 5 (35.7%) | 8 (24.2%) |
| **Do you think it is important to consider the effect that treatment given after trial treatment has on overall survival?** |  |  |  |  |  |
| Yes | 5 (83.3%) | 5 (62.5%) | 4 (80.0%) | 13 (92.9%) | 27 (81.8%) |
| Depends | 1 (16.7%) | 3 (37.5%) | 1 (20.0%) | 1 (7.1%) | 6 (18.2%) |
| **Before today have you ever thought about how after trial treatment might affect overall survival?** |  |  |  |  |  |
| Yes | 4 (66.7%) | 7 (87.5%) | 2 (40.0%) | 8 (57.1%) | 21 (63.6%) |
| No | 2 (33.3%) | 1 (12.5%) | 3 (60.0%) | 5 (35.7%) | 11 (33.3%) |
| Missing | 0 (0.0%) | 0 (0.0%) | 0 (0.0%) | 1 (7.1%) | 1 (3.0%) |

Supplementary Figure 1: Flow chart of implemented methods


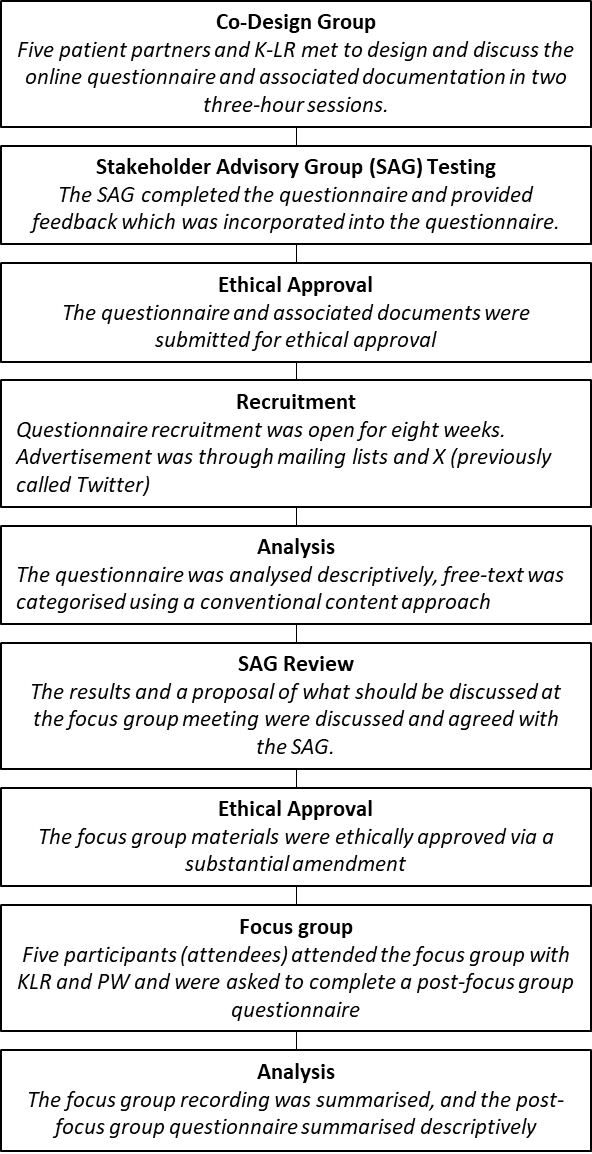


Supplementary Figure 2: Clinical Trials Tweetorial


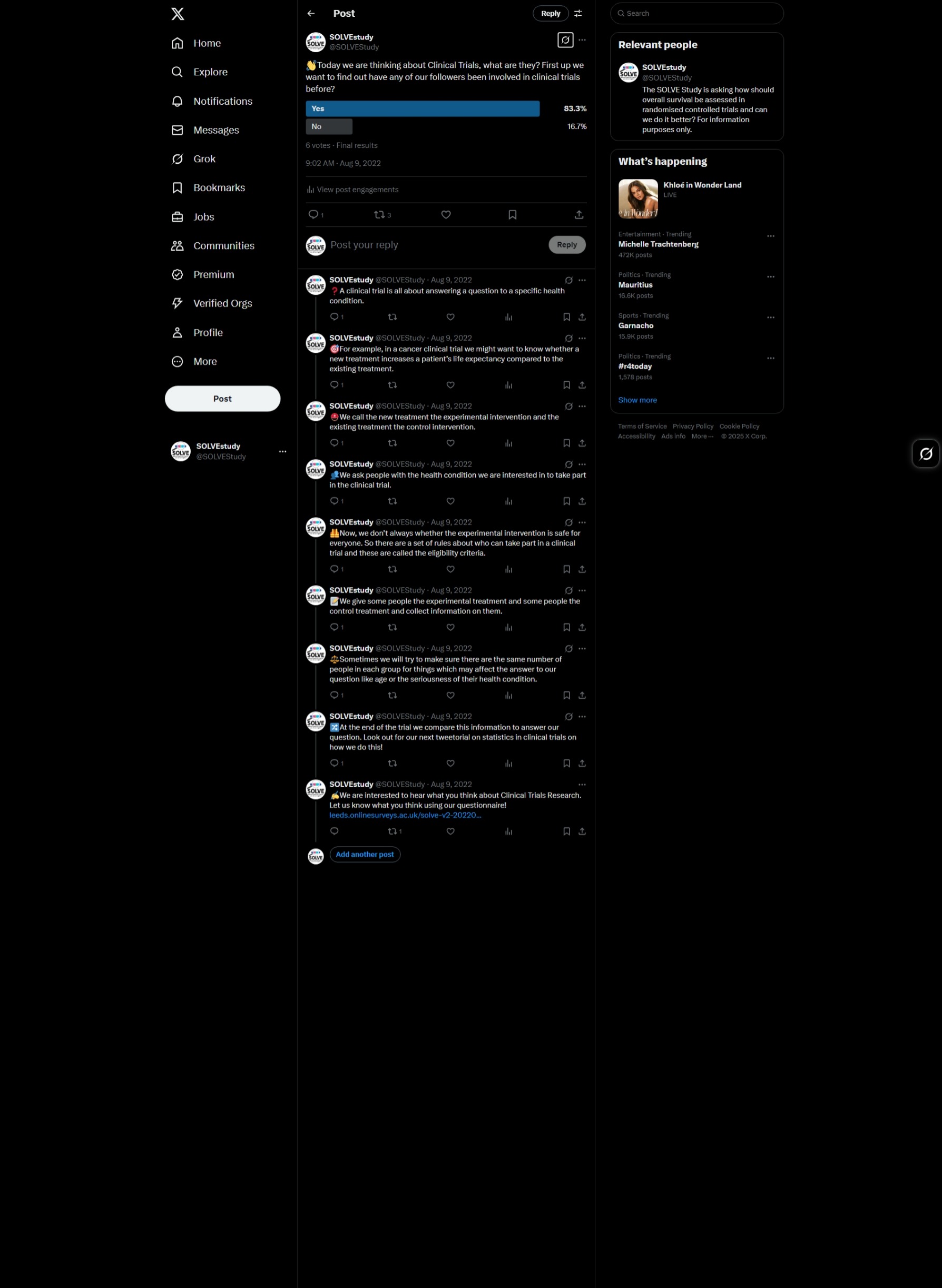


Supplementary Figure 3: Statistics Tweetorial


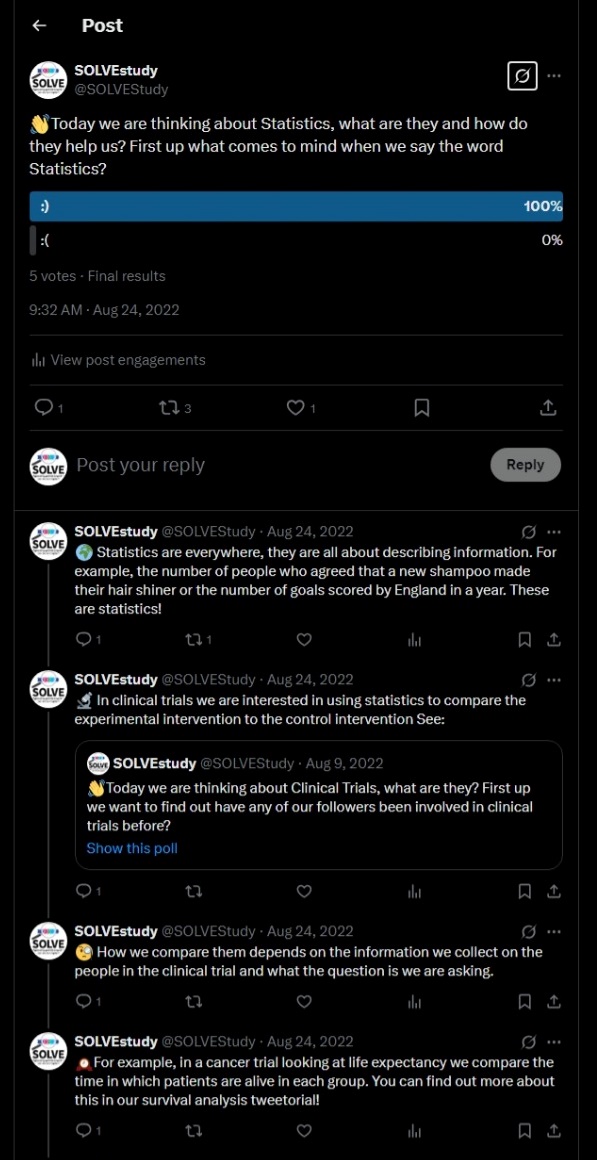

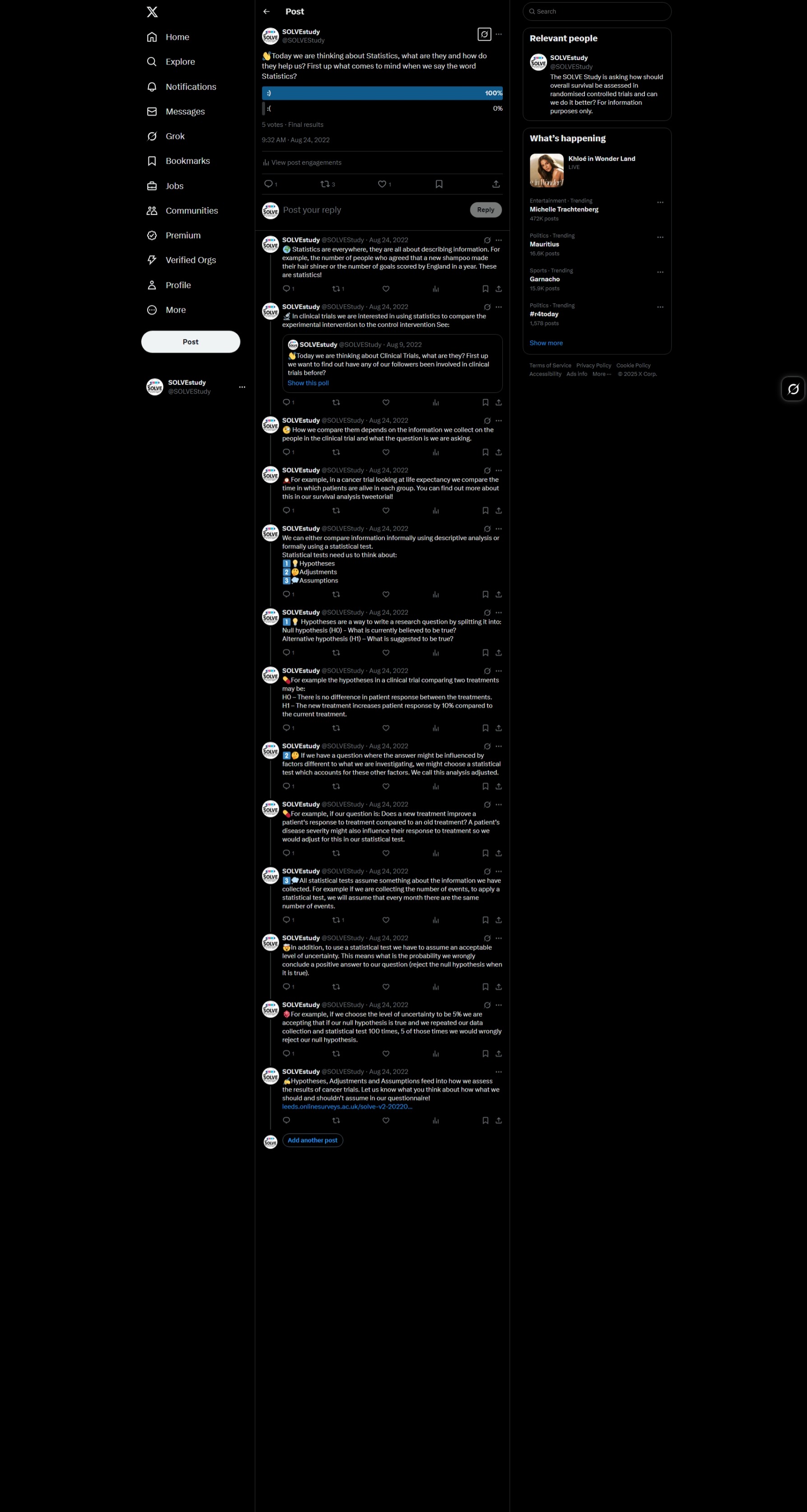


Supplementary Figure 4: Survival Analysis Tweetorial


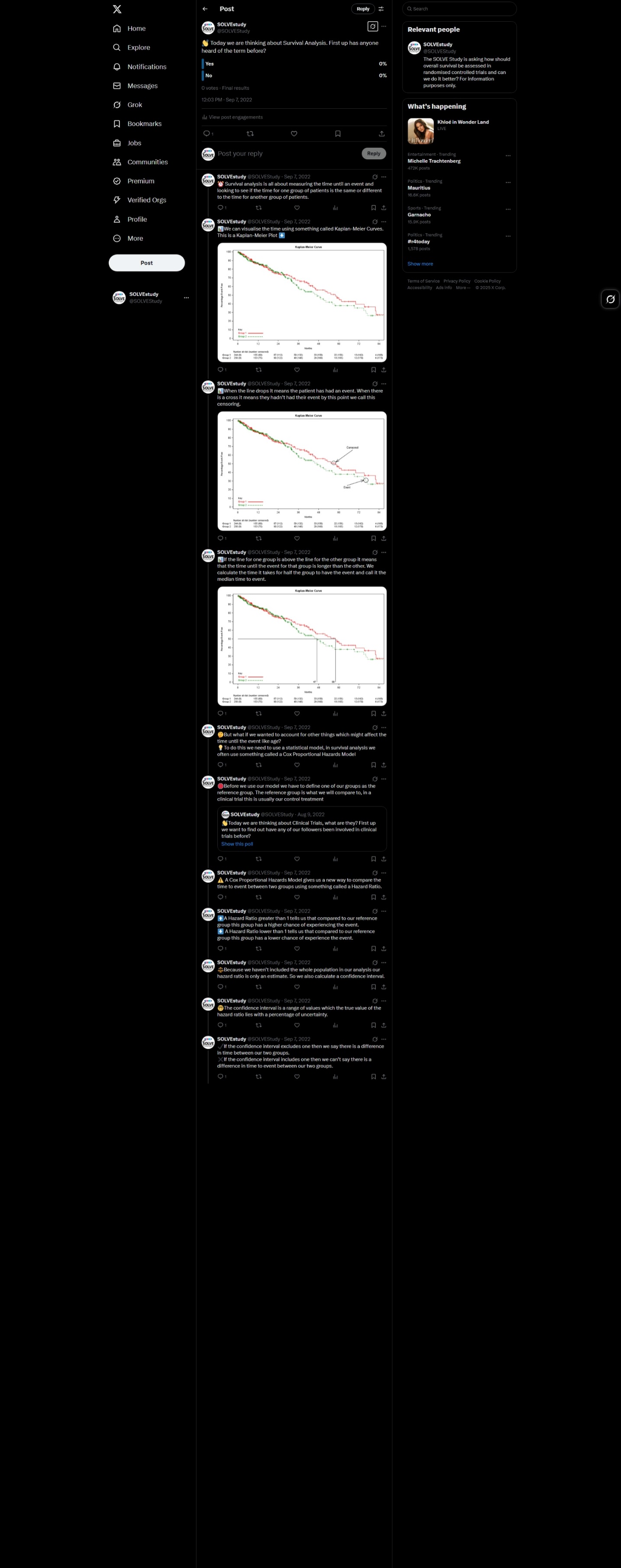

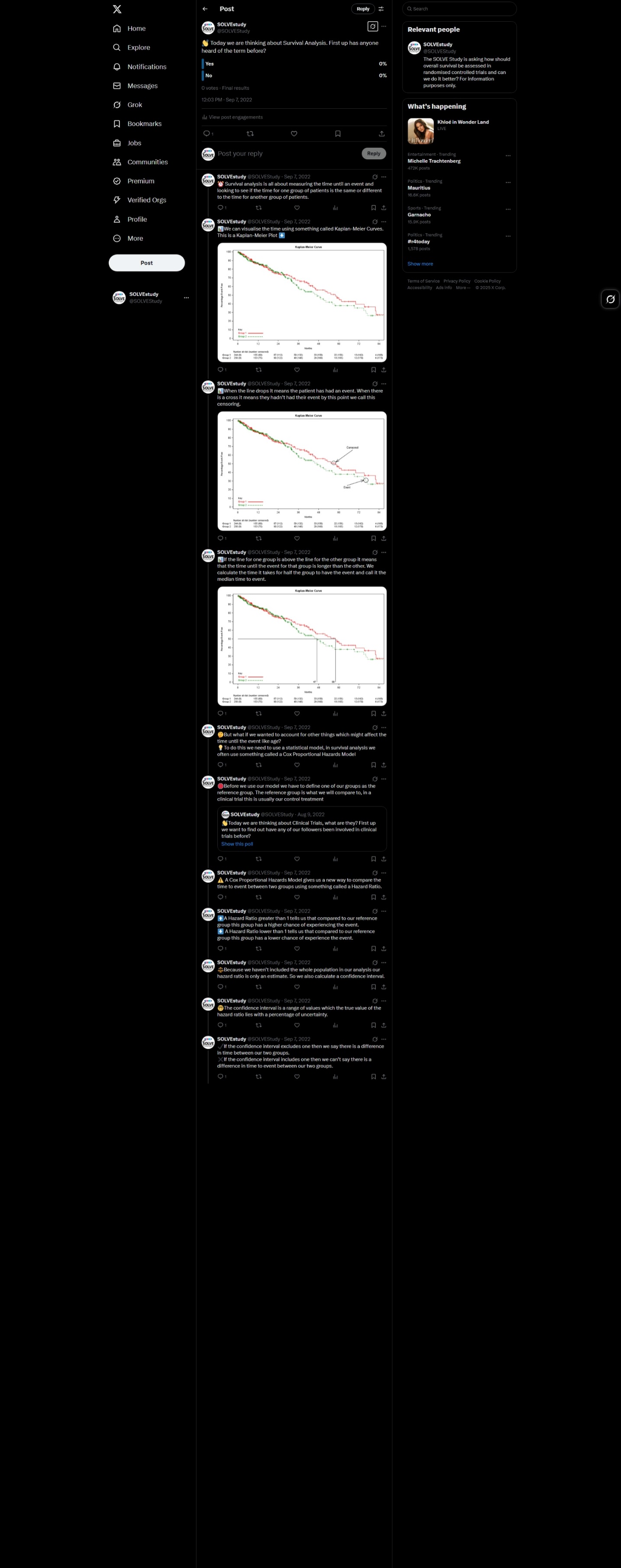

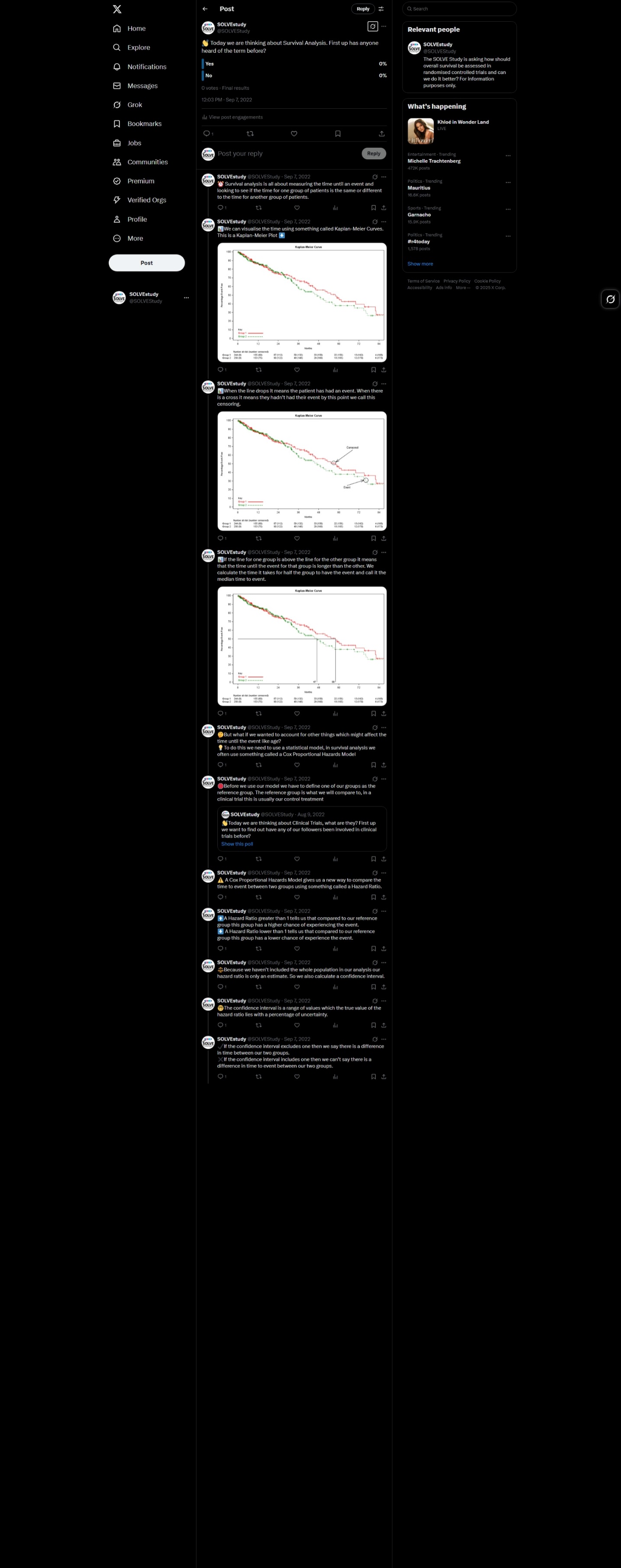


Supplementary Figure 5: Summary of Statistical Assumptions Results


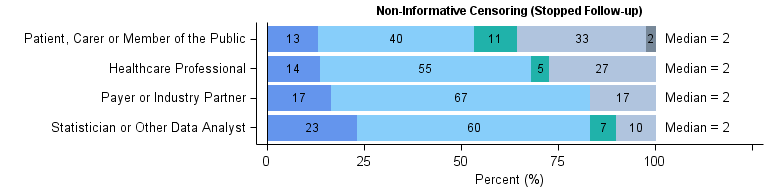

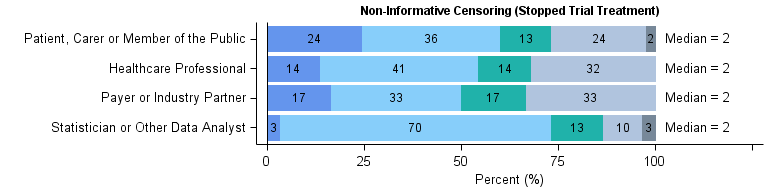

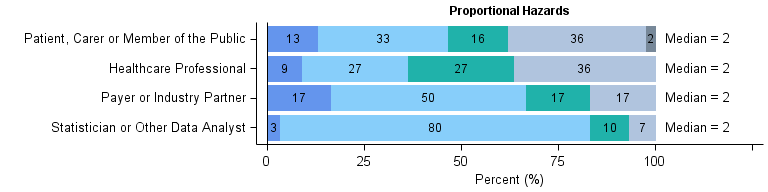

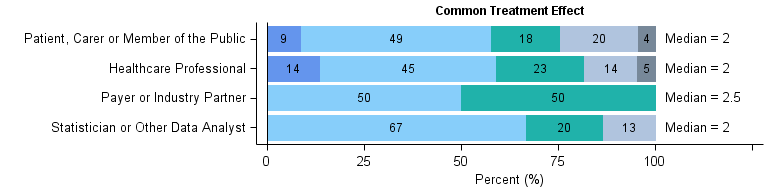

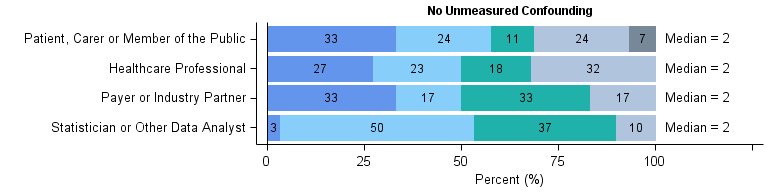

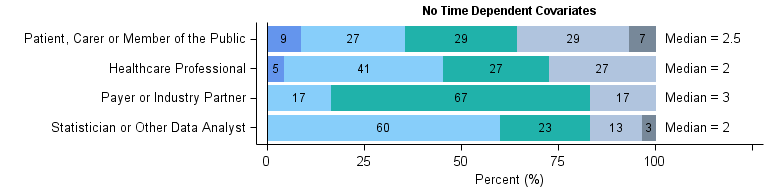

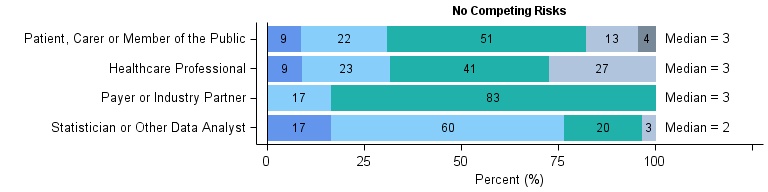


| Key | 1 | 2 | 3 | 4 | 9876 |
| --- | --- | --- | --- | --- | --- |
|  | All Scenarios | Some Scenarios | No Scenarios | Unsure | Missing |

Supplementary Figure 6: Summary of Presentation of Results


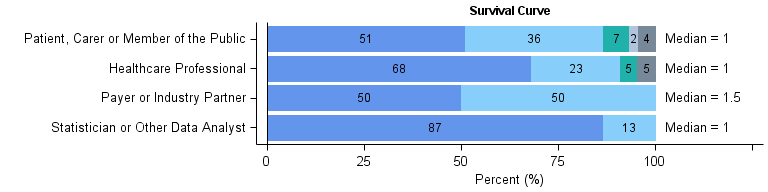

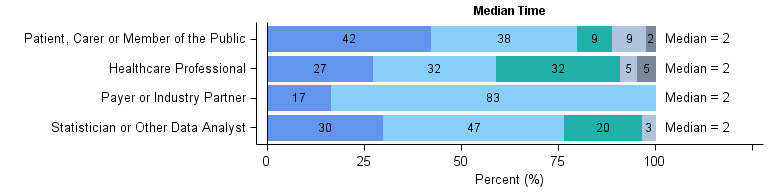

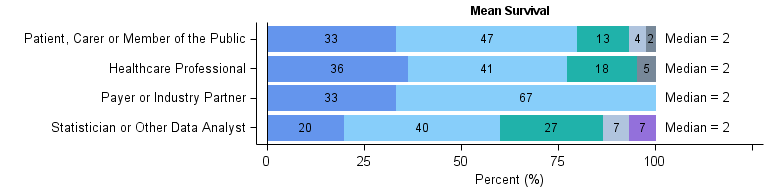

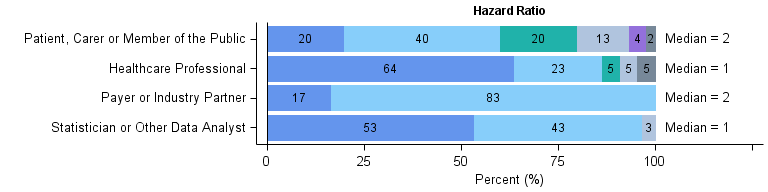

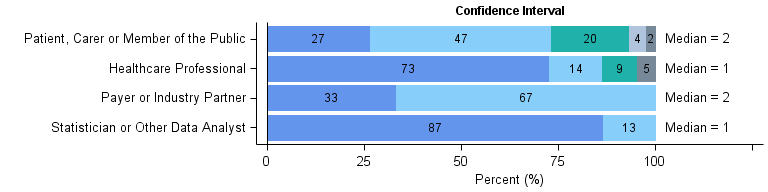

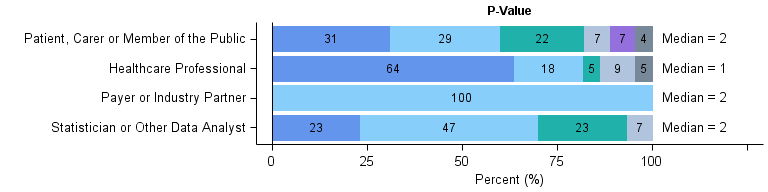


| **Key** | | | | | |
| --- | --- | --- | --- | --- | --- |
| 1 | 2 | 3 | 4 | 5 | 9876 |
| Very Helpful | Helpful | Neither Helpful or Unhelpful | Unhelpful | Very Unhelpful | Missing |

Supplementary Figure 7: Post-Focus Group Questionnaire Results – Data Items


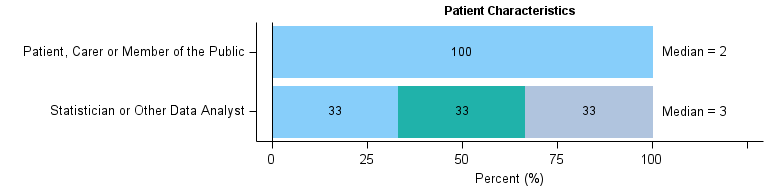


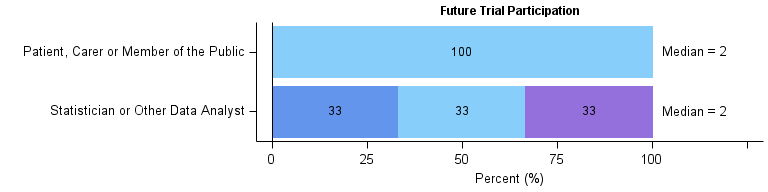


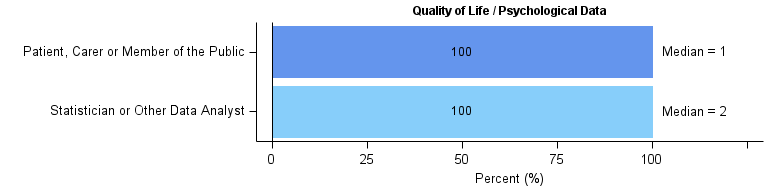


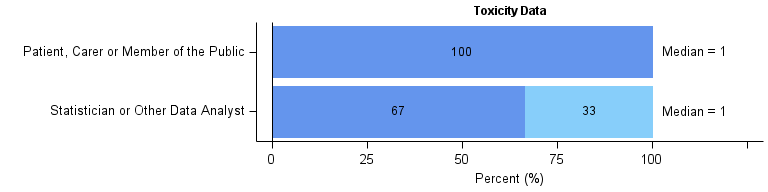


| **Key** | | | | | |
| --- | --- | --- | --- | --- | --- |
| 1 | 2 | 3 | 4 | 5 | 9876 |
| Strongly Agree | Agree | Neither Agree or Disagree | Disagree | Strongly Disagree | Missing |

Supplementary Figure 8: Post-Focus Group Questionnaire Results – Collecting Information


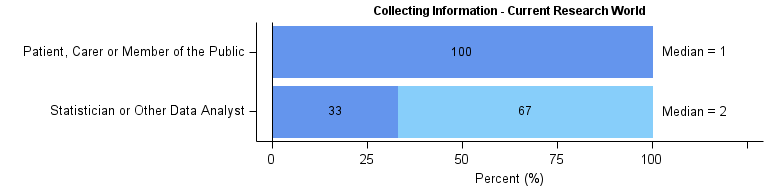


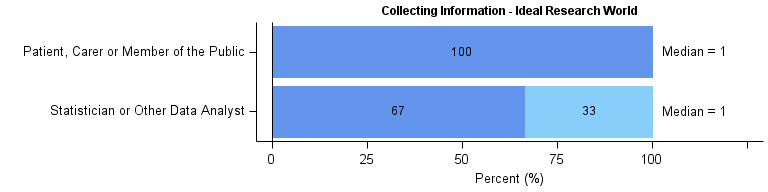


| **Key** | |
| --- | --- |
| **Colour** | **Score** |
| 1 | At a trial follow-up appointment. |
| 2 | At a routine appointment. |
| 9876 | Missing |

Supplementary Figure 9: Post-Focus Group Questionnaire Results – Recording Information


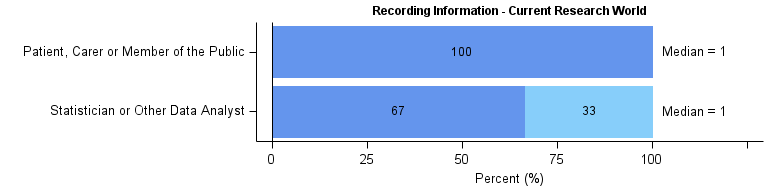


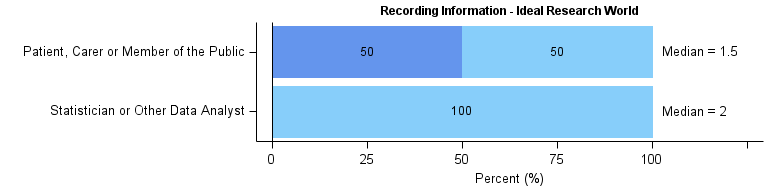


| **Key** | |
| --- | --- |
| **Colour** | **Choice** |
| 1 | From a database which was made specifically for the trial. |
| 2 | From a database which is completed normally as part of standard practice. |
| 9876 | Missing |
